# An improved enterprise development optimizer based on labor migration for numerical optimization

**DOI:** 10.1038/s41598-025-07328-4

**Published:** 2025-07-19

**Authors:** Dawei Zhao, Leidong Feng, Yijiang Wang, Xinyu Cai, Xiang Liu

**Affiliations:** 1 School of Labor Economics, Capital University of Economics and Business, Beijing, China; 2 School of Economics and Management, Shandong Youth University of Political Science, Jinan, 25000 China; 3 College of Business, Jiaxing University, Jiaxing, 314001 China; 4 School of Labor and Human Resources, Renmin University of China, Beijing, 100872 China; 5 Institute for Energy Research, Jiangsu University, Zhenjiang, 212013 China

**Keywords:** Metaheuristic, Enterprise development optimizer, Labor migration, Engineering optimization problems, Computer science, Software, Statistics

## Abstract

Enterprise Development Optimizer (EDO) is a meta-heuristic algorithm inspired by the enterprise development process. Although EDO is effective in the optimization field, it suffers from issues such as premature convergence and unequal exploration–exploitation ratio. These shortcomings restrict the performance of the algorithm in some complex problem. This research proposes an improved EDO, called LMEDO, in which EDO is integrated with incorporates time-phase based switching strategy, economy-driven guided based learning strategy and spatial selectivity-based selection strategy to improve convergence rate, stability, and search effectiveness. Among these strategies, the time-phase based switching strategy allows EDO to better apply different search strategies and enhances the search capability. Economy-driven guided learning-based strategy helps EDOs absorb valid information from dominant groups, which in turn improves the quality of the entire population. The spatial selectivity-based selection strategy achieves a balance between exploitation and exploration capabilities. To validate the performance of LMEDO, an extensive evaluation of the CEC 2018 test suite and five engineering optimization problems was performed. Parameter sensitivity analysis assisted LMEDO in determining the optimal parameter settings. Ablation experiments confirmed the effectiveness and compatibility of the improved strategies. The superiority of LMEDO is validated by comparing it with state-of-the-art algorithms such as LSHADE-SPACMA, APSM-jSO, and GLS-MPA. LMEDO received an average ranking of 2.5862 on the CEC2018 test suite and obtained a result of 1161/94/143 (+/=/−) on the Wilcoxon rank sum test. In addition, engineering design optimization problems are investigated to further demonstrate the reliability and flexibility of LMEDO. In conclusion, LMEDO is a promising variant of metaheuristic algorithms and is effective and accurate for solving complex problems.

## Introduction

Optimization problem is the process of making the objective function reach the minimum or maximum value by adjusting the values of decision variables under certain constraints. The objective function is the performance index to be optimized, the decision variables are the parameters to be adjusted, and the constraints limit the range of values of the variables or the conditions that must be met^1^. The purpose of an optimization problem is to find the optimal solution among the feasible solutions in order to achieve the optimal performance of the system or process. In the rapid development of science and technology, optimization problems become more and more complex, which puts higher demands on the solution efficiency of optimization methods. Metaheuristic algorithms, as a stochastic optimization method, are widely applied for path planning^2,3^, feature selection ^4–6^, image segmentation ^7,8^, hyperparameter optimization ^9,10^, carbon emission prediction ^11,12^ since they are not dependent on specific conditions such as convex feasible domains, continuously differentiable objective functions, or additional constraints. These metaheuristic algorithms are highly acclaimed for their ability to balance exploration and exploitation to efficiently search large and complex problem spaces ^13,14^. Due to their inherent stochastic nature, most of these methods can approximate optimal solutions in a wide variety of complex optimization scenarios.

**Fig. 1:**
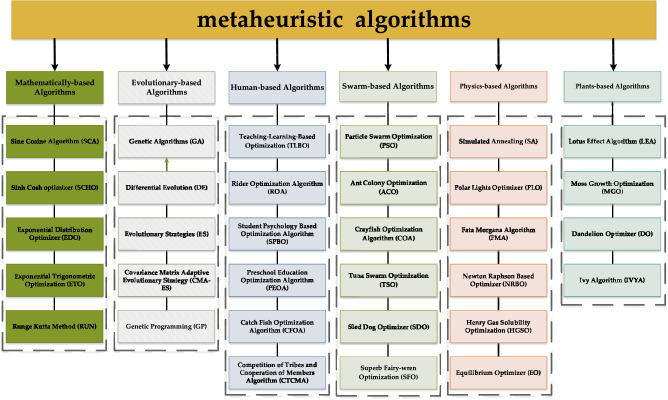
Classification of metaheuristic algorithm.

Early metaheuristic algorithms include Genetic Algorithm (GA)^15^, Particle Swarm Optimization (PSO)^16^, Differential Evolution (DE)^17^, Ant Colony Optimization (ACO)^18^ and Simulated Annealing (SA)^19^. GA and DE are designed based on the idea of evolution including selection, mutation, and crossover, and we categorize them as evolution-based algorithms. Apart from these, there are some other algorithms based on evolutionary ideas such as Evolutionary Strategies (ES)^20^, Covariance Matrix Adaptive Evolutionary Strategy (CMA-ES)^21^ and Genetic Programming (GP)^22^. SA is a physics-based algorithm which derives its idea from the simulation of solid annealing cooling process. With the development of SA, more and more physics-based and chemical law-based algorithms have been proposed, including Polar Lights Optimizer (PLO)^23^, Fata Morgana Algorithm (FMA)^24^, Newton Raphson Based Optimizer (NRBO)^25^, Henry Gas Solubility Optimization (HGSO)^26^, Equilibrium Optimizer (EO)^27^ and Special Relativity Search (SRS)^28^. PSO and ACO are swarm-based algorithms that find the optimal solution to a problem by modeling collaboration and information sharing among individuals in bird flocks and ant colonies. Other swarm-based algorithms include Crayfish Optimization Algorithm (COA)^29^, Magnificent Frigatebird Optimization (MFO)^30^, Tuna Swarm Optimization (TSO)^31^, Sled Dog Optimizer (SDO)^32^, Superb Fairy-wren Optimization (SFO)^33^, Arctic Puffin Optimization (AFO)^34^, Wolverine Optimization Algorithm (WOA)^35^, Black-winged Kite Algorithm (BKA)^36^ and Genghis Khan Shark Optimizer (GKSO)^37^. In recent years, with the continuous development of meta-heuristic algorithms, more categories of meta-heuristic algorithms have been proposed, such as mathematics-based algorithms, plant-based algorithms and human-based algorithms. Mathematics-based algorithms: relying on mathematical theories and function models, such as Sine Cosine Algorithm (SCA)^38^, Sinh Cosh optimizer (SCHO)^39^, Tangent Search Algorithm (TSA)^40^, Exponential Distribution Optimizer (EDO)^41^, Exponential Trigonometric Optimization (ETO)^42^ and Runge Kutta Method (RUN)^43^. Plant-based algorithms rely on different growth characteristics and behavioral mechanisms of plants to solve optimization problems, including Lotus Effect Algorithm (LEA)^44^, Moss Growth Optimization (MGO)^45^, Dandelion Optimizer (DO)^46^ and Ivy Algorithm (IVYA)^47^. The last category is human-based algorithms. Teaching–Learning-Based Optimization (TLBO)^48^ is the most typical example of this category and is inspired by the process of knowledge transfer between teachers and students in the educational system. Rider Optimization Algorithm (ROA)^49^, Student Psychology Based Optimization Algorithm (SPBO)^50^, Preschool Education Optimization Algorithm (PEOA)^51^, Barber Optimization Algorithm(BOA)^52^, Catch Fish Optimization Algorithm (CFOA)^53^ and Competition of Tribes and Cooperation of Members Algorithm (CTCMA)^54^ are also in this category. These five categories of metaheuristic algorithms are summarized in Fig. 1.

The Enterprise Development Optimizer (EDO) is a human-based meta-heuristic algorithm proposed by Truong in 2024^55^. The inspiration for designing the EDO algorithm comes from the business development process, which aims to find a suitable basis between exploring and utilizing solutions of optimization problems by simulating the four elements involved in business development: tasks, structures, technologies, and people. The three categories of structure, technology, and people correspond to the different search phases in the EDO algorithm: the exploration phase, the equilibrium phase, and the exploitation phase. The transition from exploration to exploitation is determined based on the switching rate. EDO confirmed its better performance in initial research, but the No Free Lunch (NFL) theorem suggests that this success may not generalize to other test problems^56^. In addition, it does have certain limitations, such as restricted local search capability and susceptibility to local optima due to diminished population diversity. Improper switching rates severely limit the performance of the EDO algorithm. The neglect of effective information of dominant populations weakens the global exploration ability of the EDO algorithm. The unorganized search direction makes it difficult to balance exploitation and exploration.

In order to improve the performance of EDO algorithms in benchmark test functions, engineering test problems, and real engineering applications, this study aims to enhance the EDO method by using labor migration theory. The time-phase based switching strategy allows EDO to better apply different search strategies and enhances the search capability. Economy-driven guided learning-based strategy helps EDOs absorb valid information from dominant groups, which in turn improves the quality of the entire population. The spatial selectivity-based selection strategy achieves a balance between exploitation and exploration capabilities. In this work, we propose the Labor Migration-based EDO algorithm called LMEDO. In order to extensively evaluate the performance of LMEDO, this paper employs the CEC2018 test suite and engineering constrained optimization problems, and conducts the following experiments. (1) Parameter sensitivity analysis: discuss the optimal parameter settings for economy-driven guided learning-based strategy. (2) Ablation experiment: discuss the effectiveness and compatibility of the three improved strategies. (3) Comparison experiments: discuss the comparison results between LMEDO and the advanced basic and improved algorithms on the CEC2018 test suite, engineering constrained optimization problems. In all experiments, we adopt Friedman test, Wilcoxon rank sum test, convergence analysis and robustness analysis to comprehensively evaluate the experimental results of LMEDO and the comparison algorithms. The main contributions of this study are shown below:

1. This study introduces and implements LMEDO, testing its performance on benchmark functions and real-world engineering problems.
2. The LMEDO is compared with other metaheuristic algorithms using the CEC2018 benchmark suite and 10 engineering problems, highlighting its competitive performance.
3. Through empirical assessments, this study showcases LMEDO’s ability to solve complex optimization problems, positioning it as a strong contender in the field.

The remaining sections of this paper are organized as follows: A concise overview of the EDO algorithm is provided in the “Enterprise development optimizer (EDO)” section. In the “Proposed labor migration-based EDO” section titled "Proposed Labor Migration-based EDO," we present three enhancement strategies along with our proposed LMEDO approach. The “Experiments and discussion” section on "Experiments and Discussion" presents the results of experiments conducted on the CEC2018 test suite and five engineering problems. Finally, a conclusion is presented.

## Enterprise development optimizer (EDO)

This section introduces relevant knowledge and principles of the EDO algorithm in preparation for the proposed improvement strategies in the next section. The EDO algorithm consists of five parts: population initialization, task, structure, technology and people. EDO first randomly generates a population in the problem space and then selects one of the task, structure, technology and people to execute through a switching mechanism. The loop is repeated until the stopping condition is satisfied and the optimal solution is output. The following presents each part in detail.

### Population initialization

In EDO, each member of the population represents a solution. Each solution consists of $D$ elements that fulfill the restrictions of the boundary conditions. These solutions then collectively form the population. Like other metaheuristic algorithms, the first step of EGO is to generate the initial population. Assuming that the search range of the problem space is $\left[ {lb,ub} \right]$, the position of the $i^{th}$ solution $X_{i}$ can be given by Eq. 1.

1 $$X_{i} = lb + rand\left( {1,D} \right) \times \left( {ub - lb} \right),i = 1,2,...,N$$

In Eq. 1, $rand\left( {1,D} \right)$ is a $D$ dimensional random vector whose elements are random numbers ranging across 0 to 1. $N$ is the number of EGO population members. After obtaining the initial population, we will evaluate the fitness of each individual, denoted as Eq. 2.

2 $$fit_{i} = F\left( {X_{i} } \right)$$

where $F\left( \cdot \right)$ denotes the objective function. $fit_{i}$ represents the fitness of the $i^{th}$ member.

### Task

Unlike the other three phases, the task phase acts only on the worst individual, i.e., when the task strategy is executed, only the worst individual is updated by Eq. 3.

3 $$X_{worst}^{{}} = lb + rand\left( {1,D} \right) \times \left( {ub - lb} \right)$$

Analysis of Eq. 3 shows that this method is mainly applied to reinitialize the worst individual $X_{worst}^{{}}$, thus enriching the population diversity.

### Structure

During the development of the enterprise, each member is influenced by other members and the best one. Thus, in the structure stage, EDO updates the members in the structure stage using Eq. 4 to represent that influence.

4 $$X_{i}^{s} \left( {t + 1} \right) = X_{i}^{s} \left( t \right) + rand\left( {1,D} \right) \times (X_{best} - X_{c}^{s} \left( t \right))$$

In Eq. 4, $X_{i}^{s} \left( t \right)$ and $X_{i}^{s} \left( {t + 1} \right)$ represent the $i^{th}$ individual’s position before and after updating in the structure phase. $X_{best}$ is the best member of the population (optimal solution). $X_{c}^{s} \left( t \right)$ denotes the center position of the other members in the structure phase that affect $X_{i}^{s} \left( t \right)$, as denoted by the Eq. 5.

5 $$X_{c}^{s} \left( t \right) = \frac{{X_{r1}^{s} \left( t \right) + X_{r2}^{s} \left( t \right) + X_{r3}^{s} \left( t \right)}}{m}$$

where $X_{r1}^{s} \left( t \right)$, $X_{r2}^{s} \left( t \right)$ and $X_{r3}^{s} \left( t \right)$ are three randomly selected individuals from the population that are different from $X_{i}^{s} \left( t \right)$. $t$ denotes the current iteration number.

### Technology

In meta-heuristic algorithms, the coordination of exploitation and exploration is crucial. EDO achieves a balance between exploitation and exploration in the technology phase by learning from the best and random members, as shown in Eq. 6.

6 $$X_{i}^{\tau } \left( {t + 1} \right) = X_{i}^{\tau } \left( t \right) + rand\left( {1,D} \right) \times \left( {X_{best} - X_{i}^{\tau } \left( t \right)} \right) + rand\left( {1,D} \right) \times \left( {X_{best} - X_{r1}^{\tau } \left( t \right)} \right)$$

In this formula, $X_{i}^{\tau } \left( t \right)$ and $X_{i}^{\tau } \left( {t + 1} \right)$ represent the $i^{th}$ individual’s position before and after updating in the technology phase. $X_{r1}^{s} \left( t \right)$ is a randomly selected member from the population that are different from $X_{i}^{\tau } \left( t \right)$.

### People

In the people phase, EDO assumes that each dimension of each member is a feature. In each iteration, EDO updates the member information by randomly selecting dimensions as Eq. 7.

7 $$X_{i,j}^{p} \left( {t + 1} \right) = X_{i,j}^{p} \left( t \right) + rand \times \left( {X_{best,j} - X_{c,j}^{p} \left( t \right)} \right)$$

In this formula, $X_{i,j}^{p} \left( t \right)$ and $X_{i,j}^{p} \left( {t + 1} \right)$ represent the $j^{th}$ dimension of $i^{th}$ individual’s position before and after updating in the people phase. $rand$ is a random number in the interval $\left[ {0,1} \right]$. $X_{c,j}^{p} \left( t \right)$ represent the $j^{th}$ dimension of center position of the other members in the people phase that affect $X_{i,j}^{p} \left( t \right)$, as denoted by Eq. 8.

8 $$X_{c}^{p} \left( t \right) = \frac{{X_{r1}^{p} \left( t \right) + X_{r2}^{p} \left( t \right) + X_{r3}^{p} \left( t \right)}}{m}$$

### Switching mechanism

The EDO determines which update method to use before each iteration after initializing the members. Considering that the task method is only used to replace the worst individual, EDO sets the frequency of its execution to 10 percent, i.e., when rand < p, EDO chooses the task method to update the population. For the remaining three methods, EDO determines which method to use by calculating the switching rate $c\left( t \right)$. When $c\left( t \right)$ is 1, 2, and 3, respectively, the corresponding methods executed by EDO are structure, technology, and people. $c\left( t \right)$ is updated as Eq. 9.

9 $$c\left( t \right) = 3 \times \left( {1 - \frac{rand \times t}{T}} \right)$$

where $T$ is the max iteration. In summary, the steps of the EDO algorithm are as follows.

Step 1. Initialization: An initial population of solutions is randomly generated.

Step 2. Evaluation: Evaluate the fitness of each solution.

Step 3. Update Population: Based on whether rand is less than 0.1 and the value of c(t), four update methods (task, structure, technology and people) are applied.

Step 4. Terminate: The process stops once the stopping condition is satisfied, usually when a set maximum number of iterations is reached.

## Proposed labor migration-based EDO

In this section, we show the specific details of the proposed motivations for each improvement strategy. Labor migration is the movement of labor from one region or country to another. Corresponding to the metaheuristic algorithm, it can be viewed as the movement of population from one search region to another. Labor migration is time-phased, economic-driven, and spatially selective. Inspired by these three properties, this work proposes three different improvement strategies: time-phase based switching strategy (TBW), economy-driven guided based learning strategy (EGL) and spatial selectivity-based selection strategy (SBS). Each improvement technique is described in detail next.

### Time-phase based switching strategy (TBW)

**Fig. 2:**
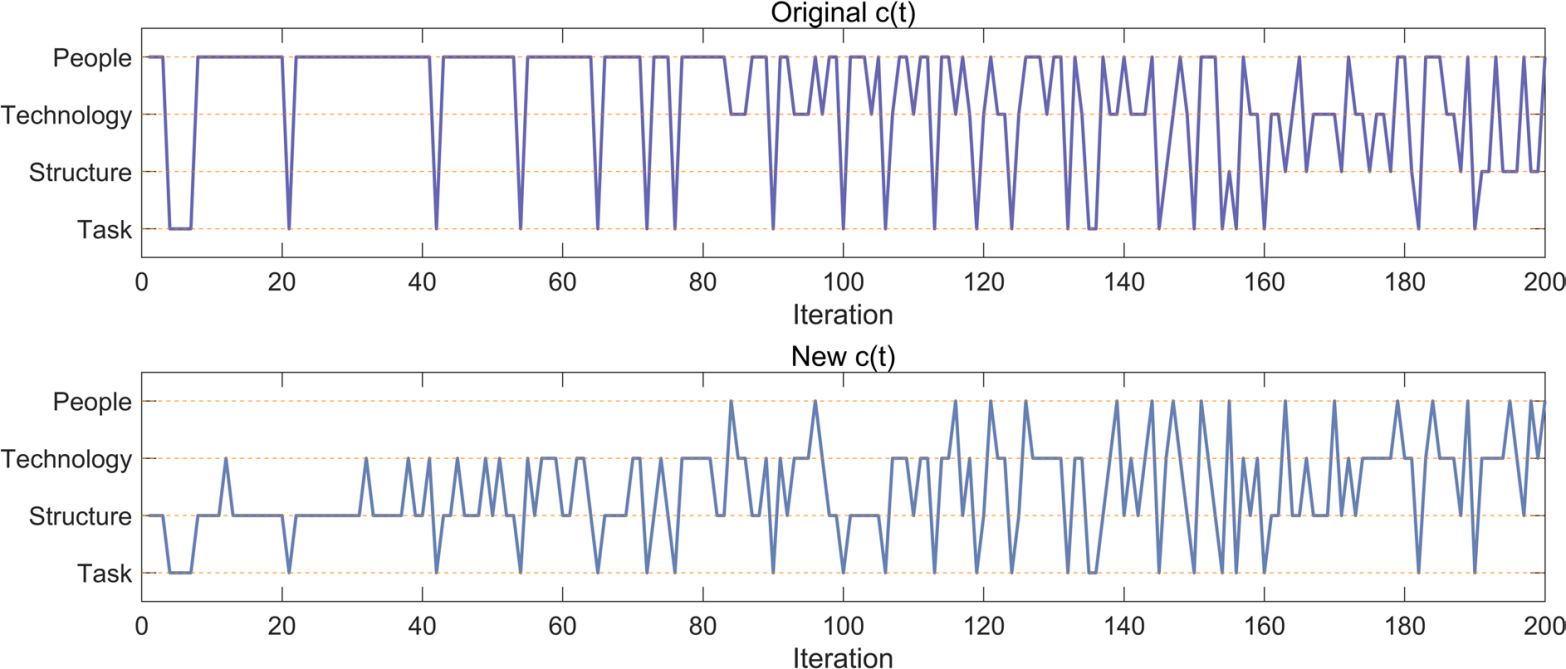
The value of original switch rate and new switch rate.

The basic EDO algorithm selects the updating strategy through a switching mechanism. Figure 2 illustrates the selection frequency of each strategy of EDO. According to Fig. 2, the basic EDO executes Task strategy and PEOPLE strategy more in the early stage. As the iterative process gets deeper, the technology strategy and structure strategy are gradually added. Task strategy is used to replace the worst individual to maintain the population diversity, so it may be executed in the whole process. People strategy is used to adjust part of the dimensions of each member, focusing on the exploitation. Structure strategy focuses more on the exploration. The existing switching mechanism of EDO algorithm is unable to fully utilize its performance, which is caused by the inappropriate switching mechanism. Inspired by the time-phase nature of labor migration, this paper proposes a time-phase based switching strategy. This strategy adjusts the search strategy according to different time phases, as shown in Fig. 2. The new switching mechanism ensures proper allocation of each search strategy and enables smooth switching between exploitation and exploration, thus improving the performance of the EDO algorithm. The time-phase based switching strategy is shown in Eq. 10.

10 $$c\left( t \right) = 3 \times rand \times \left( \frac{t}{T} \right)^{rand}$$

### Economy-driven guided based learning strategy (EGL)

All the updating methods of EDO neglect the guiding role of the dominant individuals in the population. Relying only on the guidance of the optimal member tends to cause EDO to get stuck in the local optimum. Although there are random individuals joining to expand the search area, this approach is too blind. Inspired by the economy-driven nature of labor migration, this paper proposes an economy-driven guided based learning strategy. The core of labor migration is to pursue better economic opportunities. Therefore, we simulate the labor force to adjust the migration direction according to the economic situation. The economic situation corresponds to the moving direction of the dominant group in the algorithm. The economy-driven guided based learning strategy can be represented by Eq. 11.

11 $$X_{i}^{{}} \left( {t + 1} \right) = X_{wmean} + g,g \sim G\left( {0,Cov} \right)$$

where $X_{wmean}$ is the weighted average position of the dominant group, as shown in Eq. 12. $\omega_{i}$ is the weighting factor for each individual, as shown in Eq. 13. $Cov$ is the covariance matrix of the Gaussian probability distribution model, obtained from Eq. 14.

12 $$X_{wmean} = \sum\limits_{i = 1}^{\left| S \right|} {\omega_{i} \times X_{i}^{S} \left( t \right)} ,X_{i}^{S} \left( t \right) \in S$$

13 $$\omega_{i} = \ln \left( {\left| S \right| + 1} \right)/\left( {\sum\limits_{i = 1}^{\left| S \right|} {\left( {\ln \left( {\left| S \right| + 1} \right) - \ln \left( i \right)} \right)} } \right)$$

14 $$Cov = \frac{1}{\left| S \right|} \times \sum\limits_{i = 1}^{\left| S \right|} {\left( {X_{i}^{S} \left( t \right) - \mu } \right)} \times \left( {X_{i}^{S} \left( t \right) - \mu } \right)^{T} ,X_{i}^{S} \left( t \right) \in S$$

where $X_{i}^{S} \left( t \right)$ denotes the $i^{th}$ member of the dominant group. $S$ denotes the set of dominant groups. $\left| S \right|$ is the number of dominant group members. In this paper, the individuals with the top fifty percent of fitness in each generation are stored in $S$, and the upper limit is $\left| S \right|_{\max }$. When the number of $S$ exceeds the upper limit, the redundant individuals are deleted according to the principle of first-in-first-out. For the EGL strategy, we used to update the position of those individuals whose Rank is less than 0.5. The rank of each individual can be obtained from Eq. 15.

15 $$Rank_{i} = \frac{N - i + 1}{N}$$

The quality of EDO’s population is improved through the guiding effect of the dominant population, and its global search capability is strengthened.

### Spatial selectivity based selection strategy (SBS)

Inspired by the spatial selectivity of labor migration, this paper proposes the spatial selectivity-based selection strategy. Specifically, labor migration has a clear spatial selectivity and usually flows to economically developed or resource-rich regions. In the meta-heuristic algorithm, the optimal agent has the best fitness. The suboptimal individual has the second-best fitness, but may be farther away from the optimal individual, located in a local optimum beyond the optimal point. If we consider the influence of the suboptimal individual, it may similarly cause the algorithm to fall into a local optimum. Therefore, it is necessary to consider the effects of both fitness and distance. The spatial selectivity-based selection strategy selects the individuals for guidance by calculating the combined score of each individual based on fitness and distance, as shown in the following procedure.

The Euclidean distance between each member to the optimal member is first calculated and normalized as in Eq. 16.

16 $$Dis_{i} \left( t \right) = Norm\left( {\sqrt {\left( {X_{i,1} \left( t \right) - X_{best,1} } \right)^{2} + \left( {X_{i,2} \left( t \right) - X_{best,2} } \right)^{2} + \ldots + \left( {X_{i,j} \left( t \right) - X_{best,j} } \right)^{2} } } \right)$$

The fitness of each individual is then normalized as in Eq. 17.

17 $$Fis_{i} \left( t \right) = 1 - \frac{{\left( {fit_{i} - \min \left( {fit} \right)} \right)}}{{\max \left( {fit} \right)}}$$

Finally, the weighted score for each member was calculated according to Eqs. 18 and 19.

18 $$WS_{i} = \left( {1 - \sigma } \right) \times Fis_{i} \left( t \right) + \sigma \times Dis_{i} \left( t \right)$$

19 $$\sigma_{1} = \bmod \left( {\frac{T}{10},t} \right)/T \times \left( {1 - n} \right) + n$$

Before the start of each iteration, the $WS_{i}$ was calculated for all individuals. then the highest scoring individual $X_{SBS} \left( t \right)$ was selected and introduced into Eqs. 4 and 7.

20 $$X_{i}^{s} \left( {t + 1} \right) = X_{SBS} \left( t \right) + rand\left( {1,D} \right) \times \left( {X_{best} - X_{c}^{s} \left( t \right)} \right)$$

21 $$X_{i,j}^{p} \left( {t + 1} \right) = X_{SBS,j} \left( t \right) + rand \times \left( {X_{best,j} - X_{c,j}^{p} \left( t \right)} \right)$$

Equation 4 belongs to the structure stage and is used for the exploration of the problem space. The modified Eq. 4 (i.e., Eq. 20) has improved exploration capability through the implementation of $X_{SBS} \left( t \right)$. The modified Eq. 7 (i.e., Eq. 21), through the introduction of $X_{SBS} \left( t \right)$, it has some exploration capability with strong exploitation capability. Overall, through the SBS strategy, EDO achieves a balance in the allocation of exploitation and exploration capabilities throughout the search process.

### Steps of the LMEDO algorithm

**Fig. 3:**
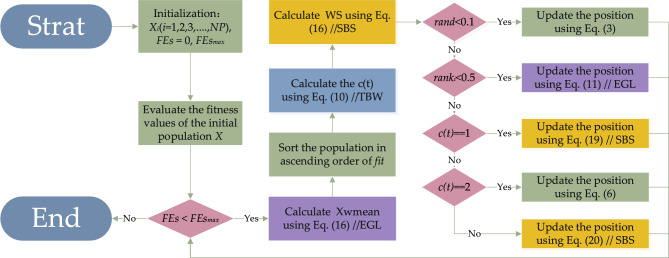
The flowchart of LMEDO.

The proposed LMEDO algorithm is obtained by integrating three improved strategies based on the basic EDO. The detailed steps are as follows and flowchart of the LMEDO is shown in Fig. 3.

Step 1. Set the parameters. the population size is $N$ , the function variable is $D$ , the upper and lower limits are $ub$ and $lb$ respectively, and maximum number of function evaluations is $FEs_{\max }$.

Step 2. An initial population of solutions is randomly generated by Eq. (1) and evaluate the fitness of each solution by Eq. (2).

Step 3. Sort the population in ascending order of *fit.*

Step 4. Calculate $X_{wmean}$ and $WS_{i}$ by Eq. (12) and Eq. (16).

Step 5. Calculate $c\left( t \right)$ by Eq. (10).

Step 6. Update Population: Based on whether rand is less than 0.1 and the value of c(t), four update methods (task, structure, technology and people) are applied.

Step 7. Terminate: The process stops once the stopping condition is satisfied, usually when a set maximum number of function evaluations is reached.

Complexity analysis is a measure of the system as a whole and evaluates the efficiency of the algorithm. We set the number of populations as $N$, the maximum number of iterations as $T$, and the number of variables as $D$. The complexity of the original algorithm EDO is $O\left( {T \times N \times D} \right)$. LMEDO does not change the original initialization process, so there is no added time complexity for this part. In the iterative process, the TBW strategy is used to replace the original switching rate, which does not involve additional fitness evaluation, so the TBW strategy does not increase the time complexity. Similarly, the EGL strategy and the original search strategy will only be executed one in each iteration, not serially, so there is no increase in time complexity. The SBS strategy is used to select high-quality individuals, which does not increase time complexity. In conclusion, the three improvement strategies proposed in this paper do not add additional computational cost for fitness and therefore, the time complexity of LMEDO is $O\left( {T \times N \times D} \right)$.

## Experiments and discussion

In this section, we evaluate the performance of the proposed LMEDO on the CEC2018 test suite and engineering optimization problems. The configuration of the experimental platform and the parameters settings of the experiments and algorithms are demonstrated in "Experimental platform and parameter settings" section. In "Descriptions of benchmark functions and performance metrics" section, the paper provides details of the CEC2018 test set and the evaluation criteria for the experiments. "Sensitivity analysis of the parameters" and "Effectiveness analysis of different improvement strategies" sections discuss the impact of the parameters and improvement strategies on LMEDO, respectively. Comprehensive comparison experiments are conducted in “Experimental case 1: CEC2018 test function analysis" and "Experimental case 2: engineering design optimization" sections for the CEC2018 test set and the engineering optimization problem, respectively.

### Experimental platform and parameter settings

The device used for the experiments in this paper, version Windows 11, 32.0 GB, processor AMD R9-7945HX CPU 2.50 GHz, and the software used is MATLAB R2023b. Meanwhile, to ensure the fairness of the experimental results, the general parameters are set as follows: population size $N = 30 \times D$, maximum number of function evaluations of the experiment $FEs_{\max } = 1000 \times D$, and each algorithm to run independently for 51 times.

The CEC 2018 benchmark functions and real-world problems are employed to evaluate the performance of LMEDO against other widely recognized metaheuristic methods, such as LSHADE, LSHADE-SPACMA, APSM-jSO, EO, IRIME, MRFO, GLS-MPA, ECO, ISGTOA, QIO, EPSCA and the original EDO. All the competitors for comparison will set their respective parameters according to the original literature, as shown in Table 1. The comparison algorithms involved in the experiment included both basic and improved algorithms. These algorithms belong to different categories of metaheuristic algorithms. The basic algorithms EO and MRFO are highly cited algorithms and are widely employed. LSHADE is the classic high-performance evolution-based algorithm. LSHADE-SPACMA and APSM-jSO are powerful DE variants. IRIME, GLS-MPA, ISGTOA and EPSCA are improved algorithms in different categories. The superior performance of LMEDO is highlighted by comparing it with these basic and improved algorithms.

**Table 1:** Parameters setting of LMEDO and other competing algorithms.

Algorithm	Parameters setting
LMEDO	$m = 3,p = 0.1,\left| S \right|_{\max } = 15D,n = 0.6$
EDO	$m = 3,p = 0.1$
LSHADE^57^	$F = 0.5,Cr = 0.5,{\text{p = 0}}{\text{.11,N}}_{\min } = 4$
LSHADE-SPACMA	$L = 0.8,P = 0.11,arc = 1.5,s = 0.5,N_{m} = 4,M = 5$
APSM-jSO^58^	$k = 3,F = 0.3,CR = 0.8,H = 6$
EO	$a_{1} = 2,a_{2} = 1,GP = 0.5$
IRIME	$L = 50,c_{1} = 0.1,c_{2} = 0.2,c_{3} = 0.9,W = 5$
MRFO^59^	$S = 2$
GLS-MPA	$F = 0.2,P = 0.5,A = 30,C_{m} = 2500$
ECO	$\beta = 1.5,H = 0.5,G = 0.2,P = 0.1$
ISGTOA	$Ar = 2N$
QIO^60^	$\alpha_{1} = 0.7,\alpha_{2} = 0.15,w = 3$
EPSCA	$a = 2,p = 90,b = 1.2,c = 0.5$

### Descriptions of benchmark functions and performance metrics

Table 2 provides an overview of CEC 2018 test functions, among which, F1–F2 are unimodal benchmark test functions with only the unique global optimal solution, which test the algorithm’s ability to search for the optimal and convergence speed. F3–F9 are multimodal benchmark test functions, with the existence of multiple local extremes, extrema that can test the algorithm’s ability to search for the global optimal and the ability to escape from the local optimal. F10–F29 are hybrid and composite multimodal benchmark test functions, which is a fixed-dimension multi-peak function to verify the balance between the algorithm’s exploration and exploitation capabilities. The test dimensions are 10-dimensional, 30-dimensional, 50-dimensional, and 100-dimensional.

**Table 2:** Detailed description of CEC2018 test functions.

Type	No	Function name	Min
Unimodal functions	F1	Shifted and rotated bent cigar function	100
	F2	Shifted and rotated Zakharov function	300
Multimodal functions	F3	Shifted and rotated Rosenbrock’s function	400
	F4	Shifted and rotated Rastrigin’s function	500
	F5	Shifted and rotated expanded Scaffer’s function	600
	F6	Shifted and rotated Lunacek Bi_Rastrigin function	700
	F7	Shifted and rotated Non-Continuous Rastrigin’s function	800
	F8	Shifted and rotated Levy function	900
	F9	Shifted and rotated Schwefel’s function	1000
Hybrid functions	F10	Hybrid function 1 (N = 3)	1100
	F11	Hybrid function 2 (N = 3)	1200
	F12	Hybrid function 3 (N = 3)	1300
	F13	Hybrid function 4 (N = 4)	1400
	F14	Hybrid function 5 (N = 4)	1500
	F15	Hybrid Function 6 (N = 4)	1600
	F16	Hybrid function 6 (N = 5)	1700
	F17	Hybrid function 6 (N = 5)	1800
	F18	Hybrid function 6 (N = 5)	1900
	F19	Hybrid function 6 (N = 6)	2000
Composition functions	F20	Composition function 1 (N = 3)	2100
	F21	Composition function 2 (N = 3)	2200
	F22	Composition function 3 (N = 4)	2300
	F23	Composition function 4 (N = 4)	2400
	F24	Composition function 5 (N = 5)	2500
	F25	Composition function 6 (N = 5)	2600
	F26	Composition function 7 (N = 6)	2700
	F27	Composition function 8 (N = 6)	2800
	F28	Composition function 9 (N = 3)	2900
	F29	Composition function 10 (N = 3)	3000
Search range: $[ - 100,100]^{D}$			

In order to evaluate the performance of the algorithms we analyze the minimum (Min), average (Avg), standard deviation (Std), obtained from the experiments. The data are analyzed using the Friedman test and Wilcoxon rank sum test. Considering coherent readability, this paper only shows statistical results and selected convergence plots, box plots, in the main content. The complete tables and figures will be available in Appendix A and Appendix B. Among them, Tables A1–A4 record the experimental results of LMEDO and comparison algorithms in 10/30/50/100 dimensions for the CEC2018 test suite. Figure B1–B4 show the convergence curves of LMEDO and comparison algorithms. Figure B5–B8 provide the entire box plots of the LMEDO and comparison algorithms.

**Fig. 4:**
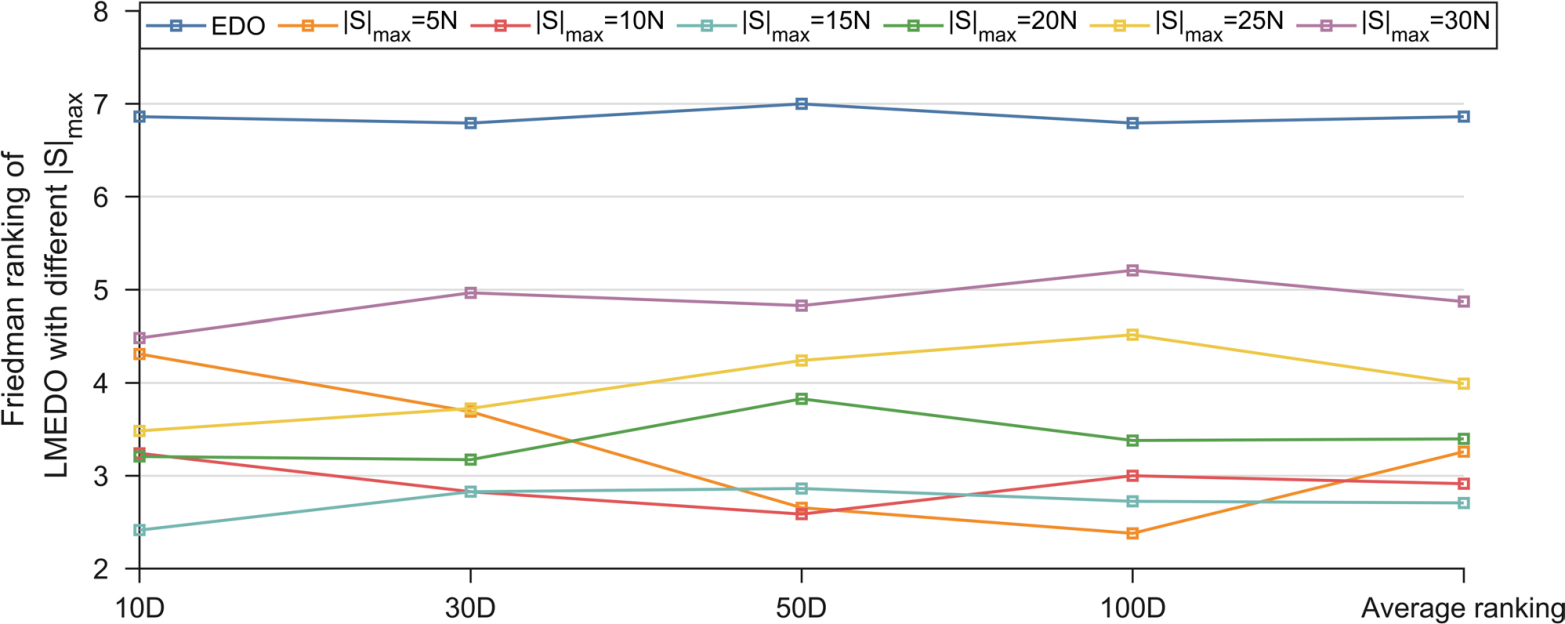
Friedman rankings of LMEDO with different $\left| S \right|_{\max }$ (α = 0.05).

**Fig. 5:**
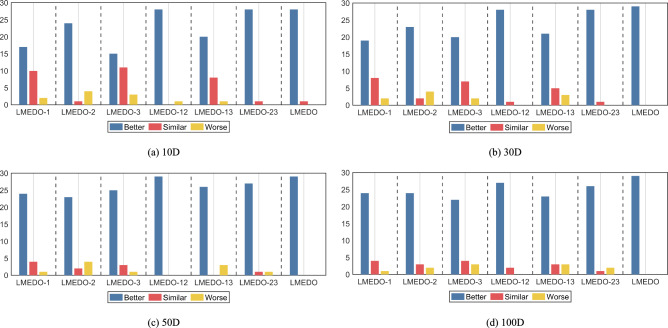
The Wilcoxon rank sum test results of LMEDO with different strategy (α = 0.05).

### Sensitivity analysis of the parameters

The performance of an algorithm is closely related to its parameter settings. In this paper, the number of dominant groups $\left| S \right|_{\max }$ of the economy-driven guided based learning strategy is critical to the performance of LMEDO. Therefore, it is necessary for us to discuss the role of $\left| S \right|_{\max }$ for LMEDO and determine the optimal parameter settings. A too small $\left| S \right|_{\max }$ is difficult to reflect the evolutionary trend of the dominant group. Too large $\left| S \right|_{\max }$ weakens the guidance of the dominant population. In this subsection, to determine the optimal $\left| S \right|_{\max }$, we use the grid search method, where $\left| S \right|_{\max }$ is taken from 5 to 30D with a value interval of 5D. Table 3 summarizes the Friedman test results for the LMEDO algorithms with different $\left| S \right|_{\max }$ on the CEC2018 test set with the best ranking is bold, and the rankings are visualized in Fig. 4.

**Table 3:** Friedman test results of LMEDO with different $\left| S \right|_{\max }$ (α = 0.05).

Dimension	EDO	|S|_max_ = 5D	|S|_max_ = 10D	|S|_max_ = 15D	|S|_max_ = 20D	|S|_max_ = 25D	|S|_max_ = 30D
10D	6.8621	4.3103	3.2414	2.4138	3.2069	3.4828	4.4828
30D	6.7931	3.6897	2.8276	2.8276	3.1724	3.7241	4.9655
50D	7.0000	2.6552	2.5862	2.8621	3.8276	4.2414	4.8276
100D	6.7931	2.3793	3.0000	2.7241	3.3793	4.5172	5.2069
Average ranking	6.8621	3.2586	2.9138	2.7069	3.3966	3.9914	4.8707

Based on Fig. 4, we can obtain two conclusions. One is that the EGL strategy significantly improves the performance of the basic EDOs, which is verified by the ranking of LMEDO with different $\left| S \right|_{\max }$ and EDO. The second is that the optimal parameter setting of $\left| S \right|_{\max }$ is $15N$, which is consistent with the previous analysis. That is, both too large $\left| S \right|_{\max }$ and too small $\left| S \right|_{\max }$ are detrimental to the performance of LMEDO. In Moreover, we can observe from Table 4 that the three LMEDO algorithms with smaller $\left| S \right|_{\max }$ bagged all the first places in the four cases. This also suggests that too large a dominant population is not favorable for guiding the population to find high-quality regions. In conclusion, the optimal value of the parameter $\left| S \right|_{\max }$ is $15N$.

**Table 4:** Descriptions of LMEDO with different strategy.

Strategy	EDO	LMEDO-1	LMEDO-2	LMEDO-3	LMEDO-12	LMEDO-13	LMEDO-23	LMEDO
TBW	No	Yes	No	No	Yes	Yes	No	Yes
EGL	No	No	Yes	No	Yes	No	Yes	Yes
SBS	No	No	No	Yes	No	Yes	Yes	Yes

### Effectiveness analysis of different improvement strategies

Before comparing with other advanced algorithms, it is necessary to discuss the impact of each improvement strategy on LMEDO. Based on the three improvement strategies, we developed six LMEDO variants as shown in Table 4. The ranking of the three LMEDO variants integrating a single improvement strategy can illustrate the magnitude of the contribution of each improvement strategy to the performance of LMEDO. The ranking of the LMEDO variants integrating two improvement strategies can reflect both whether the different strategies are compatible with each other and also indicate which strategy contributes more to LMEDO.

According to the p-values provided in Table 5, there are significant differences between LMEDO, the six LMEDO variants and the basic EDO. Specifically, LMEDO performs best when facing the test sets with different dimensions, achieving Friedman rankings of 1.2579, 1.0690, 1.4483 and 1.4138, respectively. By comparing LMEDO-1, LMEDO-2, LMEDO-3 with the basic EDO, we can conclude that all the three improvement strategies are effective in enhancing the performance of EDO, and that the contributions of the three strategies to the performance of LMEDO are, in descending order, EGL > SBS > TBW. By comparing the ranking of LMEDO-12, which integrates two improvement strategies, with that of algorithms that integrate a single improvement strategy LMEDO-1, LMEDO-2’s rankings, we can learn that TBW and EGL are able to promote each other and jointly enhance the performance of EDO. Similarly, by comparing the other results, we can learn that TBW and SBS, EGL and SBS do not negatively affect each other.

**Table 5:** Friedman test results of LMEDO with different strategy (α = 0.05).

Dimension	EDO	LMEDO-1	LMEDO-2	LMEDO-3	LMEDO-12	LMEDO-13	LMEDO-23	LMEDO	P-value
10D	7.1724	5.7586	4.6207	6.1724	2.8621	5.4483	2.6897	**1.2759**	7.62E−27
30D	7.2414	6.2069	4.9655	5.8621	2.7586	4.8966	3.0000	**1.0690**	8.91E−28
50D	7.5517	6.2069	4.6207	5.9310	2.5862	4.6552	3.0000	**1.4483**	1.34E−27
100D	7.4828	6.3793	4.4483	5.7586	2.4138	4.7586	3.3448	**1.4138**	2.36E−27
Average ranking	7.3621	6.1379	4.6638	5.9310	2.6552	4.9397	3.0086	**1.3017**	

The bold in the table represents the best value.

Figure 5 illustrates the Wilcoxon rank sum test results for the LMEDO variant and the basic EDO integrating different improvement strategies on the CEC2018 test set. The Wilcoxon rank sum test is a nonparametric statistical test used to compare the distribution of data from two sets of non-normally distributed independent samples. This method utilizes the rank of the samples in place of the sample values in data comparisons, thus effectively mitigating the effect of singular values on the sample as a whole. As a result, the method can reflect the optimization performance of the algorithm in a more scientific way than the mean and standard deviation. In this paper, the significance level p = 5% is adopted as the judgment condition for hypothesis testing. When p < 5%, it can be concluded that the difference between the two groups of samples is significant and the algorithm is statistically significant. Conversely, when p > 5%, it can be concluded that the two groups of samples are basically similar. In Fig. 5, the number of “Better/Similar/Worse” indicates the number of functions for which the LMEDO variant is better/similar/worse than the basic EDO. It is evident that LMEDO performs similarly to EDO in only one function each on 10D and 30D, and significantly outperforms EDO in the remaining functions. In summary, the improvement strategies proposed in this paper are effective in enhancing the capabilities of the EDO and these strategies are compatible with each other.

### Experimental case 1: CEC2018 test function analysis

**Fig. 6:**
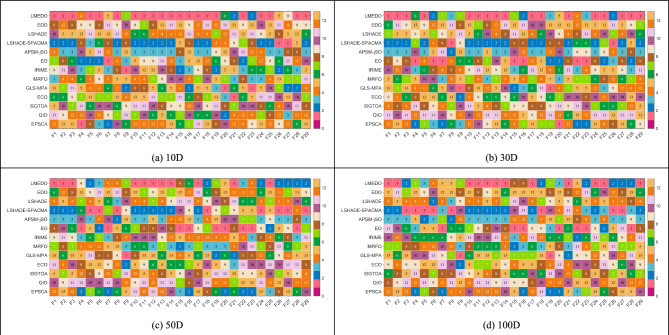
The ranking heatmaps based on “Ave” of LMEDO and comparison algorithms.

This section presents the experimental results of LMEDO and comparison algorithms on the CEC2018 test set. First, the ranking heatmaps of LMEDO and the comparison algorithms on each function are plotted based on the “Ave” of Tables A1–A4. The results obtained by LMEDO and the comparison algorithms are then statistically analyzed using the Friedman test and the Wilcoxon rank sum test. Finally, some selected convergence curves and box plots of the LMEDO and comparison algorithms solving the CEC2018 test set are shown. Figure 6 presents the rankings of LMEDO and the comparison algorithm on each function. According to Fig. 5, LMEDO obtains the highest total number of pink and orange color blocks. Therefore, we can roughly conclude that LMEDO has the best overall performance against the CEC2018 test suite.

Wilcoxon rank sum test and Friedman test were conducted based on the results obtained by the LMEDO and comparison algorithms on the CEC2018 test set. Table 6 summarizes the pairwise comparison between LMEDO and the comparison algorithm. In Table 6, the symbol “ + ” indicates that LMEDO is significantly better than the comparison algorithm. “-” indicates that LMEDO is not as good as the comparison algorithm. “ = ” shows that LMEDO and the comparison algorithm perform equally well.

**Table 6:** Wilcoxon rank sum test results of LMEDO and comparison algorithms (α = 0.05).

MSEDO vs. +/=/−	10D	30D	50D	100D	Total
EDO	28/1/0	28/1/0	29/0/0	29/0/0	114/2/0
LSHADE	27/2/0	29/0/0	26/2/1	24/1/4	106/5/5
LSHADE-SPACMA	26/1/2	26/3/0	16/4/9	18/3/8	86/11/19
APSM-jSO	27/1/1	28/0/1	27/1/1	23/4/2	105/6/5
EO	23/4/2	19/2/8	18/2/9	17/2/10	77/10/29
IRIME	19/7/3	22/3/4	19/4/6	20/4/5	80/18/18
MRFO	24/5/0	23/2/4	22/1/6	23/2/4	92/10/14
GLS-MPA	20/7/2	21/2/6	22/2/5	24/2/3	87/13/16
ECO	26/1/2	25/3/1	24/2/3	24/1/4	99/7/10
ISGTOA	27/2/0	29/0/0	29/0/0	27/0/2	114/0/2
QIO	27/1/1	29/0/0	28/1/0	27/2/0	112/4/0
EPSCA	25/4/0	22/0/7	20/0/9	19/2/8	86/6/24
Total	299/36/13	301/16/31	280/19/49	275/23/50	1161/94/143

Based on Table 6, we can draw the following findings. LMEDO receives much more “ + ” when compared with LSHADE, LSHADE-SPACMA, APSM-jSO, EO, IRIME, MRFO, GLS-MPA, ECO, IS-GTOA, QIO, EPSCA, and EDO than the sum of “−” and “ = ”. “ is much more than the sum of ” − “ and ” = ". This indicates that the overall performance of the proposed LMEDO significantly outperforms all the compared algorithms. The results of the comparison between LMEDO and EDO show that when the three improvement strategies are combined in EDO, it does not weaken its performance but significantly enhances it. The number of “ + ” obtained by LMEDO decreases as the dimensionality increases, suggesting that the performance gap of LMEDO on high-dimensional functions is not as advantageous as its performance on low-dimensional functions. In summary, LMEDO is superior (inferior) to LSHADE, LSHADE-SPACMA, APSM-jSO, EO, IRIME, MRFO, GLS-MPA, ECO, IS-GTOA, QIO, EPSCA, and EDO on 106(5), 86(19), 105(5), 77(29), 80(18), 92(14), 87(16), 99(10), 114(2), 112(0), 86(24), i.e., LMEDO is better than LSHADE, LSHADE-SPACMA, APSM-jSO, EO, IRIME, MRFO, GLS-MPA, ECO, IS-GTOA, QIO, EPSCA, and EDO in terms of overall performance.

**Fig. 7:**
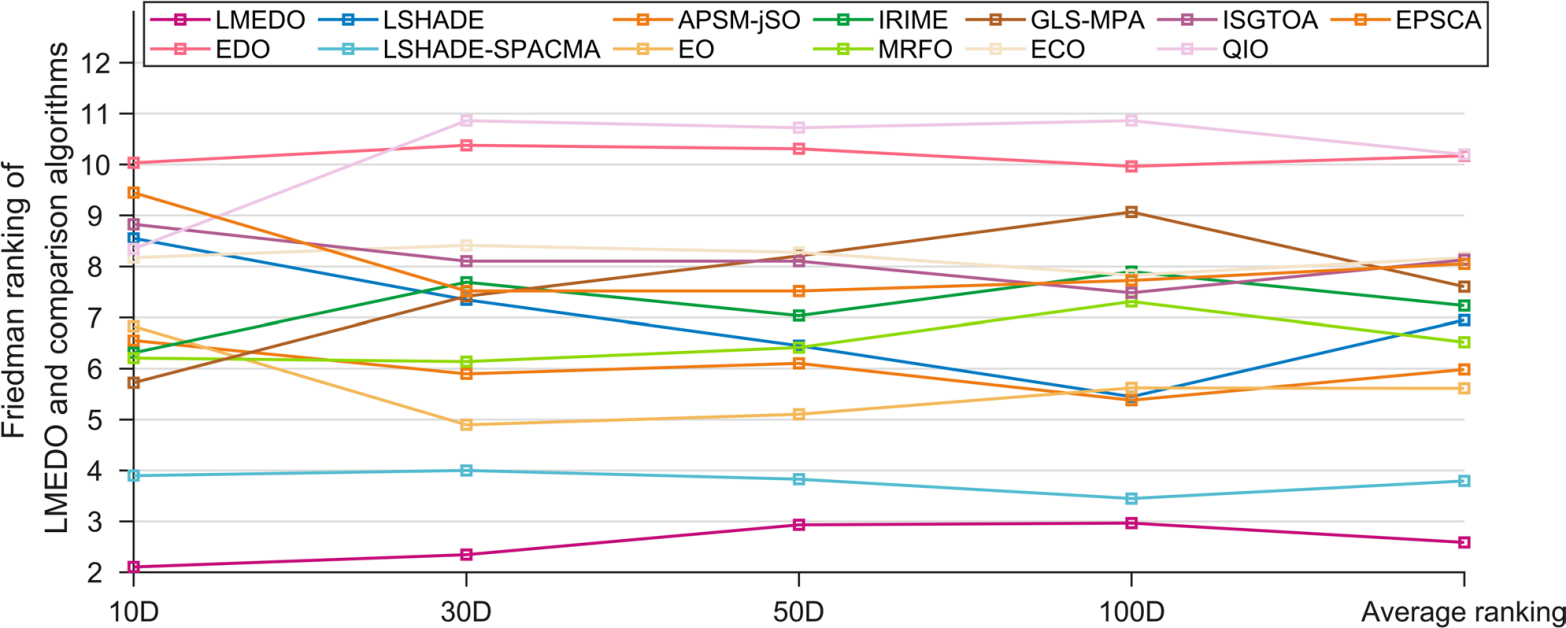
Friedman rankings of LMEDO and comparison algorithms (α = 0.05).

After completing the two-by-two comparison between LMEDO and the comparison algorithms, the Friedman test was further employed to measure the overall difference among LMEDO and LSHADE, LSHADE-SPACMA, APSM-jSO, EO, IRIME, MRFO, GLS-MPA, ECO, IS-GTOA, QIO, EPSCA, EDO. Friedman test results for LMEDO and comparison algorithms are summarized on Table 7 and a visual depiction of the Friedman rankings of each algorithm is shown in Fig. 7. From the p-values in Table 7, it is clear that there is a significant difference between LMEDO and the other algorithms. The details of Friedman’s test are given below.

- For 10D, LMEDO ranks in the first place followed by LSHADE, SPACMA, GLS-MPA, MRFO, RIME, APSM-jSO, EO, ECO, QIO, LSHADE, ISGTOA, EPSCA and EDO. This shows that LMEDO outperforms the competition algorithm when solving 10D test functions.
- For 30D, LMEDO ranks in the first place followed by LSHADE-SPACMA, EO, APSM- jSO, MRFO, LSHADE, GLS-MPA, EPSCA, IRIME, ISGTOA, ECO, EDO and QIO. This shows that LMEDO outperforms the competition algorithm when solving 50D test functions.
- For 50D, LMEDO ranks in the first place followed by LSHADE-SPACMA, EO, APSM-jSO, MRFO, LSHADE, IRIME, EPSCA, ISGTOA, GLS-MPA, ECO, EDO and QIO. This shows that MSDCS outperforms the competition algorithm when solving 50D test functions.
- For 100D, LMEDO ranks in the first place followed by LSHADE-SPACMA, APSM-jSO, LSHADE, EO, MRFO, ISGTOA, EPSCA, ECO, IRIME, GLS-MPA, EDO and QIO. This shows that LMEDO outperforms the competition algorithm when solving 100D test functions.

**Table 7:** Friedman test results of LMEDO and comparison algorithms (α = 0.05).

Algorithm	10D	30D	50D	100D	Average ranking
LMEDO	**2.1034**	**2.3448**	**2.9310**	**2.9655**	**2.5862**
EDO	10.0345	10.3793	10.3103	9.9655	10.1724
LSHADE	8.5517	7.3448	6.4483	5.4483	6.9483
LSHADE-SPACMA	3.8966	4.0000	3.8276	3.4483	3.7931
APSM- jSO	6.5517	5.8966	6.1034	5.3793	5.9828
EO	6.8276	4.8966	5.1034	5.6207	5.6121
IRIME	6.3103	7.6897	7.0345	7.8966	7.2328
MRFO	6.2069	6.1379	6.4138	7.3103	6.5172
GLS-MPA	5.7241	7.4138	8.2069	9.0690	7.6034
ECO	8.1724	8.4138	8.2759	7.8276	8.1724
ISGTOA	8.8276	8.1034	8.1034	7.4828	8.1293
QIO	8.3448	10.8621	10.7241	10.8621	10.1983
EPSCA	9.4483	7.5172	7.5172	7.7241	8.0517
P-value	3.74E−19	8.39E−22	2.72E−19	3.10E−21	

The bold in the table represents the best value.

Based on the above discussions, LMEDO is superior to LSHADE, LSHADE-SPACMA, APSM-jSO, EO, IRIME, MRFO, GLS-MPA, ECO, IS-GTOA, QIO, EPSCA, and EDO in all cases. Similarly, the results in Fig. 7 show that LMEDO performs less well on high-dimensional functions than on low-dimensional functions, which is consistent with the analytical results of the Wilcoxon rank sum test.

**Fig. 8:**
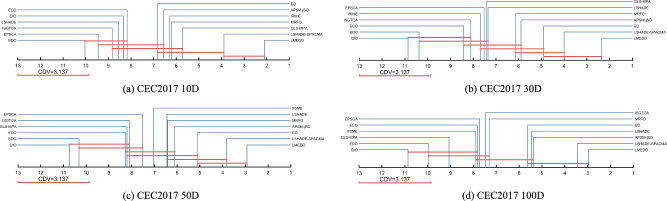
The Nemenyi test results of LMEDO and comparison algorithms.

To further determine the magnitude of differences between LMEDO and each of the comparison algorithms, The Nemenyi test was used for post hoc test. Figure 8 presents the magnitude of differences among LMEDO, LSHADE-SPACMA, APSM-jSO, LSHADE, EO, MRFO, ISGTOA, EPSCA, ECO, IRIME, GLS-MPA, EDO and QIO based on the Friedman test results. In Fig. 10, CDV is a measurement baseline. When the ranking difference between two algorithms is greater than the CDV, then there is a significant difference between these two algorithms. Conversely, there is no significant difference. That is, there is no significant difference between the algorithms connected by CDV. According to Fig. 10, LMEDO is ranked first on 10D and is only not significantly different from LSHADE-SPACMA. For 30D and 50D, LMEDO shows no significant difference in comparison with LSHADE-SPACMA and EO. For 100D, LMEDO shows no significant differences between LSHADE-SPACMA, APSM-jSO, LSHADE and EO, and is significantly superior to MRFO, ISGTOA, EPSCA, ECO, IRIME, GLS-MPA, EDO and QIO.

**Fig. 9:**
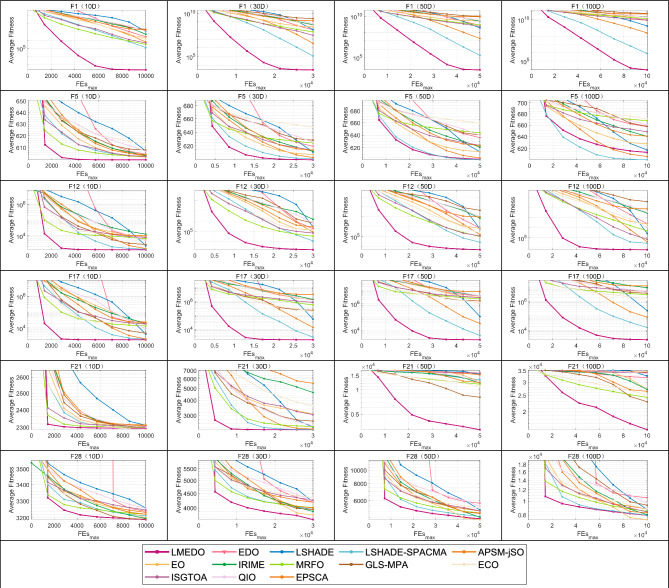
The convergence curves of LMEDO and comparison algorithms.

Since there are 29 functions in the CEC2018 test set, if all of them are shown, there are as many as 116 convergence plots. For the consistency of reading, this paper chooses some different types of functions to be shown in Fig. 9, and all the convergence curves can be obtained from Figure B1–B4 in Appendix B. A unimodal function (F1),a multimodal function (F5), two hybrid functions (F12, F17), and two composite functions (F21, F28) are chosen to be shown in Fig. 9. In addition to showing the different types of functions, the convergence curves of different dimensions are also shown at the same time, so that it is easy to observe the effect of dimensionality on the performance of the algorithm. According to Fig. 9, we can clearly observe that LMEDO is able to converge quickly in most functions and its convergence accuracy is better than that of the original EDO, which reflects the powerful search capability of LMEDO. The convergence curves for hybrid and composite functions show that LMEDO effectively avoids stagnation at local optima and premature convergence, and shows better convergence speed. By observing the LMEDO curves in different dimensions horizontally, we can learn that although the convergence accuracy of LMEDO decreases with the increase of dimensions, the trend of its convergence remains favorable. These results further confirm the effectiveness of the proposed algorithm.

**Fig. 10:**
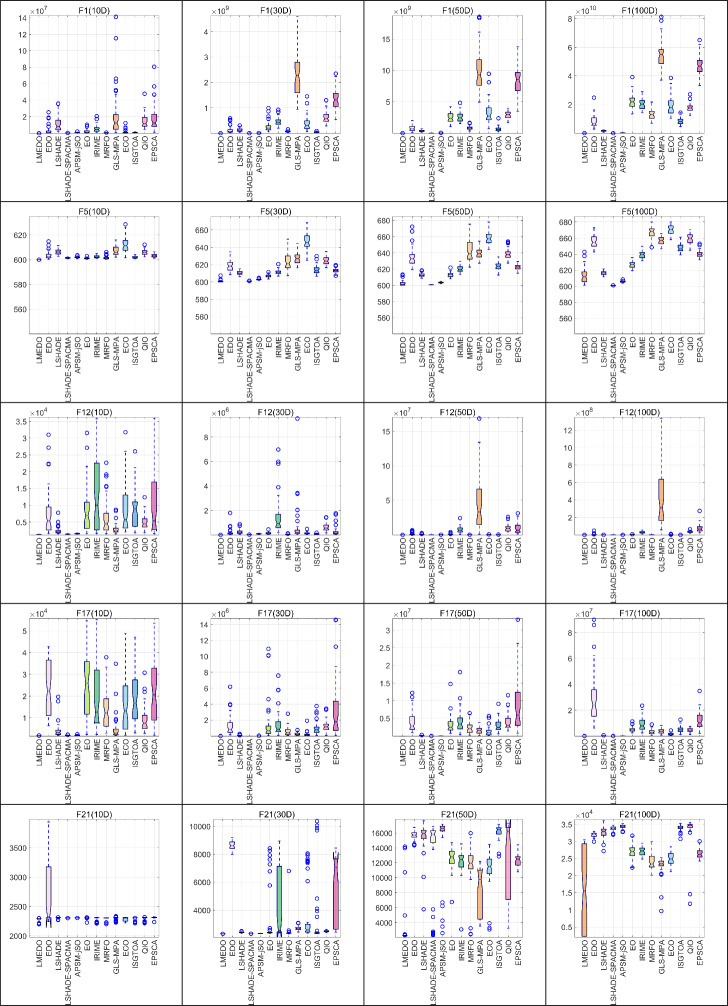

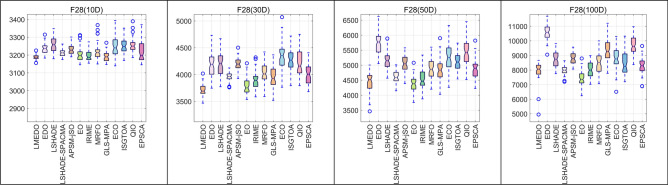
The box plots of LMEDO and comparison algorithms.

In order to examine the distributional characteristics of the solutions provided by LMEDO and the comparison algorithms, a box plot analysis as shown in Fig. 10 was performed, choosing to display the same functions as those chosen for the convergence curve analysis. In this case, the median denotes the middle value of the data set and represents the average of the sample data. The first and third quartiles denote the middle value of the minimum or maximum number and the median of the data set, respectively, and the two make up the interquartile spacing, which is the height of the box that contains 50% of the data and reflects the fluctuation of the data to a certain extent, and the flatter the box is, the more concentrated the data is. The end lines above and below the box represent the maximum and minimum values; the shorter the end lines, the more concentrated the data. If there are outliers, i.e., outliers, that are beyond the upper and lower borders, the outliers are shown with an ‘o’. As shown in Fig. 10, for most of the test function results, LMEDO has lower upper, lower, upper quartile, lower quartile, and median values than the other comparison algorithms, and exhibits greater stability and robustness overall.

### Experimental case 2: engineering design optimization

Engineering optimization problems are intimately connected to mathematical models. The key to constructing an optimized mathematical design model hinges on the design variables, target functions and constraints. Among these, restrictions assume a pivotal role in optimization problems. They not only define the range of possible solutions involved, but also exert a direct influence on the pursuit of optimal solutions. In the field of engineering design, design variables are subject to a set of constraints, such as material strength, safety standards, and cost limits. These restrictions ensure that the design is both practical and economical.

In this subsection, five authentic engineering cases (E1-E5) are selected and optimized through the application of the LMEDO. The details of these five real engineering problems are shown in the following Table 8. Table 9 shows the standard deviation, best value, mean value and ranking. The results of the Friedman test and Wilcoxon rank sum test are also summarized in Table 9. According to Table 9, the proposed LMEDO gets the first place for all the problems. Wilcoxon rank sum test shows that the performance of LMEDO is similar to LSHADE-SPACMA, MRFO, and QIO for only one engineering problem and has a significant gap with all the algorithms for all the other problems.

**Table 8:** Information about the five engineering problems.

No	Engineering application problems	D	g	h
E1	Pressure vessel design problem	4	4	0
E2	Three-bar truss design problem	2	3	0
E3	Welded beam design problem	4	5	0
E4	Gear train design Problem	4	1	1
E5	Step-cone pulley problem	5	8	3

**Table 9:** Statistical results obtained from LMEDO and comparison algorithms based on engineering problems.

No	Index	LMEDO	EDO	LSHADE	LSHADE-SPACMA	APSM-jSO	EO	IRIME	MRFO	GLS-MPA	ECO	ISGTOA	QIO	EPSCA
E1	Best	5.8701E+03	6.3603E+03	6.5608E+03	6.4296E+03	6.3968E+03	6.4912E+03	7.3587E+03	6.2695E+03	6.1609E+03	6.5578E+03	7.6135E+03	6.8865E+03	7.1798E+03
	Mean	5.8701E+03	8.1575E+03	6.7919E+03	6.5701E+03	6.5278E+03	7.1135E+03	9.2970E+03	6.6031E+03	6.8943E+03	7.3632E+03	1.0906E+04	8.8967E+03	8.0696E+03
	Std	1.1879E−03	3.0591E+03	2.5208E+02	1.1723E+02	1.5726E+02	7.0108E+02	1.8366E+03	3.7616E+02	5.4412E+02	6.6948E+02	2.8706E+03	1.5188E+03	1.4429E+03
	Rank	1	10	5	3	2	7	12	4	6	8	13	11	9
E2	Best	2.6389E+02	2.6390E+02	2.6389E+02	2.6389E+02	2.6389E+02	2.6390E+02	2.6396E+02	2.6390E+02	2.6389E+02	2.6389E+02	2.6393E+02	2.6393E+02	2.6389E+02
	Mean	2.6389E+02	2.6394E+02	2.6392E+02	2.6389E+02	2.6389E+02	2.6401E+02	2.6400E+02	2.6391E+02	2.6426E+02	2.6400E+02	2.6409E+02	2.6404E+02	2.6402E+02
	Std	2.8422E−14	5.4048E−02	4.8007E−02	0.0000E+00	1.4829E−03	1.2288E−01	4.5337E−02	1.1315E−02	6.8564E−01	1.7913E−01	2.3338E−01	1.1931E−01	1.1146E−01
	Rank	1	6	5	2	3	9	8	4	13	7	12	11	10
E3	Best	1.6928E+00	1.7467E+00	1.7325E+00	1.6928E+00	1.0890E+15	1.7428E+00	1.7431E+00	1.7439E+00	1.7206E+00	2.1851E+00	1.7669E+00	1.7475E+00	1.7354E+00
	Mean	1.6928E+00	1.8017E+00	1.7767E+00	1.6929E+00	1.0890E+15	1.7646E+00	1.8627E+00	1.8296E+00	1.7961E+00	2.3471E+00	1.8882E+00	1.8186E+00	1.9130E+00
	Std	3.8953E−07	5.4260E−02	3.1868E−02	1.2382E−04	3.6299E+07	2.1905E−02	9.9630E−02	8.3415E−02	8.6873E−02	1.4883E−01	1.1896E−01	4.8812E−02	3.0891E−01
	Rank	1	6	4	2	13	3	9	8	5	12	10	7	11
E4	Best	2.7009E−12	9.9216E−10	2.3576E−09	2.3078E−11	2.3078E−11	9.9216E−10	9.9216E−10	2.7009E−12	6.6021E−10	8.8876E−10	2.3078E−11	2.7009E−12	1.2634E−09
	Mean	7.8626E−11	6.2461E−09	1.8186E−08	5.7689E−10	7.4076E−10	9.6032E−09	5.0211E−09	1.0283E−09	1.1083E−09	1.3459E−08	1.8907E−09	8.6494E−09	2.0166E−08
	Std	7.1015E−11	7.7689E−09	2.7379E−08	6.1043E−10	1.0283E−09	1.0490E−08	6.1778E−09	1.2184E−09	7.1213E−10	1.3019E−08	1.0440E−09	1.6894E−08	1.7378E−08
	Rank	1	8	12	2	3	10	7	4	5	11	6	9	13
E5	Best	1.6086E+01	1.6636E+01	1.7330E+01	1.6115E+01	1.0098E+11	1.7054E+01	1.6331E+01	1.6877E+01	1.6268E+01	1.7449E+01	1.7579E+01	1.7153E+01	1.7711E+01
	Mean	1.6098E+01	1.6814E+01	1.7804E+01	1.6185E+01	1.0098E+11	1.7600E+01	1.8088E + 01	1.7107E + 01	1.7022E + 01	1.9710E + 01	1.7726E + 01	2.2875E + 01	2.0964E + 01
	Std	2.7093E−02	1.5137E−01	4.1424E−01	5.5683E−02	2.4805E + 04	4.2952E−01	1.2630E + 00	3.3611E−01	4.5295E−01	4.5228E + 00	2.0383E−01	8.8869E + 00	6.7025E + 00
	Rank	1	3	8	2	13	6	9	5	4	10	7	12	11
Friedman ranking		1.000	6.600	6.800	2.200	6.800	7.000	9.000	5.000	6.600	9.600	9.600	10.000	10.800
Wilcoxon rank sum test results		+ / = /−	5/0/0	5/0/0	4/1/0	4/1/0	5/0/0	5/0/0	4/1/0	5/0/0	5/0/0	5/0/0	4/1/0	5/0/0

### Discussion

Overall, LMEDO proved its excellent performance through the CEC2018 test set and engineering optimization problems. Compared to LSHADE-SPACMA, APSM-jSO, LSHADE, EO, MRFO, ISGTOA, EPSCA, ECO, IRIME, GLS-MPA, EDO and QIO, LMEDO offers better equilibrium exploration and exploitation, faster convergence and optimization accuracy. However, in the face of these advantages, there are still the following theoretical limitations and application constraints that need to be explicitly pointed out. LMEDO gains an advantage against a limited number of comparative algorithms. However, the theory of no free lunch states that there is no optimal algorithm for all optimization problems, which means that our proposed LMEDO is not the best optimizer for all optimization problems. The advantages of LMEDO are more obvious on low-dimensional problems than on high-dimensional problems. This may be due to the inability of economy-driven guided based learning strategy to compute the search trend of dominant populations when faced with high level problems. This suggests the need to further improve its ability to solve high dimensional problems. In addition, the optimal parameters of the economy-driven guided based learning strategy need to be determined experimentally, and such fixed parameters may weaken the algorithm’s searching ability, leading to premature convergence and falling into local optima in some cases. Although the LMEDO algorithm shows excellent performance in solving single-peaked and composite functions, it is significantly less efficient when dealing with hybrid and multimodal functions. Specifically, the standard deviation of the solutions for mixed and multimodal functions is much higher, indicating significant fluctuations in the solutions. This may be due to the fact that the LMEDO algorithm emphasizes the spatial search capability in the exploration phase, thus weakening its exploitation capability. Therefore, it is crucial to improve the exploitation capability of the LMEDO algorithm.

## Conclusions

This work presents a labor migration-based EDO algorithm to address the shortcomings of the original EDO algorithm. By incorporating time-phase based switching strategy, economy-driven guided based learning strategy and spatial selectivity-based selection strategy, the performance of EDO is greatly boosted. Firstly, a time-phase based switching strategy is used to ensure that the algorithm performs more global exploration in the early stage and more local exploitation in the later stage, and dynamically performs a balanced search. Secondly, an economy-driven guided based learning strategy is utilized to find the direction of population evolution, and to enhance the quality and diversity of the population through the guiding effect of dominant populations. Finally, a spatial selectivity-based selection strategy is designed to effectively realize the dynamic balance of exploration and exploitation. Optimal parameter settings were settled on the CEC2018 test set and the effectiveness and compatibility of each strategy was verified. A comprehensive comparison is performed using several advanced basic and improved algorithms and LMEDO on the CEC2018 test set and engineering optimization problems. The experimental results show that the LMEDO algorithm proposed in this paper has better search capability and excellent engineering optimization potential.

We will carry out the following future work. Conduct hybrid algorithm research to create more efficient optimization algorithms by combining them with other meta-heuristics to take advantage of multiple algorithms. Conduct algorithm development to design multi-objective versions of the LMEDO algorithm for solving more complex real-world optimization problems. Carry out a wide range of applications, using LMEDO for enterprise production scheduling, human resource allocation and inventory management.

## Supplementary Information

Supplementary Information.

## Data Availability

The data is provided within the manuscript.
