# Supplementary material for: An improved enterprise development optimizer based on labor migration for numerical optimization
Source: Sci Rep. 2025 Jul 19;15:26227. doi: 10.1038/s41598-025-07328-4 (PMC12276289; doi:10.1038/s41598-025-07328-4)
Supplement: Supplementary file 1 — Supplementary Information. [file 41598_2025_7328_MOESM1_ESM.pdf]

Appendix A and Appendix B for paper

Paper title:

An Improved Enterprise Development Optimizer Based on Labor Migration for Numerical Optimization

Author:

Dawei Zhao [1,2,+], Leidong Feng [3,+] , Yijiang Wang [4,*] Xinyu Cai [3,*] and Xiang Liu [5]

Affiliation:

1. School of Labor Economics, Capital University of Economics and Business, Beijing, China;

2. School of Economics and Management, Shandong Youth University of Political Science, Jinan 25000, China;

3. College of Business, Jiaxing University, Jiaxing 314001, China;

4. School of Labor and Human Resources, Renmin University of China, Beijing 100872, China

5. Institute For Energy Research, Jiangsu University, Zhenjiang 2120v13, China)

+These authors contributed equally to this work.

Appendix A

Table A1. Statistical results obtained from LMEDO and comparison algorithms based on CEC2018 (10D)

No.	Index	LMEDO	EDO	LSHADE	LSHADE-SPACMA	APSM-jSO	EO	IRIME	MRFO	GLS-MPA	ECO	ISGTOA	QIO	EPSCA
F1	Best	1.0000E+02	1.1802E+04	1.7040E+06	3.8232E+04	1.4739E+05	9.0512E+04	9.7638E+05	7.0535E+04	6.0874E+05	1.6112E+04	1.7667E+05	4.5790E+06	1.1798E+06
	Mean	2.0721E+02	2.7457E+06	1.1670E+07	1.2326E+05	5.7754E+05	2.1001E+06	5.4255E+06	2.9746E+05	2.1059E+07	1.0618E+06	7.2634E+05	1.4093E+07	1.7915E+07
	Std	4.3140E+02	5.0916E+06	9.5657E+06	5.3825E+04	3.4058E+05	2.0797E+06	4.1235E+06	1.5949E+05	2.7435E+07	2.0830E+06	3.7007E+05	7.9496E+06	1.5469E+07
	Rank	1	8	10	2	4	7	9	3	13	6	5	11	12
F2	Best	3.0000E+02	2.5184E+03	3.5867E+02	3.0000E+02	3.0942E+02	4.4845E+02	1.1339E+03	3.1561E+02	6.0056E+02	3.4171E+02	1.2472E+03	1.1520E+03	2.9732E+03
	Mean	3.0000E+02	8.5701E+03	1.0690E+03	3.0938E+02	3.3809E+02	3.1157E+03	4.3763E+03	4.8918E+02	1.7756E+03	1.0874E+03	4.2922E+03	4.0564E+03	1.3577E+04
	Std	3.1366E-09	5.2996E+03	6.4119E+02	1.7367E+01	2.0841E+01	1.8962E+03	2.4125E+03	2.2877E+02	9.9571E+02	6.5254E+02	1.9715E+03	2.0073E+03	8.4318E+03
	Rank	1	12	5	2	3	8	11	4	7	6	10	9	13
F3	Best	4.0000E+02	4.0134E+02	4.0559E+02	4.0355E+02	4.0405E+02	4.0416E+02	4.0109E+02	4.0122E+02	4.0086E+02	4.0037E+02	4.0402E+02	4.0474E+02	4.0557E+02
	Mean	4.0089E+02	4.1094E+02	4.0797E+02	4.0453E+02	4.0604E+02	4.0900E+02	4.1162E+02	4.0662E+02	4.1162E+02	4.1111E+02	4.0927E+02	4.1187E+02	4.1480E+02
	Std	8.7116E-01	1.5377E+01	1.2637E+00	4.3098E-01	6.6842E-01	6.3447E+00	1.4828E+01	3.0829E+00	1.3966E+01	2.6584E+01	9.5258E+00	1.0430E+01	1.6123E+01
	Rank	1	8	5	2	3	6	10	4	11	9	7	12	13
F4	Best	5.0497E+02	5.2062E+02	5.3173E+02	5.2360E+02	5.2305E+02	5.0866E+02	5.0880E+02	5.1017E+02	5.0772E+02	5.0622E+02	5.2519E+02	5.2066E+02	5.0557E+02
	Mean	5.2019E+02	5.3696E+02	5.4364E+02	5.3220E+02	5.4072E+02	5.1877E+02	5.1940E+02	5.2456E+02	5.2288E+02	5.2724E+02	5.4126E+02	5.4193E+02	5.1813E+02
	Std	5.3152E+00	8.4133E+00	7.0143E+00	4.7117E+00	6.0314E+00	5.5287E+00	6.6594E+00	1.0146E+01	7.0545E+00	1.1574E+01	7.5356E+00	7.8049E+00	6.7401E+00
	Rank	4	9	13	8	10	2	3	6	5	7	11	12	1
F5	Best	6.0000E+02	6.0049E+02	6.0271E+02	6.0085E+02	6.0087E+02	6.0045E+02	6.0107E+02	6.0047E+02	6.0209E+02	6.0162E+02	6.0071E+02	6.0230E+02	6.0130E+02
	Mean	6.0000E+02	6.0351E+02	6.0625E+02	6.0151E+02	6.0204E+02	6.0120E+02	6.0254E+02	6.0165E+02	6.0703E+02	6.1213E+02	6.0205E+02	6.0601E+02	6.0331E+02
	Std	4.3395E-05	2.8555E+00	2.1382E+00	3.4172E-01	5.7645E-01	5.0292E-01	1.1266E+00	1.2065E+00	3.6738E+00	6.3404E+00	9.2043E-01	2.2040E+00	1.3043E+00
	Rank	1	9	11	3	5	2	7	4	12	13	6	10	8
F6	Best	7.1698E+02	7.2550E+02	7.5065E+02	7.3339E+02	7.4062E+02	7.2071E+02	7.2147E+02	7.2395E+02	7.2275E+02	7.1902E+02	7.3723E+02	7.3300E+02	7.1865E+02
	Mean	7.3114E+02	7.5076E+02	7.6833E+02	7.4594E+02	7.5399E+02	7.3310E+02	7.4043E+02	7.5083E+02	7.3663E+02	7.4729E+02	7.5271E+02	7.5910E+02	7.3488E+02
	Std	5.5887E+00	1.0564E+01	8.8626E+00	5.0223E+00	6.1957E+00	5.7026E+00	8.8012E+00	1.3825E+01	7.9169E+00	1.2992E+01	7.7228E+00	9.0335E+00	7.3643E+00
	Rank	1	8	13	6	11	2	5	9	4	7	10	12	3
F7	Best	8.0534E+02	8.2346E+02	8.2160E+02	8.1236E+02	8.2336E+02	8.0600E+02	8.0732E+02	8.0780E+02	8.0893E+02	8.0831E+02	8.1531E+02	8.2160E+02	8.0700E+02
	Mean	8.2089E+02	8.3875E+02	8.4641E+02	8.3287E+02	8.4077E+02	8.1566E+02	8.2047E+02	8.2055E+02	8.2105E+02	8.2582E+02	8.3639E+02	8.3494E+02	8.1914E+02
	Std	5.7757E+00	7.1628E+00	7.8296E+00	6.3728E+00	5.7950E+00	6.0305E+00	6.8127E+00	7.1725E+00	6.1913E+00	9.1051E+00	8.2560E+00	7.4307E+00	6.9427E+00
	Rank	5	11	13	8	12	1	3	4	6	7	10	9	2
F8	Best	9.0000E+02	9.0034E+02	9.0219E+02	9.0038E+02	9.0064E+02	9.0029E+02	9.0113E+02	9.0009E+02	9.0067E+02	9.0411E+02	9.0040E+02	9.0158E+02	9.0335E+02
	Mean	9.0000E+02	9.1672E+02	9.3247E+02	9.0131E+02	9.0182E+02	9.0255E+02	9.1399E+02	9.0333E+02	9.3415E+02	1.0201E+03	9.0351E+02	9.0940E+02	9.1705E+02
	Std	1.2131E-11	2.2337E+01	2.8077E+01	6.3284E-01	9.4220E-01	3.6158E+00	1.6230E+01	5.5307E+00	2.6319E+01	1.2616E+02	3.3871E+00	7.9106E+00	1.5517E+01
	Rank	1	9	11	2	3	4	8	5	12	13	6	7	10
F9	Best	1.4550E+03	2.1003E+03	2.2173E+03	1.7569E+03	2.1815E+03	1.2708E+03	1.1837E+03	1.2862E+03	1.1390E+03	1.1936E+03	1.8774E+03	2.0331E+03	1.5490E+03

	Mean	2.0414E+03	2.4984E+03	2.6976E+03	2.2796E+03	2.7101E+03	1.8953E+03	1.6703E+03	1.9691E+03	1.6958E+03	1.9505E+03	2.3849E+03	2.6756E+03	2.0262E+03
	Std	2.0250E+02	1.8404E+02	2.3704E+02	2.1816E+02	1.7777E+02	2.8613E+02	2.3148E+02	3.6291E+02	3.1378E+02	3.1460E+02	2.6441E+02	1.9459E+02	2.8901E+02
	Rank	7	10	12	8	13	3	1	5	2	4	9	11	6
F10	Best	1.1002E+03	1.1085E+03	1.1081E+03	1.1086E+03	1.1108E+03	1.1058E+03	1.1058E+03	1.1043E+03	1.1065E+03	1.1120E+03	1.1116E+03	1.1124E+03	1.1108E+03
	Mean	1.1053E+03	1.1347E+03	1.1302E+03	1.1123E+03	1.1166E+03	1.1254E+03	1.1360E+03	1.1198E+03	1.1328E+03	1.1844E+03	1.1543E+03	1.1318E+03	1.1556E+03
	Std	1.8694E+00	4.4166E+01	1.1695E+01	2.2343E+00	3.3618E+00	1.3116E+01	3.8703E+01	8.2133E+00	2.1543E+01	7.7277E+01	2.1884E+01	7.6304E+00	6.3304E+01
	Rank	1	9	6	2	3	5	10	4	8	13	11	7	12
F11	Best	1.2153E+03	2.9078E+04	3.4799E+04	5.2866E+03	1.0744E+04	4.2688E+03	1.1021E+04	9.4365E+03	4.8812E+03	3.4296E+03	1.3573E+04	1.4228E+05	1.7767E+04
	Mean	1.4863E+03	7.4361E+05	4.4627E+05	1.6118E+04	3.2787E+04	1.1681E+06	2.0530E+06	2.7483E+05	9.4742E+05	3.6939E+05	1.7378E+06	1.4525E+06	1.8853E+06
	Std	3.3543E+02	9.2626E+05	5.6803E+05	1.0961E+04	1.6977E+04	1.6139E+06	2.5380E+06	7.5625E+05	1.8034E+06	9.0407E+05	1.9585E+06	1.2033E+06	2.6445E+06
	Rank	1	7	6	2	3	9	13	4	8	5	11	10	12
F12	Best	1.3039E+03	1.5978E+03	1.5126E+03	1.3517E+03	1.3939E+03	1.5834E+03	1.6494E+03	1.5203E+03	1.4746E+03	2.0235E+03	1.7521E+03	1.8443E+03	1.5301E+03
	Mean	1.3107E+03	7.6023E+03	2.3916E+03	1.4081E+03	1.5464E+03	8.3887E+03	1.2757E+04	6.3399E+03	2.8177E+03	9.1656E+03	8.4072E+03	5.0122E+03	1.0385E+04
	Std	4.4304E+00	6.7375E+03	1.0581E+03	4.0239E+01	8.9107E+01	6.7241E+03	1.1424E+04	5.1648E+03	1.4480E+03	7.7120E+03	5.2391E+03	2.1112E+03	9.7239E+03
	Rank	1	8	4	2	3	9	13	7	5	11	10	6	12
F13	Best	1.4047E+03	1.4798E+03	1.4303E+03	1.4245E+03	1.4266E+03	1.4523E+03	1.4448E+03	1.4361E+03	1.4323E+03	1.4317E+03	1.4741E+03	1.4341E+03	1.4600E+03
	Mean	1.4167E+03	9.0588E+03	1.4505E+03	1.4322E+03	1.4372E+03	2.6659E+03	1.5995E+03	1.5828E+03	1.4543E+03	1.4993E+03	1.5207E+03	1.4533E+03	5.0382E+03
	Std	5.0541E+00	7.9077E+03	1.1754E+01	2.9328E+00	3.8395E+00	2.1811E+03	2.8735E+02	1.8554E+02	1.2699E+01	3.4334E+01	4.6196E+01	8.6913E+00	5.5566E+03
	Rank	1	13	4	2	3	11	10	9	6	7	8	5	12
F14	Best	1.5016E+03	1.6388E+03	1.5225E+03	1.5054E+03	1.5130E+03	1.6496E+03	1.5403E+03	1.5875E+03	1.5228E+03	1.5597E+03	1.5813E+03	1.5556E+03	1.6150E+03
	Mean	1.5032E+03	5.0297E+03	1.5880E+03	1.5149E+03	1.5264E+03	5.5694E+03	2.0992E+03	2.7561E+03	1.6128E+03	1.9232E+03	2.3773E+03	1.6444E+03	9.4869E+03
	Std	9.4801E-01	1.6993E+03	3.5322E+01	6.0614E+00	7.5881E+00	4.7908E+03	6.1332E+02	1.2127E+03	5.6448E+01	5.0047E+02	5.7733E+02	8.1643E+01	8.4728E+03
	Rank	1	11	4	2	3	12	8	10	5	7	9	6	13
F15	Best	1.6011E+03	1.6273E+03	1.6143E+03	1.6239E+03	1.6557E+03	1.6060E+03	1.6035E+03	1.6053E+03	1.6092E+03	1.6029E+03	1.6251E+03	1.6359E+03	1.6034E+03
	Mean	1.6496E+03	1.7864E+03	1.7587E+03	1.6877E+03	1.7319E+03	1.6802E+03	1.6506E+03	1.7448E+03	1.6867E+03	1.7459E+03	1.7342E+03	1.7308E+03	1.7218E+03
	Std	6.9967E+01	1.0842E+02	7.6104E+01	4.2098E+01	5.6975E+01	8.6407E+01	5.5811E+01	1.0625E+02	6.7848E+01	9.5790E+01	6.4931E+01	8.1073E+01	8.8580E+01
	Rank	1	13	12	5	8	3	2	10	4	11	9	7	6
F16	Best	1.7235E+03	1.7387E+03	1.7450E+03	1.7337E+03	1.7500E+03	1.7291E+03	1.7244E+03	1.7260E+03	1.7156E+03	1.7328E+03	1.7503E+03	1.7463E+03	1.7359E+03
	Mean	1.7447E+03	1.7735E+03	1.7862E+03	1.7670E+03	1.7900E+03	1.7587E+03	1.7461E+03	1.7513E+03	1.7447E+03	1.7636E+03	1.7874E+03	1.7824E+03	1.7649E+03
	Std	8.9926E+00	2.3849E+01	2.4351E+01	1.2994E+01	2.3234E+01	1.8161E+01	1.5309E+01	1.6720E+01	1.4427E+01	1.4989E+01	2.1525E+01	2.0946E+01	2.0821E+01
	Rank	1	9	11	8	13	5	3	4	2	6	12	10	7
F17	Best	1.8009E+03	6.2925E+03	1.9188E+03	1.8591E+03	1.8442E+03	2.4069E+03	2.3104E+03	2.8923E+03	1.8668E+03	2.0323E+03	2.9907E+03	2.7967E+03	2.0994E+03
	Mean	1.8093E+03	2.2769E+04	4.0429E+03	1.9687E+03	2.0299E+03	2.4387E+04	1.8322E+04	1.3523E+04	4.6490E+03	1.6527E+04	1.8868E+04	8.9032E+03	2.1127E+04
	Std	4.5091E+00	1.2672E+04	3.2685E+03	7.9546E+01	1.1310E+02	1.3561E+04	1.3278E+04	8.8820E+03	5.4155E+03	1.3202E+04	1.0940E+04	5.5710E+03	1.3914E+04
	Rank	1	12	4	2	3	13	9	7	5	8	10	6	11
F18	Best	1.9012E+03	1.9389E+03	1.9080E+03	1.9055E+03	1.9060E+03	1.9797E+03	1.9191E+03	1.9564E+03	1.9123E+03	1.9142E+03	1.9541E+03	1.9203E+03	1.9306E+03
	Mean	1.9029E+03	5.0928E+03	1.9247E+03	1.9090E+03	1.9115E+03	1.0138E+04	2.5239E+03	3.7882E+03	1.9363E+03	2.4892E+03	3.7702E+03	2.0162E+03	1.1927E+04
	Std	7.0362E-01	4.2057E+03	1.1716E+01	1.7121E+00	3.4559E+00	9.5601E+03	8.3301E+02	2.5021E+03	3.0312E+01	2.0431E+03	2.8240E+03	1.5183E+02	9.5030E+03
	Rank	1	11	4	2	3	12	8	10	5	7	9	6	13
F19	Best	2.0016E+03	2.0550E+03	2.0410E+03	2.0358E+03	2.0512E+03	2.0255E+03	2.0224E+03	2.0131E+03	2.0165E+03	2.0322E+03	2.0532E+03	2.0476E+03	2.0270E+03
	Mean	2.0344E+03	2.1053E+03	2.0739E+03	2.0558E+03	2.0864E+03	2.0590E+03	2.0359E+03	2.0589E+03	2.0487E+03	2.1132E+03	2.1009E+03	2.1008E+03	2.0781E+03
	Std	1.5908E+01	2.7619E+01	1.6822E+01	9.6201E+00	1.7577E+01	3.6443E+01	8.1076E+00	2.4695E+01	1.8098E+01	5.0005E+01	3.8167E+01	3.1490E+01	4.8917E+01
	Rank	1	12	7	4	9	6	2	5	3	13	11	10	8
F20	Best	2.2000E+03	2.2017E+03	2.2042E+03	2.2051E+03	2.2032E+03	2.2039E+03	2.2030E+03	2.2014E+03	2.2021E+03	2.2022E+03	2.2033E+03	2.2035E+03	2.2040E+03
	Mean	2.2546E+03	2.3108E+03	2.3165E+03	2.2517E+03	2.3168E+03	2.2991E+03	2.2536E+03	2.2573E+03	2.2089E+03	2.2435E+03	2.2975E+03	2.2376E+03	2.3050E+03
	Std	6.0891E+01	5.4333E+01	5.6349E+01	4.6050E+01	4.8552E+01	4.1998E+01	5.4607E+01	5.8464E+01	1.7973E+01	5.4717E+01	6.1910E+01	5.2006E+01	3.8973E+01
	Rank	6	11	12	4	13	9	5	7	1	3	8	2	10
F21	Best	2.2000E+03	2.2457E+03	2.2700E+03	2.3034E+03	2.3053E+03	2.2273E+03	2.2106E+03	2.2006E+03	2.2284E+03	2.2222E+03	2.2175E+03	2.2162E+03	2.2364E+03
	Mean	2.2954E+03	2.6717E+03	2.3110E+03	2.3069E+03	2.3086E+03	2.3059E+03	2.3014E+03	2.2997E+03	2.3031E+03	2.2859E+03	2.3052E+03	2.3001E+03	2.3125E+03
	Std	2.1700E+01	5.6432E+02	7.8124E+00	1.1591E+00	1.0322E+00	1.1422E+01	2.7643E+01	2.6407E+01	2.3092E+01	3.1164E+01	1.8589E+01	3.0183E+01	1.1903E+01
	Rank	2	13	11	9	10	8	5	3	6	1	7	4	12
F22	Best	2.6040E+03	2.6225E+03	2.6241E+03	2.6174E+03	2.6258E+03	2.6090E+03	2.6095E+03	2.6090E+03	2.6096E+03	2.6116E+03	2.6098E+03	2.6184E+03	2.6075E+03
	Mean	2.6167E+03	2.6388E+03	2.6440E+03	2.6300E+03	2.6394E+03	2.6208E+03	2.6216E+03	2.6310E+03	2.6260E+03	2.6284E+03	2.6390E+03	2.6415E+03	2.6238E+03
	Std	7.3938E+00	1.0592E+01	9.1725E+00	4.2499E+00	5.2266E+00	6.2727E+00	7.2342E+00	1.1354E+01	8.5436E+00	1.1810E+01	1.0365E+01	9.8453E+00	8.2745E+00
	Rank	1	9	13	7	11	2	3	8	5	6	10	12	4
F23	Best	2.5000E+03	2.7596E+03	2.5285E+03	2.5693E+03	2.6484E+03	2.5173E+03	2.5114E+03	2.5019E+03	2.5020E+03	2.5076E+03	2.5431E+03	2.5241E+03	2.5769E+03
	Mean	2.6829E+03	2.7736E+03	2.7578E+03	2.7422E+03	2.7608E+03	2.7364E+03	2.7309E+03	2.7110E+03	2.5652E+03	2.7225E+03	2.7519E+03	2.7186E+03	2.7441E+03
	Std	1.0446E+02	7.9772E+00	4.9453E+01	4.4068E+01	2.4606E+01	4.3079E+01	6.7683E+01	8.9174E+01	7.9852E+01	7.6126E+01	5.4700E+01	9.1344E+01	3.3429E+01
	Rank	2	13	11	8	12	7	6	3	1	5	10	4	9
F24	Best	2.8977E+03	2.8980E+03	2.9011E+03	2.8987E+03	2.8992E+03	2.8995E+03	2.9000E+03	2.8980E+03	2.8994E+03	2.8987E+03	2.9002E+03	2.9014E+03	2.9023E+03
	Mean	2.9137E+03	2.9357E+03	2.9356E+03	2.9225E+03	2.9267E+03	2.9404E+03	2.9312E+03	2.9262E+03	2.9311E+03	2.9403E+03	2.9354E+03	2.9354E+03	2.9499E+03
	Std	2.1867E+01	9.7555E+01	1.9631E+01	2.2812E+01	2.2414E+01	1.5085E+01	2.2021E+01	2.7718E+01	2.0325E+01	2.9777E+01	1.8747E+01	1.8847E+01	1.5047E+01
	Rank	1	10	9	2	4	12	6	3	5	11	8	7	13
F25	Best	2.8000E+03	2.8312E+03	2.9097E+03	2.9005E+03	2.9018E+03	2.8321E+03	2.6346E+03	2.8131E+03	2.6314E+03	2.6838E+03	2.8345E+03	2.8850E+03	2.9329E+03
	Mean	2.8907E+03	3.7399E+03	2.9444E+03	2.9015E+03	2.9052E+03	2.9593E+03	2.9489E+03	2.9295E+03	2.9776E+03	2.9917E+03	2.9451E+03	2.9561E+03	3.0150E+03
	Std	1.6190E+02	5.8499E+02	1.9698E+01	4.3452E-01	6.2707E+00	3.8847E+01	5.4520E+01	1.3519E+02	5.5972E+01	9.0282E+01	4.2578E+01	3.6330E+01	6.1682E+01
	Rank	1	13	5	2	3	9	7	4	10	11	6	8	12
F26	Best	3.0889E+03	3.0927E+03	3.0914E+03	3.0892E+03	3.0901E+03	3.0904E+03	3.0898E+03	3.0951E+03	3.0898E+03	3.0901E+03	3.0907E+03	3.0996E+03	3.0907E+03
	Mean	3.0943E+03	3.0992E+03	3.0954E+03	3.0916E+03	3.0934E+03	3.0945E+03	3.0932E+03	3.1107E+03	3.0952E+03	3.0983E+03	3.0939E+03	3.1107E+03	3.0963E+03
	Std	1.9918E+00	2.5350E+00	2.4256E+00	1.2118E+00	2.1711E+00	2.4691E+00	2.2282E+00	2.4243E+01	2.7830E+00	1.5821E+01	1.8997E+00	8.1037E+00	3.7702E+00
	Rank	5	11	8	1	3	6	2	13	7	10	4	12	9
F27	Best	3.1000E+03	3.1050E+03	3.1437E+03	3.1033E+03	3.1049E+03	3.1245E+03	3.1091E+03	2.8253E+03	3.1028E+03	3.1454E+03	3.1746E+03	2.9507E+03	3.1854E+03
	Mean	3.2771E+03	3.2089E+03	3.2390E+03	3.1749E+03	3.2609E+03	3.3027E+03	3.2409E+03	3.2484E+03	3.1990E+03	3.3644E+03	3.2818E+03	3.1999E+03	3.3357E+03
	Std	1.4871E+02	8.8676E+01	8.8562E+01	1.0807E+02	1.1978E+02	1.0905E+02	8.3148E+01	1.4816E+02	4.1595E+01	1.0923E+02	1.1649E+02	8.2571E+01	9.7892E+01
	Rank	9	4	5	1	8	11	6	7	2	13	10	3	12
F28	Best	3.1553E+03	3.1829E+03	3.1792E+03	3.1744E+03	3.1900E+03	3.1453E+03	3.1543E+03	3.1499E+03	3.1466E+03	3.1391E+03	3.1693E+03	3.1858E+03	3.1453E+03
	Mean	3.1894E+03	3.2375E+03	3.2574E+03	3.2108E+03	3.2310E+03	3.1995E+03	3.1953E+03	3.2206E+03	3.1915E+03	3.2452E+03	3.2519E+03	3.2563E+03	3.2206E+03
	Std	1.3754E+01	3.0811E+01	4.2061E+01	1.8685E+01	2.5911E+01	3.8912E+01	2.9087E+01	4.4160E+01	2.8640E+01	5.8961E+01	3.9682E+01	3.8288E+01	6.2874E+01
	Rank	1	9	13	5	8	4	3	7	2	10	11	12	6
F29	Best	3.4049E+03	4.8707E+04	5.4127E+03	3.6705E+03	4.4470E+03	7.1718E+03	3.7800E+03	1.4484E+04	4.0368E+03	4.9301E+03	5.1365E+04	9.8253E+03	1.1253E+04
	Mean	3.6818E+03	8.2578E+05	3.6777E+05	3.9554E+04	1.8435E+05	8.6251E+05	2.6964E+05	8.8983E+05	2.2771E+05	4.0217E+05	7.1647E+05	1.1275E+06	1.2089E+06
	Std	8.2963E+02	1.2615E+06	6.5289E+05	1.6706E+05	3.9090E+05	7.3306E+05	3.2687E+05	1.0793E+06	3.8668E+05	4.2571E+05	3.8801E+05	1.4272E+06	9.2854E+05
	Rank	1	9	6	2	3	10	5	11	4	7	8	12	13

Table A2. Statistical results obtained from LMEDO and comparison algorithms based on CEC2018 (30D)

No.	Index	LMEDO	EDO	LSHADE	LSHADE-SPACMA	APSM-Jso	EO	IRIME	MRFO	GLS-MPA	ECO	ISGTOA	QIO	EPSCA
F1	Best	1.0000E+02	1.1802E+04	1.7040E+06	3.8232E+04	1.4739E+05	9.0512E+04	9.7638E+05	7.0535E+04	6.0874E+05	1.6112E+04	1.7667E+05	4.5790E+06	1.1798E+06
	Mean	2.0721E+02	2.7457E+06	1.1670E+07	1.2326E+05	5.7754E+05	2.1001E+06	5.4255E+06	2.9746E+05	2.1059E+07	1.0618E+06	7.2634E+05	1.4093E+07	1.7915E+07
	Std	4.3140E+02	5.0916E+06	9.5657E+06	5.3825E+04	3.4058E+05	2.0797E+06	4.1235E+06	1.5949E+05	2.7435E+07	2.0830E+06	3.7007E+05	7.9496E+06	1.5469E+07
	Rank	1	8	10	2	4	7	9	3	13	6	5	11	12
F2	Best	3.0000E+02	2.5184E+03	3.5867E+02	3.0000E+02	3.0942E+02	4.4845E+02	1.1339E+03	3.1561E+02	6.0056E+02	3.4171E+02	1.2472E+03	1.1520E+03	2.9732E+03
	Mean	3.0000E+02	8.5701E+03	1.0690E+03	3.0938E+02	3.3809E+02	3.1157E+03	4.3763E+03	4.8918E+02	1.7756E+03	1.0874E+03	4.2922E+03	4.0564E+03	1.3577E+04
	Std	3.1366E-09	5.2996E+03	6.4119E+02	1.7367E+01	2.0841E+01	1.8962E+03	2.4125E+03	2.2877E+02	9.9571E+02	6.5254E+02	1.9715E+03	2.0073E+03	8.4318E+03
	Rank	1	12	5	2	3	8	11	4	7	6	10	9	13
F3	Best	4.0000E+02	4.0134E+02	4.0559E+02	4.0355E+02	4.0405E+02	4.0416E+02	4.0109E+02	4.0122E+02	4.0086E+02	4.0037E+02	4.0402E+02	4.0474E+02	4.0557E+02
	Mean	4.0089E+02	4.1094E+02	4.0797E+02	4.0453E+02	4.0604E+02	4.0900E+02	4.1162E+02	4.0662E+02	4.1162E+02	4.1111E+02	4.0927E+02	4.1187E+02	4.1480E+02
	Std	8.7116E-01	1.5377E+01	1.2637E+00	4.3098E-01	6.6842E-01	6.3447E+00	1.4828E+01	3.0829E+00	1.3966E+01	2.6584E+01	9.5258E+00	1.0430E+01	1.6123E+01
	Rank	1	8	5	2	3	6	10	4	11	9	7	12	13
F4	Best	5.0497E+02	5.2062E+02	5.3173E+02	5.2360E+02	5.2305E+02	5.0866E+02	5.0880E+02	5.1017E+02	5.0772E+02	5.0622E+02	5.2519E+02	5.2066E+02	5.0557E+02
	Mean	5.2019E+02	5.3696E+02	5.4364E+02	5.3220E+02	5.4072E+02	5.1877E+02	5.1940E+02	5.2456E+02	5.2288E+02	5.2724E+02	5.4126E+02	5.4193E+02	5.1813E+02
	Std	5.3152E+00	8.4133E+00	7.0143E+00	4.7117E+00	6.0314E+00	5.5287E+00	6.6594E+00	1.0146E+01	7.0545E+00	1.1574E+01	7.5356E+00	7.8049E+00	6.7401E+00
	Rank	4	9	13	8	10	2	3	6	5	7	11	12	1
F5	Best	6.0000E+02	6.0049E+02	6.0271E+02	6.0085E+02	6.0087E+02	6.0045E+02	6.0107E+02	6.0047E+02	6.0209E+02	6.0162E+02	6.0071E+02	6.0230E+02	6.0130E+02
	Mean	6.0000E+02	6.0351E+02	6.0625E+02	6.0151E+02	6.0204E+02	6.0120E+02	6.0254E+02	6.0165E+02	6.0703E+02	6.1213E+02	6.0205E+02	6.0601E+02	6.0331E+02
	Std	4.3395E-05	2.8555E+00	2.1382E+00	3.4172E-01	5.7645E-01	5.0292E-01	1.1266E+00	1.2065E+00	3.6738E+00	6.3404E+00	9.2043E-01	2.2040E+00	1.3043E+00
	Rank	1	9	11	3	5	2	7	4	12	13	6	10	8
F6	Best	7.1698E+02	7.2550E+02	7.5065E+02	7.3339E+02	7.4062E+02	7.2071E+02	7.2147E+02	7.2395E+02	7.2275E+02	7.1902E+02	7.3723E+02	7.3300E+02	7.1865E+02
	Mean	7.3114E+02	7.5076E+02	7.6833E+02	7.4594E+02	7.5399E+02	7.3310E+02	7.4043E+02	7.5083E+02	7.3663E+02	7.4729E+02	7.5271E+02	7.5910E+02	7.3488E+02
	Std	5.5887E+00	1.0564E+01	8.8626E+00	5.0223E+00	6.1957E+00	5.7026E+00	8.8012E+00	1.3825E+01	7.9169E+00	1.2992E+01	7.7228E+00	9.0335E+00	7.3643E+00
	Rank	1	8	13	6	11	2	5	9	4	7	10	12	3
F7	Best	8.0534E+02	8.2346E+02	8.2160E+02	8.1236E+02	8.2336E+02	8.0600E+02	8.0732E+02	8.0780E+02	8.0893E+02	8.0831E+02	8.1531E+02	8.2160E+02	8.0700E+02
	Mean	8.2089E+02	8.3875E+02	8.4641E+02	8.3287E+02	8.4077E+02	8.1566E+02	8.2047E+02	8.2055E+02	8.2105E+02	8.2582E+02	8.3639E+02	8.3494E+02	8.1914E+02
	Std	5.7757E+00	7.1628E+00	7.8296E+00	6.3728E+00	5.7950E+00	6.0305E+00	6.8127E+00	7.1725E+00	6.1913E+00	9.1051E+00	8.2560E+00	7.4307E+00	6.9427E+00
	Rank	5	11	13	8	12	1	3	4	6	7	10	9	2
F8	Best	9.0000E+02	9.0034E+02	9.0219E+02	9.0038E+02	9.0064E+02	9.0029E+02	9.0113E+02	9.0009E+02	9.0067E+02	9.0411E+02	9.0040E+02	9.0158E+02	9.0335E+02
	Mean	9.0000E+02	9.1672E+02	9.3247E+02	9.0131E+02	9.0182E+02	9.0255E+02	9.1399E+02	9.0333E+02	9.3415E+02	1.0201E+03	9.0351E+02	9.0940E+02	9.1705E+02
	Std	1.2131E-11	2.2337E+01	2.8077E+01	6.3284E-01	9.4220E-01	3.6158E+00	1.6230E+01	5.5307E+00	2.6319E+01	1.2616E+02	3.3871E+00	7.9106E+00	1.5517E+01
	Rank	1	9	11	2	3	4	8	5	12	13	6	7	10
F9	Best	1.4550E+03	2.1003E+03	2.2173E+03	1.7569E+03	2.1815E+03	1.2708E+03	1.1837E+03	1.2862E+03	1.1390E+03	1.1936E+03	1.8774E+03	2.0331E+03	1.5490E+03
	Mean	2.0414E+03	2.4984E+03	2.6976E+03	2.2796E+03	2.7101E+03	1.8953E+03	1.6703E+03	1.9691E+03	1.6958E+03	1.9505E+03	2.3849E+03	2.6756E+03	2.0262E+03
	Std	2.0250E+02	1.8404E+02	2.3704E+02	2.1816E+02	1.7777E+02	2.8613E+02	2.3148E+02	3.6291E+02	3.1378E+02	3.1460E+02	2.6441E+02	1.9459E+02	2.8901E+02
	Rank	7	10	12	8	13	3	1	5	2	4	9	11	6
F10	Best	1.1002E+03	1.1085E+03	1.1081E+03	1.1086E+03	1.1108E+03	1.1058E+03	1.1058E+03	1.1043E+03	1.1065E+03	1.1120E+03	1.1116E+03	1.1124E+03	1.1108E+03
	Mean	1.1053E+03	1.1347E+03	1.1302E+03	1.1123E+03	1.1166E+03	1.1254E+03	1.1360E+03	1.1198E+03	1.1328E+03	1.1844E+03	1.1543E+03	1.1318E+03	1.1556E+03
	Std	1.8694E+00	4.4166E+01	1.1695E+01	2.2343E+00	3.3618E+00	1.3116E+01	3.8703E+01	8.2133E+00	2.1543E+01	7.7277E+01	2.1884E+01	7.6304E+00	6.3304E+01
	Rank	1	9	6	2	3	5	10	4	8	13	11	7	12
F11	Best	1.2153E+03	2.9078E+04	3.4799E+04	5.2866E+03	1.0744E+04	4.2688E+03	1.1021E+04	9.4365E+03	4.8812E+03	3.4296E+03	1.3573E+04	1.4228E+05	1.7767E+04
	Mean	1.4863E+03	7.4361E+05	4.4627E+05	1.6118E+04	3.2787E+04	1.1681E+06	2.0530E+06	2.7483E+05	9.4742E+05	3.6939E+05	1.7378E+06	1.4525E+06	1.8853E+06
	Std	3.3543E+02	9.2626E+05	5.6803E+05	1.0961E+04	1.6977E+04	1.6139E+06	2.5380E+06	7.5625E+05	1.8034E+06	9.0407E+05	1.9585E+06	1.2033E+06	2.6445E+06
	Rank	1	7	6	2	3	9	13	4	8	5	11	10	12

F12	Best	1.3039E+03	1.5978E+03	1.5126E+03	1.3517E+03	1.3939E+03	1.5834E+03	1.6494E+03	1.5203E+03	1.4746E+03	2.0235E+03	1.7521E+03	1.8443E+03	1.5301E+03
	Mean	1.3107E+03	7.6023E+03	2.3916E+03	1.4081E+03	1.5464E+03	8.3887E+03	1.2757E+04	6.3399E+03	2.8177E+03	9.1656E+03	8.4072E+03	5.0122E+03	1.0385E+04
	Std	4.4304E+00	6.7375E+03	1.0581E+03	4.0239E+01	8.9107E+01	6.7241E+03	1.1424E+04	5.1648E+03	1.4480E+03	7.7120E+03	5.2391E+03	2.1112E+03	9.7239E+03
	Rank	1	8	4	2	3	9	13	7	5	11	10	6	12
F13	Best	1.4047E+03	1.4798E+03	1.4303E+03	1.4245E+03	1.4266E+03	1.4523E+03	1.4448E+03	1.4361E+03	1.4323E+03	1.4317E+03	1.4741E+03	1.4341E+03	1.4600E+03
	Mean	1.4167E+03	9.0588E+03	1.4505E+03	1.4322E+03	1.4372E+03	2.6659E+03	1.5995E+03	1.5828E+03	1.4543E+03	1.4993E+03	1.5207E+03	1.4533E+03	5.0382E+03
	Std	5.0541E+00	7.9077E+03	1.1754E+01	2.9328E+00	3.8395E+00	2.1811E+03	2.8735E+02	1.8554E+02	1.2699E+01	3.4334E+01	4.6196E+01	8.6913E+00	5.5566E+03
	Rank	1	13	4	2	3	11	10	9	6	7	8	5	12
F14	Best	1.5016E+03	1.6388E+03	1.5225E+03	1.5054E+03	1.5130E+03	1.6496E+03	1.5403E+03	1.5875E+03	1.5228E+03	1.5597E+03	1.5813E+03	1.5556E+03	1.6150E+03
	Mean	1.5032E+03	5.0297E+03	1.5880E+03	1.5149E+03	1.5264E+03	5.5694E+03	2.0992E+03	2.7561E+03	1.6128E+03	1.9232E+03	2.3773E+03	1.6444E+03	9.4869E+03
	Std	9.4801E-01	1.6993E+03	3.5322E+01	6.0614E+00	7.5881E+00	4.7908E+03	6.1332E+02	1.2127E+03	5.6448E+01	5.0047E+02	5.7733E+02	8.1643E+01	8.4728E+03
	Rank	1	11	4	2	3	12	8	10	5	7	9	6	13
F15	Best	1.6011E+03	1.6273E+03	1.6143E+03	1.6239E+03	1.6557E+03	1.6060E+03	1.6035E+03	1.6053E+03	1.6092E+03	1.6029E+03	1.6251E+03	1.6359E+03	1.6034E+03
	Mean	1.6496E+03	1.7864E+03	1.7587E+03	1.6877E+03	1.7319E+03	1.6802E+03	1.6506E+03	1.7448E+03	1.6867E+03	1.7459E+03	1.7342E+03	1.7308E+03	1.7218E+03
	Std	6.9967E+01	1.0842E+02	7.6104E+01	4.2098E+01	5.6975E+01	8.6407E+01	5.5811E+01	1.0625E+02	6.7848E+01	9.5790E+01	6.4931E+01	8.1073E+01	8.8580E+01
	Rank	1	13	12	5	8	3	2	10	4	11	9	7	6
F16	Best	1.7235E+03	1.7387E+03	1.7450E+03	1.7337E+03	1.7500E+03	1.7291E+03	1.7244E+03	1.7260E+03	1.7156E+03	1.7328E+03	1.7503E+03	1.7463E+03	1.7359E+03
	Mean	1.7447E+03	1.7735E+03	1.7862E+03	1.7670E+03	1.7900E+03	1.7587E+03	1.7461E+03	1.7513E+03	1.7447E+03	1.7636E+03	1.7874E+03	1.7824E+03	1.7649E+03
	Std	8.9926E+00	2.3849E+01	2.4351E+01	1.2994E+01	2.3234E+01	1.8161E+01	1.5309E+01	1.6720E+01	1.4427E+01	1.4989E+01	2.1525E+01	2.0946E+01	2.0821E+01
	Rank	1	9	11	8	13	5	3	4	2	6	12	10	7
F17	Best	1.8009E+03	6.2925E+03	1.9188E+03	1.8591E+03	1.8442E+03	2.4069E+03	2.3104E+03	2.8923E+03	1.8668E+03	2.0323E+03	2.9907E+03	2.7967E+03	2.0994E+03
	Mean	1.8093E+03	2.2769E+04	4.0429E+03	1.9687E+03	2.0299E+03	2.4387E+04	1.8322E+04	1.3523E+04	4.6490E+03	1.6527E+04	1.8868E+04	8.9032E+03	2.1127E+04
	Std	4.5091E+00	1.2672E+04	3.2685E+03	7.9546E+01	1.1310E+02	1.3561E+04	1.3278E+04	8.8820E+03	5.4155E+03	1.3202E+04	1.0940E+04	5.5710E+03	1.3914E+04
	Rank	1	12	4	2	3	13	9	7	5	8	10	6	11
F18	Best	1.9012E+03	1.9389E+03	1.9080E+03	1.9055E+03	1.9060E+03	1.9797E+03	1.9191E+03	1.9564E+03	1.9123E+03	1.9142E+03	1.9541E+03	1.9203E+03	1.9306E+03
	Mean	1.9029E+03	5.0928E+03	1.9247E+03	1.9090E+03	1.9115E+03	1.0138E+04	2.5239E+03	3.7882E+03	1.9363E+03	2.4892E+03	3.7702E+03	2.0162E+03	1.1927E+04
	Std	7.0362E-01	4.2057E+03	1.1716E+01	1.7121E+00	3.4559E+00	9.5601E+03	8.3301E+02	2.5021E+03	3.0312E+01	2.0431E+03	2.8240E+03	1.5183E+02	9.5030E+03
	Rank	1	11	4	2	3	12	8	10	5	7	9	6	13
F19	Best	2.0016E+03	2.0550E+03	2.0410E+03	2.0358E+03	2.0512E+03	2.0255E+03	2.0224E+03	2.0131E+03	2.0165E+03	2.0322E+03	2.0532E+03	2.0476E+03	2.0270E+03
	Mean	2.0344E+03	2.1053E+03	2.0739E+03	2.0558E+03	2.0864E+03	2.0590E+03	2.0359E+03	2.0589E+03	2.0487E+03	2.1132E+03	2.1009E+03	2.1008E+03	2.0781E+03
	Std	1.5908E+01	2.7619E+01	1.6822E+01	9.6201E+00	1.7577E+01	3.6443E+01	8.1076E+00	2.4695E+01	1.8098E+01	5.0005E+01	3.8167E+01	3.1490E+01	4.8917E+01
	Rank	1	12	7	4	9	6	2	5	3	13	11	10	8
F20	Best	2.2000E+03	2.2017E+03	2.2042E+03	2.2051E+03	2.2032E+03	2.2039E+03	2.2030E+03	2.2014E+03	2.2021E+03	2.2022E+03	2.2033E+03	2.2035E+03	2.2040E+03
	Mean	2.2546E+03	2.3108E+03	2.3165E+03	2.2517E+03	2.3168E+03	2.2991E+03	2.2536E+03	2.2573E+03	2.2089E+03	2.2435E+03	2.2975E+03	2.2376E+03	2.3050E+03
	Std	6.0891E+01	5.4333E+01	5.6349E+01	4.6050E+01	4.8552E+01	4.1998E+01	5.4607E+01	5.8464E+01	1.7973E+01	5.4717E+01	6.1910E+01	5.2006E+01	3.8973E+01
	Rank	6	11	12	4	13	9	5	7	1	3	8	2	10
F21	Best	2.2000E+03	2.2457E+03	2.2700E+03	2.3034E+03	2.3053E+03	2.2273E+03	2.2106E+03	2.2006E+03	2.2284E+03	2.2222E+03	2.2175E+03	2.2162E+03	2.2364E+03
	Mean	2.2954E+03	2.6717E+03	2.3110E+03	2.3069E+03	2.3086E+03	2.3059E+03	2.3014E+03	2.2997E+03	2.3031E+03	2.2859E+03	2.3052E+03	2.3001E+03	2.3125E+03
	Std	2.1700E+01	5.6432E+02	7.8124E+00	1.1591E+00	1.0322E+00	1.1422E+01	2.7643E+01	2.6407E+01	2.3092E+01	3.1164E+01	1.8589E+01	3.0183E+01	1.1903E+01
	Rank	2	13	11	9	10	8	5	3	6	1	7	4	12
F22	Best	2.6040E+03	2.6225E+03	2.6241E+03	2.6174E+03	2.6258E+03	2.6090E+03	2.6095E+03	2.6090E+03	2.6096E+03	2.6116E+03	2.6098E+03	2.6184E+03	2.6075E+03
	Mean	2.6167E+03	2.6388E+03	2.6440E+03	2.6300E+03	2.6394E+03	2.6208E+03	2.6216E+03	2.6310E+03	2.6260E+03	2.6284E+03	2.6390E+03	2.6415E+03	2.6238E+03
	Std	7.3938E+00	1.0592E+01	9.1725E+00	4.2499E+00	5.2266E+00	6.2727E+00	7.2342E+00	1.1354E+01	8.5436E+00	1.1810E+01	1.0365E+01	9.8453E+00	8.2745E+00
	Rank	1	9	13	7	11	2	3	8	5	6	10	12	4
F23	Best	2.5000E+03	2.7596E+03	2.5285E+03	2.5693E+03	2.6484E+03	2.5173E+03	2.5114E+03	2.5019E+03	2.5020E+03	2.5076E+03	2.5431E+03	2.5241E+03	2.5769E+03
	Mean	2.6829E+03	2.7736E+03	2.7578E+03	2.7422E+03	2.7608E+03	2.7364E+03	2.7309E+03	2.7110E+03	2.5652E+03	2.7225E+03	2.7519E+03	2.7186E+03	2.7441E+03
	Std	1.0446E+02	7.9772E+00	4.9453E+01	4.4068E+01	2.4606E+01	4.3079E+01	6.7683E+01	8.9174E+01	7.9852E+01	7.6126E+01	5.4700E+01	9.1344E+01	3.3429E+01
	Rank	2	13	11	8	12	7	6	3	1	5	10	4	9
F24	Best	2.8977E+03	2.8980E+03	2.9011E+03	2.8987E+03	2.8992E+03	2.8995E+03	2.9000E+03	2.8980E+03	2.8994E+03	2.8987E+03	2.9002E+03	2.9014E+03	2.9023E+03
	Mean	2.9137E+03	2.9357E+03	2.9356E+03	2.9225E+03	2.9267E+03	2.9404E+03	2.9312E+03	2.9262E+03	2.9311E+03	2.9403E+03	2.9354E+03	2.9354E+03	2.9499E+03
	Std	2.1867E+01	9.7555E+01	1.9631E+01	2.2812E+01	2.2414E+01	1.5085E+01	2.2021E+01	2.7718E+01	2.0325E+01	2.9777E+01	1.8747E+01	1.8847E+01	1.5047E+01
	Rank	1	10	9	2	4	12	6	3	5	11	8	7	13
F25	Best	2.8000E+03	2.8312E+03	2.9097E+03	2.9005E+03	2.9018E+03	2.8321E+03	2.6346E+03	2.8131E+03	2.6314E+03	2.6838E+03	2.8345E+03	2.8850E+03	2.9329E+03
	Mean	2.8907E+03	3.7399E+03	2.9444E+03	2.9015E+03	2.9052E+03	2.9593E+03	2.9489E+03	2.9295E+03	2.9776E+03	2.9917E+03	2.9451E+03	2.9561E+03	3.0150E+03
	Std	1.6190E+02	5.8499E+02	1.9698E+01	4.3452E-01	6.2707E+00	3.8847E+01	5.4520E+01	1.3519E+02	5.5972E+01	9.0282E+01	4.2578E+01	3.6330E+01	6.1682E+01
	Rank	1	13	5	2	3	9	7	4	10	11	6	8	12
F26	Best	3.0889E+03	3.0927E+03	3.0914E+03	3.0892E+03	3.0901E+03	3.0904E+03	3.0898E+03	3.0951E+03	3.0898E+03	3.0901E+03	3.0907E+03	3.0996E+03	3.0907E+03
	Mean	3.0943E+03	3.0992E+03	3.0954E+03	3.0916E+03	3.0934E+03	3.0945E+03	3.0932E+03	3.1107E+03	3.0952E+03	3.0983E+03	3.0939E+03	3.1107E+03	3.0963E+03
	Std	1.9918E+00	2.5350E+00	2.4256E+00	1.2118E+00	2.1711E+00	2.4691E+00	2.2282E+00	2.4243E+01	2.7830E+00	1.5821E+01	1.8997E+00	8.1037E+00	3.7702E+00
	Rank	5	11	8	1	3	6	2	13	7	10	4	12	9
F27	Best	3.1000E+03	3.1050E+03	3.1437E+03	3.1033E+03	3.1049E+03	3.1245E+03	3.1091E+03	2.8253E+03	3.1028E+03	3.1454E+03	3.1746E+03	2.9507E+03	3.1854E+03
	Mean	3.2771E+03	3.2089E+03	3.2390E+03	3.1749E+03	3.2609E+03	3.3027E+03	3.2409E+03	3.2484E+03	3.1990E+03	3.3644E+03	3.2818E+03	3.1999E+03	3.3357E+03
	Std	1.4871E+02	8.8676E+01	8.8562E+01	1.0807E+02	1.1978E+02	1.0905E+02	8.3148E+01	1.4816E+02	4.1595E+01	1.0923E+02	1.1649E+02	8.2571E+01	9.7892E+01
	Rank	9	4	5	1	8	11	6	7	2	13	10	3	12
F28	Best	3.1553E+03	3.1829E+03	3.1792E+03	3.1744E+03	3.1900E+03	3.1453E+03	3.1543E+03	3.1499E+03	3.1466E+03	3.1391E+03	3.1693E+03	3.1858E+03	3.1453E+03
	Mean	3.1894E+03	3.2375E+03	3.2574E+03	3.2108E+03	3.2310E+03	3.1995E+03	3.1953E+03	3.2206E+03	3.1915E+03	3.2452E+03	3.2519E+03	3.2563E+03	3.2206E+03
	Std	1.3754E+01	3.0811E+01	4.2061E+01	1.8685E+01	2.5911E+01	3.8912E+01	2.9087E+01	4.4160E+01	2.8640E+01	5.8961E+01	3.9682E+01	3.8288E+01	6.2874E+01
	Rank	1	9	13	5	8	4	3	7	2	10	11	12	6
F29	Best	3.4049E+03	4.8707E+04	5.4127E+03	3.6705E+03	4.4470E+03	7.1718E+03	3.7800E+03	1.4484E+04	4.0368E+03	4.9301E+03	5.1365E+04	9.8253E+03	1.1253E+04
	Mean	3.6818E+03	8.2578E+05	3.6777E+05	3.9554E+04	1.8435E+05	8.6251E+05	2.6964E+05	8.8983E+05	2.2771E+05	4.0217E+05	7.1647E+05	1.1275E+06	1.2089E+06
	Std	8.2963E+02	1.2615E+06	6.5289E+05	1.6706E+05	3.9090E+05	7.3306E+05	3.2687E+05	1.0793E+06	3.8668E+05	4.2571E+05	3.8801E+05	1.4272E+06	9.2854E+05
	Rank	1	9	6	2	3	10	5	11	4	7	8	12	13

Table A3. Statistical results obtained from LMEDO and comparison algorithms based on CEC2018 (50D)

No.	Index	LMEDO	EDO	LSHADE	LSHADE-SPACMA	APSM-Jso	EO	IRIME	MRFO	GLS-MPA	ECO	ISGTOA	QIO	EPSCA
F1	Best	1.0174E+02	3.7754E+06	4.1458E+07	4.9209E+04	1.3804E+06	5.2792E+07	1.9896E+08	2.2510E+07	9.4569E+08	5.9656E+07	8.0378E+06	2.8420E+08	5.3366E+08
	Mean	2.8437E+03	1.1857E+08	1.2325E+08	1.1337E+05	3.2314E+06	2.5542E+08	4.5429E+08	6.7977E+07	2.3433E+09	3.7586E+08	3.7539E+07	6.2719E+08	1.3168E+09
	Std	3.2449E+03	1.3539E+08	5.5135E+07	3.1711E+04	1.1229E+06	1.9354E+08	1.5674E+08	2.8618E+07	9.5228E+08	2.7436E+08	2.0479E+07	2.1766E+08	3.8768E+08
	Rank	1	6	7	2	3	8	10	5	13	9	4	11	12
F2	Best	3.0000E+02	4.1776E+04	1.2856E+04	4.8097E+02	2.5205E+03	3.8683E+04	4.2941E+04	1.4305E+04	1.9083E+04	1.2524E+04	3.9355E+04	4.5561E+04	5.8217E+04
	Mean	3.0019E+02	8.4414E+04	2.8969E+04	3.5378E+03	6.2675E+03	7.6246E+04	9.0113E+04	2.9885E+04	3.4815E+04	2.5582E+04	5.7430E+04	8.0147E+04	1.4331E+05
	Std	5.0294E-01	1.9885E+04	9.5960E+03	1.5447E+04	2.5627E+03	1.9587E+04	2.3313E+04	6.4852E+03	9.3910E+03	7.0937E+03	1.0291E+04	1.5346E+04	4.8773E+04
	Rank	1	11	5	2	3	9	12	6	7	4	8	10	13
F3	Best	4.0044E+02	4.9372E+02	5.1783E+02	4.8584E+02	4.8978E+02	4.9923E+02	5.2587E+02	4.9728E+02	5.5978E+02	4.9492E+02	4.9395E+02	5.5613E+02	5.7791E+02
	Mean	4.7985E+02	5.6677E+02	5.5107E+02	4.8908E+02	5.0435E+02	5.6528E+02	5.8015E+02	5.6333E+02	7.1396E+02	5.6164E+02	5.4398E+02	6.6066E+02	6.8587E+02
	Std	2.5017E+01	4.1858E+01	1.6199E+01	1.4290E+00	1.2041E+01	3.3442E+01	2.9809E+01	3.0187E+01	1.1282E+02	4.7132E+01	2.5370E+01	5.1053E+01	5.7717E+01
	Rank	1	9	5	2	3	8	10	7	13	6	4	11	12
F4	Best	5.6980E+02	6.9284E+02	6.5225E+02	6.5545E+02	6.6642E+02	5.8325E+02	5.9194E+02	5.9498E+02	6.0009E+02	6.1314E+02	6.6956E+02	6.6513E+02	5.8913E+02
	Mean	6.7183E+02	7.3758E+02	6.9564E+02	6.7918E+02	6.9851E+02	6.2547E+02	6.5057E+02	6.6434E+02	6.5838E+02	6.8516E+02	7.1096E+02	7.2327E+02	6.2825E+02
	Std	3.7598E+01	2.4314E+01	1.7323E+01	1.1780E+01	1.4830E+01	2.0950E+01	2.7514E+01	3.9158E+01	2.5513E+01	4.0841E+01	2.1809E+01	2.7422E+01	1.7658E+01
	Rank	6	13	9	7	10	1	3	5	4	8	11	12	2
F5	Best	6.0000E+02	6.0849E+02	6.0634E+02	6.0062E+02	6.0174E+02	6.0341E+02	6.0682E+02	6.0725E+02	6.1630E+02	6.2509E+02	6.0665E+02	6.1657E+02	6.0725E+02
	Mean	6.0067E+02	6.1859E+02	6.1042E+02	6.0086E+02	6.0270E+02	6.0687E+02	6.1149E+02	6.2337E+02	6.2733E+02	6.4644E+02	6.1414E+02	6.2455E+02	6.1304E+02
	Std	1.2235E+00	6.0591E+00	2.2528E+00	1.4984E-01	5.5456E-01	1.7901E+00	2.7175E+00	1.0015E+01	5.8420E+00	9.3942E+00	4.7001E+00	4.8461E+00	2.5378E+00
	Rank	1	9	5	2	3	4	6	10	12	13	8	11	7
F6	Best	8.5221E+02	9.4195E+02	9.0952E+02	8.8231E+02	9.0641E+02	8.2399E+02	8.8381E+02	9.0550E+02	8.3857E+02	9.2730E+02	9.2774E+02	9.5269E+02	8.4978E+02
	Mean	8.9063E+02	9.9378E+02	9.3947E+02	9.1247E+02	9.3296E+02	8.7589E+02	9.4174E+02	9.8947E+02	9.3874E+02	1.0201E+03	9.6939E+02	9.9201E+02	9.0343E+02
	Std	2.1076E+01	2.5478E+01	1.7121E+01	1.1498E+01	1.1532E+01	2.0703E+01	2.8397E+01	4.9658E+01	4.3522E+01	5.9770E+01	2.1515E+01	2.1561E+01	2.7846E+01
	Rank	2	12	7	4	5	1	8	10	6	13	9	11	3
F7	Best	8.7361E+02	9.8679E+02	9.4992E+02	9.5031E+02	9.3937E+02	8.7350E+02	9.0235E+02	8.6791E+02	8.7619E+02	8.8987E+02	9.2599E+02	9.3759E+02	8.7579E+02
	Mean	9.6149E+02	1.0317E+03	9.9159E+02	9.8181E+02	9.9471E+02	9.1714E+02	9.4377E+02	9.3972E+02	9.4435E+02	9.5471E+02	1.0056E+03	9.9454E+02	9.2547E+02
	Std	2.8163E+01	2.2142E+01	1.8201E+01	1.2967E+01	1.4869E+01	1.9529E+01	2.3056E+01	3.3643E+01	3.0059E+01	2.7127E+01	2.9183E+01	2.4527E+01	1.8032E+01
	Rank	7	13	9	8	11	1	4	3	5	6	12	10	2
F8	Best	9.0000E+02	1.2586E+03	1.0305E+03	9.0081E+02	9.0483E+02	9.5621E+02	1.2923E+03	1.3046E+03	1.5951E+03	2.3945E+03	1.0196E+03	1.2967E+03	1.0400E+03
	Mean	1.0024E+03	3.1969E+03	1.2414E+03	9.0155E+02	9.1454E+02	1.2106E+03	2.1311E+03	4.1912E+03	2.6318E+03	5.1534E+03	1.5483E+03	2.3066E+03	1.5246E+03
	Std	3.8107E+02	1.6874E+03	1.4758E+02	4.6823E-01	8.3689E+00	2.4355E+02	7.3456E+02	1.7946E+03	6.2595E+02	1.4003E+03	4.5752E+02	6.9643E+02	2.4438E+02
	Rank	3	11	5	1	2	4	8	12	10	13	7	9	6
F9	Best	5.7356E+03	5.6128E+03	6.9790E+03	6.5812E+03	7.8658E+03	4.8743E+03	4.9332E+03	4.0724E+03	3.4958E+03	4.4575E+03	7.2410E+03	7.8091E+03	4.5542E+03
	Mean	7.0155E+03	7.1015E+03	8.6544E+03	8.0732E+03	8.7765E+03	6.6468E+03	6.2998E+03	6.5089E+03	5.0786E+03	5.9820E+03	8.5292E+03	8.7151E+03	6.1324E+03
	Std	4.3295E+02	6.0187E+02	5.9968E+02	4.9248E+02	3.1075E+02	6.5513E+02	5.3382E+02	9.4294E+02	6.3651E+02	6.8924E+02	4.2099E+02	3.2233E+02	6.5821E+02
	Rank	7	8	11	9	13	6	4	5	1	2	10	12	3
F10	Best	1.1158E+03	1.5000E+03	1.2674E+03	1.1616E+03	1.2170E+03	1.3029E+03	1.3202E+03	1.3237E+03	1.2956E+03	1.1943E+03	1.6016E+03	1.4512E+03	1.3693E+03
	Mean	1.1688E+03	2.2327E+03	1.3572E+03	1.2140E+03	1.2611E+03	1.4242E+03	1.5132E+03	1.4251E+03	1.5052E+03	1.3426E+03	1.9751E+03	1.6496E+03	1.9982E+03
	Std	3.5667E+01	7.5792E+02	4.4412E+01	2.4601E+01	2.1813E+01	7.3197E+01	8.8189E+01	5.4779E+01	1.0229E+02	6.5604E+01	2.7248E+02	1.4106E+02	5.1025E+02
	Rank	1	13	5	2	3	6	9	7	8	4	11	10	12
F11	Best	2.8769E+03	6.5847E+05	9.1535E+05	3.6102E+04	3.2256E+05	9.6786E+05	7.4550E+06	4.5070E+05	9.2943E+06	1.9754E+06	4.5060E+05	8.8155E+06	6.2675E+06
	Mean	1.7415E+04	9.7212E+06	6.3376E+06	8.0684E+04	1.0154E+06	1.3456E+07	3.0485E+07	3.7532E+06	8.1490E+07	1.4002E+07	5.1028E+06	3.2245E+07	2.6920E+07
	Std	1.1169E+04	1.0305E+07	3.9404E+06	3.0749E+04	4.5996E+05	1.1589E+07	1.8616E+07	2.3603E+06	7.8119E+07	1.1300E+07	3.6813E+06	2.0687E+07	1.9186E+07
	Rank	1	7	6	2	3	8	11	4	13	9	5	12	10
F12	Best	1.3948E+03	6.5511E+03	4.8804E+04	6.4908E+03	4.2070E+04	2.4957E+04	1.9578E+05	8.0326E+03	2.1921E+04	2.4655E+04	2.3107E+04	1.6122E+05	2.6392E+04
	Mean	1.7088E+03	1.7851E+05	2.6762E+05	1.2200E+04	8.1817E+04	1.4071E+05	1.4559E+06	3.4113E+04	6.3964E+05	1.3406E+05	7.4390E+04	6.3340E+05	2.6677E+05
	Std	3.3717E+02	2.8357E+05	1.8909E+05	3.4814E+03	2.2383E+04	7.9773E+04	1.4510E+06	2.4724E+04	1.4650E+06	7.9820E+04	3.7101E+04	3.2935E+05	3.5767E+05
	Rank	1	8	10	2	5	7	13	3	12	6	4	11	9
F13	Best	1.4536E+03	7.9398E+03	1.5855E+03	1.5020E+03	1.5565E+03	3.5686E+03	4.1988E+03	2.5342E+03	1.7011E+03	1.6429E+03	3.0136E+03	1.3610E+04	6.1314E+03
	Mean	1.4765E+03	1.1710E+05	1.9654E+03	1.5356E+03	1.6441E+03	1.0785E+05	9.1682E+04	3.3332E+04	6.7108E+03	1.1683E+04	7.0005E+04	1.1373E+05	3.5775E+05
	Std	1.1176E+01	7.9573E+04	4.0785E+02	1.6592E+01	3.9871E+01	1.2225E+05	7.1817E+04	3.4940E+04	6.7852E+03	1.8006E+04	1.0634E+05	8.1826E+04	4.7764E+05
	Rank	1	12	4	2	3	10	9	7	5	6	8	11	13
F14	Best	1.5377E+03	3.0406E+03	9.2363E+03	1.9147E+03	3.6966E+03	5.3517E+03	6.0227E+03	2.0610E+03	6.3685E+03	4.2272E+03	1.0602E+04	2.0599E+04	4.7810E+03
	Mean	1.6101E+03	6.1558E+04	4.0076E+04	2.2389E+03	9.2710E+03	2.8928E+04	9.7200E+04	9.5820E+03	5.4206E+04	5.3009E+04	3.8991E+04	6.8699E+04	2.9308E+04
	Std	5.6644E+01	1.1104E+05	2.1364E+04	1.5349E+02	2.8560E+03	1.9784E+04	8.0636E+04	8.2139E+03	4.2748E+04	4.5582E+04	1.9809E+04	3.7839E+04	2.6721E+04

	Rank	1	11	8	2	3	5	13	4	10	9	7	12	6
F15	Best	2.3381E+03	3.0514E+03	2.7322E+03	2.7154E+03	2.4648E+03	2.0471E+03	2.1252E+03	2.1796E+03	2.0878E+03	1.9635E+03	2.6278E+03	2.7754E+03	1.7990E+03
	Mean	2.9628E+03	3.5665E+03	3.4067E+03	3.1286E+03	3.3089E+03	2.5659E+03	2.7120E+03	2.7869E+03	2.6889E+03	2.9070E+03	3.3013E+03	3.4413E+03	2.7398E+03
	Std	2.3092E+02	2.1418E+02	2.7634E+02	1.7561E+02	2.6068E+02	3.0288E+02	2.9072E+02	2.8995E+02	2.6223E+02	3.1991E+02	3.1009E+02	2.6719E+02	3.1779E+02
	Rank	7	13	11	8	10	1	3	5	2	6	9	12	4
F16	Best	1.7573E+03	1.9910E+03	1.9854E+03	1.9915E+03	2.0827E+03	1.7914E+03	1.8318E+03	1.7934E+03	1.8624E+03	1.8405E+03	1.8966E+03	1.8977E+03	1.8014E+03
	Mean	2.0013E+03	2.3224E+03	2.3887E+03	2.2121E+03	2.3561E+03	1.9970E+03	2.1171E+03	2.0999E+03	2.0786E+03	2.2762E+03	2.2423E+03	2.1052E+03	2.1729E+03
	Std	1.2855E+02	1.6420E+02	1.9025E+02	1.0329E+02	1.3579E+02	1.5170E+02	1.8587E+02	2.2487E+02	1.3423E+02	2.3091E+02	1.9804E+02	1.6780E+02	2.1233E+02
	Rank	2	11	13	8	12	1	6	4	3	10	9	5	7
F17	Best	1.8433E+03	1.7051E+05	1.4653E+04	2.3617E+03	4.3341E+03	8.0228E+04	1.0373E+05	8.7241E+04	5.4351E+04	2.2834E+04	8.8104E+04	8.6230E+04	1.0776E+05
	Mean	1.8649E+03	1.3233E+06	5.4760E+04	2.8692E+03	1.4074E+04	1.4247E+06	1.4086E+06	5.6505E+05	2.3721E+05	1.9216E+05	9.2423E+05	1.1790E+06	3.1845E+06
	Std	1.3569E+01	1.3705E+06	3.6618E+04	2.5324E+02	7.0895E+03	2.4236E+06	1.5293E+06	5.2182E+05	1.5527E+05	2.7390E+05	7.5242E+05	6.6037E+05	3.5485E+06
	Rank	1	10	4	2	3	12	11	7	6	5	8	9	13
F18	Best	1.9263E+03	3.3492E+03	4.7183E+03	2.0332E+03	2.6653E+03	2.4857E+03	1.7925E+04	2.1048E+03	4.2670E+03	2.5685E+03	4.0596E+03	3.1840E+04	2.8059E+03
	Mean	1.9825E+03	7.7628E+04	2.9050E+04	2.1444E+03	5.3994E+03	2.6451E+04	1.9261E+05	1.0285E+04	1.1856E+05	7.4848E+04	4.8386E+04	1.3634E+05	3.0757E+04
	Std	2.2683E+02	1.8605E+05	2.2443E+04	6.2572E+01	1.7418E+03	5.7963E+04	2.1572E+05	1.0695E+04	1.9780E+05	1.8380E+05	5.0706E+04	8.2551E+04	1.0876E+05
	Rank	1	10	6	2	3	5	13	4	11	9	8	12	7
F19	Best	2.1544E+03	2.4603E+03	2.3967E+03	2.4224E+03	2.4622E+03	2.1165E+03	2.1353E+03	2.2504E+03	2.1742E+03	2.2268E+03	2.3944E+03	2.4519E+03	2.1832E+03
	Mean	2.4327E+03	2.9072E+03	2.7804E+03	2.7033E+03	2.8223E+03	2.3883E+03	2.4587E+03	2.4968E+03	2.3665E+03	2.5439E+03	2.7507E+03	2.7937E+03	2.5651E+03
	Std	1.6577E+02	1.6073E+02	1.7054E+02	1.0854E+02	1.3719E+02	1.4970E+02	1.5604E+02	1.5177E+02	9.7494E+01	1.5877E+02	1.5621E+02	1.7629E+02	1.9682E+02
	Rank	3	13	10	8	12	2	4	5	1	6	9	11	7
F20	Best	2.2000E+03	2.4621E+03	2.4420E+03	2.4446E+03	2.4720E+03	2.3775E+03	2.4024E+03	2.3750E+03	2.3915E+03	2.2344E+03	2.4256E+03	2.2619E+03	2.3844E+03
	Mean	2.4401E+03	2.5189E+03	2.4908E+03	2.4731E+03	2.4957E+03	2.4170E+03	2.4403E+03	2.4300E+03	2.4372E+03	2.4612E+03	2.4960E+03	2.4996E+03	2.4169E+03
	Std	5.1628E+01	2.3507E+01	1.8797E+01	1.1248E+01	9.3088E+00	2.1352E+01	2.0507E+01	2.9660E+01	2.3027E+01	5.1220E+01	2.2130E+01	4.0156E+01	1.4641E+01
	Rank	5	13	9	8	10	2	6	3	4	7	11	12	1
F21	Best	2.3000E+03	7.9892E+03	2.3497E+03	2.3029E+03	2.3176E+03	2.3357E+03	2.3704E+03	2.3302E+03	2.4343E+03	2.3569E+03	2.3214E+03	2.3972E+03	2.4262E+03
	Mean	2.3001E+03	8.6220E+03	2.4070E+03	2.3058E+03	2.3245E+03	3.0841E+03	4.6395E+03	2.4394E+03	2.7024E+03	3.6138E+03	3.0895E+03	2.4876E+03	5.5178E+03
	Std	4.8152E-01	3.3657E+02	2.8764E+01	1.4995E+00	3.9092E+00	1.7806E+03	2.5997E+03	6.2329E+02	1.3375E+02	2.0586E+03	2.0949E+03	4.8470E+01	2.4880E+03
	Rank	1	13	4	2	3	8	11	5	7	10	9	6	12
F22	Best	2.6904E+03	2.8589E+03	2.7711E+03	2.7981E+03	2.8207E+03	2.7221E+03	2.7371E+03	2.7551E+03	2.7531E+03	2.7513E+03	2.8042E+03	2.8471E+03	2.7494E+03
	Mean	2.7952E+03	2.9116E+03	2.8463E+03	2.8253E+03	2.8544E+03	2.7646E+03	2.7943E+03	2.8439E+03	2.8108E+03	2.8556E+03	2.8623E+03	2.9196E+03	2.7817E+03
	Std	2.6654E+01	2.9144E+01	2.1638E+01	1.1865E+01	1.2436E+01	2.3513E+01	2.4602E+01	4.5943E+01	3.1527E+01	5.4954E+01	2.6167E+01	3.1979E+01	2.0377E+01
	Rank	4	12	8	6	9	1	3	7	5	10	11	13	2
F23	Best	2.8454E+03	3.0065E+03	2.9830E+03	2.9533E+03	2.9642E+03	2.8689E+03	2.9197E+03	2.9153E+03	2.9132E+03	2.9048E+03	2.9424E+03	2.9916E+03	2.9154E+03
	Mean	2.9566E+03	3.0944E+03	3.0214E+03	2.9865E+03	3.0165E+03	2.9315E+03	2.9744E+03	3.0004E+03	2.9794E+03	3.0050E+03	3.0181E+03	3.0849E+03	2.9549E+03
	Std	3.4472E+01	4.2246E+01	1.9260E+01	1.2570E+01	1.5488E+01	2.1750E+01	3.0395E+01	4.4032E+01	3.1690E+01	6.4839E+01	2.8898E+01	3.5440E+01	2.0395E+01
	Rank	3	13	11	6	9	1	4	7	5	8	10	12	2
F24	Best	2.8834E+03	2.8914E+03	2.9054E+03	2.8871E+03	2.8881E+03	2.9196E+03	2.9125E+03	2.9014E+03	2.9582E+03	2.9064E+03	2.9138E+03	2.9403E+03	2.9629E+03
	Mean	2.8864E+03	2.9391E+03	2.9324E+03	2.8873E+03	2.8939E+03	2.9614E+03	2.9715E+03	2.9517E+03	3.0359E+03	2.9766E+03	2.9528E+03	2.9966E+03	3.0402E+03
	Std	3.4663E+00	2.3369E+01	1.4924E+01	2.2291E-01	6.0009E+00	2.4507E+01	3.0895E+01	2.7590E+01	5.1079E+01	3.7350E+01	2.0895E+01	2.8104E+01	4.0060E+01
	Rank	1	5	4	2	3	8	9	6	12	10	7	11	13
F25	Best	2.8000E+03	5.6460E+03	5.0193E+03	5.0145E+03	5.2001E+03	3.3334E+03	3.2872E+03	2.9841E+03	3.6610E+03	3.1093E+03	2.9432E+03	3.2932E+03	4.4932E+03
	Mean	4.5962E+03	6.2609E+03	5.5307E+03	5.2634E+03	5.5087E+03	4.6449E+03	5.0813E+03	5.3558E+03	4.7530E+03	5.6826E+03	5.2242E+03	5.6237E+03	5.0769E+03
	Std	8.5226E+02	2.8372E+02	1.8529E+02	1.1509E+02	1.2654E+02	3.1800E+02	4.2862E+02	1.2140E+03	8.2649E+02	7.6393E+02	1.0879E+03	1.2134E+03	2.6217E+02
	Rank	1	13	10	7	9	2	5	8	3	12	6	11	4
F26	Best	3.1806E+03	3.2476E+03	3.2299E+03	3.2001E+03	3.2176E+03	3.2051E+03	3.2094E+03	3.2458E+03	3.2141E+03	3.2086E+03	3.2137E+03	3.3262E+03	3.2200E+03
	Mean	3.2191E+03	3.2901E+03	3.2492E+03	3.2159E+03	3.2329E+03	3.2318E+03	3.2354E+03	3.3051E+03	3.2591E+03	3.2626E+03	3.2436E+03	3.4047E+03	3.2495E+03
	Std	1.5683E+01	2.9880E+01	1.1591E+01	5.5881E+00	7.5732E+00	1.0824E+01	1.0982E+01	3.0646E+01	2.5302E+01	3.0396E+01	1.3904E+01	4.7576E+01	2.1634E+01
	Rank	2	11	7	1	4	3	5	12	9	10	6	13	8
F27	Best	3.1000E+03	3.2197E+03	3.2715E+03	3.2036E+03	3.2132E+03	3.2593E+03	3.2958E+03	3.2393E+03	3.3367E+03	3.3042E+03	3.2745E+03	3.3114E+03	3.3762E+03
	Mean	3.1872E+03	3.3132E+03	3.3112E+03	3.2210E+03	3.2482E+03	3.3517E+03	3.3588E+03	3.3183E+03	3.4692E+03	3.3809E+03	3.3216E+03	3.4270E+03	3.5275E+03
	Std	4.2447E+01	6.1575E+01	2.2857E+01	8.7273E+00	1.6247E+01	3.9398E+01	3.5816E+01	3.2438E+01	7.2434E+01	6.2316E+01	3.3096E+01	5.4292E+01	9.8621E+01
	Rank	1	5	4	2	3	8	9	6	12	10	7	11	13
F28	Best	3.4665E+03	3.7459E+03	3.7735E+03	3.7615E+03	3.9257E+03	3.5351E+03	3.5838E+03	3.5905E+03	3.5190E+03	3.7639E+03	3.7937E+03	3.7936E+03	3.6820E+03
	Mean	3.7086E+03	4.1922E+03	4.1956E+03	3.9579E+03	4.1906E+03	3.7779E+03	3.8885E+03	4.0143E+03	3.9508E+03	4.3505E+03	4.2746E+03	4.2129E+03	4.0051E+03
	Std	1.1809E+02	2.4896E+02	2.1690E+02	8.0070E+01	1.2446E+02	1.5487E+02	1.6472E+02	2.0148E+02	2.1414E+02	2.6455E+02	2.1500E+02	2.4207E+02	1.8842E+02
	Rank	1	9	10	5	8	2	3	7	4	13	12	11	6
F29	Best	5.4336E+03	4.2474E+04	7.6985E+04	7.7897E+03	2.9361E+04	5.8746E+04	9.6976E+04	3.4353E+04	2.8275E+05	6.6697E+04	5.6880E+04	5.9889E+05	6.0669E+04
	Mean	6.4650E+03	5.2723E+05	4.3506E+05	1.1825E+04	5.4621E+04	6.4037E+05	9.8104E+05	1.8982E+05	1.9495E+06	8.7207E+05	3.9371E+05	2.2077E+06	7.5972E+05
	Std	1.3863E+03	6.1630E+05	3.1537E+05	2.7710E+03	1.7871E+04	6.0373E+05	7.3048E+05	1.8077E+05	1.3084E+06	6.2203E+05	3.4201E+05	1.2392E+06	5.3087E+05
	Rank	1	7	6	2	3	8	11	4	12	10	5	13	9

Table A4. Statistical results obtained from LMEDO and comparison algorithms based on CEC2018 (100D)

No.	Index	LMEDO	EDO	LSHADE	LSHADE-SPACMA	APSM-Jso	EO	IRIME	MRFO	GLS-MPA	ECO	ISGTOA	QIO	EPSCA
F1	Best	1.9039E+02	2.8482E+09	6.7908E+08	2.6924E+05	1.4632E+08	1.3477E+10	1.4252E+10	7.0223E+09	3.7040E+10	1.0435E+10	4.5989E+09	1.2135E+10	3.3163E+10
	Mean	8.1322E+03	8.2758E+09	1.6016E+09	7.4212E+05	2.2839E+08	2.2169E+10	2.0170E+10	1.3042E+10	5.4938E+10	1.9215E+10	8.4885E+09	1.7617E+10	4.7331E+10
	Std	8.6070E+03	4.1959E+09	3.6460E+08	1.8952E+05	5.7225E+07	5.1406E+09	3.6057E+09	3.4348E+09	9.6477E+09	6.3673E+09	2.2478E+09	2.8833E+09	6.8111E+09
	Rank	1	5	4	2	3	11	10	7	13	9	6	8	12
F2	Best	1.3567E+03	3.6625E+05	1.5151E+05	2.7108E+04	8.6035E+04	3.7457E+05	3.4319E+05	2.4148E+05	2.0408E+05	1.4172E+05	2.4520E+05	3.2266E+05	3.7910E+05
	Mean	3.8874E+03	4.8258E+05	2.0942E+05	4.4399E+04	1.1189E+05	4.7741E+05	5.3721E+05	2.9018E+05	2.5936E+05	1.8795E+05	3.0218E+05	3.9494E+05	6.9156E+05
	Std	1.8668E+03	5.8209E+04	2.2700E+04	1.5312E+04	1.7638E+04	7.3736E+04	1.1512E+05	2.8075E+04	2.3843E+04	2.1127E+04	2.2788E+04	3.5966E+04	1.3268E+05
	Rank	1	11	5	2	3	10	12	7	6	4	8	9	13
F3	Best	5.6253E+02	1.3214E+03	8.7509E+02	6.1673E+02	7.7964E+02	1.4973E+03	1.9960E+03	1.5438E+03	3.2257E+03	1.6823E+03	1.1679E+03	2.3306E+03	2.6821E+03
	Mean	6.6346E+02	2.4292E+03	1.0235E+03	6.6122E+02	8.6337E+02	2.3684E+03	2.8206E+03	2.4774E+03	5.8660E+03	2.6953E+03	1.6960E+03	3.1450E+03	4.7508E+03
	Std	4.2046E+01	8.2594E+02	5.4962E+01	1.7065E+01	3.8778E+01	4.4118E+02	5.3329E+02	4.8380E+02	1.4020E+03	5.5499E+02	2.2862E+02	4.6145E+02	1.0496E+03
	Rank	2	7	4	1	3	6	10	8	13	9	5	11	12
F4	Best	1.0333E+03	1.5759E+03	1.1383E+03	8.1196E+02	1.2928E+03	1.0904E+03	1.2142E+03	1.2051E+03	1.2398E+03	1.2114E+03	1.1920E+03	1.3768E+03	1.1339E+03
	Mean	1.3421E+03	1.6873E+03	1.2641E+03	1.1881E+03	1.3389E+03	1.2550E+03	1.3395E+03	1.3618E+03	1.4397E+03	1.4033E+03	1.4555E+03	1.5378E+03	1.2382E+03
	Std	1.6872E+02	6.1735E+01	4.1766E+01	1.1809E+02	2.4304E+01	6.1720E+01	6.2730E+01	6.8618E+01	7.8514E+01	9.3270E+01	1.3630E+02	7.5728E+01	4.6713E+01
	Rank	7	13	4	1	5	3	6	8	10	9	11	12	2
F5	Best	6.0127E+02	6.4276E+02	6.1172E+02	6.0054E+02	6.0396E+02	6.1939E+02	6.3182E+02	6.4860E+02	6.4662E+02	6.5814E+02	6.3909E+02	6.4496E+02	6.3293E+02
	Mean	6.1367E+02	6.5618E+02	6.1647E+02	6.0074E+02	6.0595E+02	6.2697E+02	6.3947E+02	6.6659E+02	6.5690E+02	6.7010E+02	6.4774E+02	6.5928E+02	6.3996E+02
	Std	9.4392E+00	8.0558E+00	2.2369E+00	1.3263E-01	1.1237E+00	3.7987E+00	4.3082E+00	7.3355E+00	5.2764E+00	5.6125E+00	5.2952E+00	6.4784E+00	4.1348E+00
	Rank	3	9	4	1	2	5	6	12	10	13	8	11	7
F6	Best	1.5542E+03	2.1014E+03	1.5207E+03	8.7148E+02	1.6135E+03	1.6200E+03	1.8838E+03	2.3444E+03	1.9432E+03	2.3373E+03	2.0108E+03	2.0107E+03	1.9087E+03
	Mean	1.6739E+03	2.3081E+03	1.6338E+03	1.0973E+03	1.6679E+03	1.8943E+03	2.0784E+03	2.6867E+03	2.3413E+03	2.9037E+03	2.1256E+03	2.1572E+03	2.1429E+03
	Std	7.2612E+01	1.2299E+02	4.9049E+01	1.3606E+02	2.4360E+01	9.8276E+01	9.4884E+01	2.0730E+02	1.5200E+02	2.6996E+02	5.8647E+01	7.0727E+01	9.9461E+01
	Rank	4	10	2	1	3	5	6	12	11	13	7	9	8
F7	Best	1.1791E+03	1.8459E+03	1.4092E+03	1.3487E+03	1.5770E+03	1.4319E+03	1.4984E+03	1.5938E+03	1.5747E+03	1.6124E+03	1.3946E+03	1.6308E+03	1.4463E+03
	Mean	1.6201E+03	1.9664E+03	1.5754E+03	1.5610E+03	1.6436E+03	1.5415E+03	1.6282E+03	1.7540E+03	1.7353E+03	1.7715E+03	1.7268E+03	1.8681E+03	1.5582E+03
	Std	1.5293E+02	5.4790E+01	4.1979E+01	4.2076E+01	2.3979E+01	6.4001E+01	6.1399E+01	9.6160E+01	8.4877E+01	8.1909E+01	1.3870E+02	7.3196E+01	4.8999E+01
	Rank	5	13	4	3	7	1	6	10	9	11	8	12	2
F8	Best	1.0043E+03	2.9494E+04	2.6048E+03	9.0385E+02	1.1990E+03	1.2624E+04	2.1541E+04	3.3338E+04	1.9222E+04	2.8335E+04	2.2338E+04	2.7119E+04	1.1001E+04
	Mean	2.0021E+04	5.7705E+04	4.7207E+03	9.1087E+02	1.5442E+03	2.3376E+04	3.5112E+04	4.9537E+04	2.9684E+04	3.7571E+04	3.3702E+04	4.2141E+04	1.8672E+04
	Std	1.7286E+04	1.3862E+04	1.2731E+03	3.3300E+00	2.4020E+02	5.4676E+03	7.7585E+03	9.7412E+03	4.4641E+03	4.6998E+03	5.6398E+03	7.1134E+03	3.4134E+03
	Rank	5	13	3	1	2	6	9	12	7	10	8	11	4
F9	Best	2.4845E+04	2.6423E+04	2.7083E+04	3.0464E+04	3.0262E+04	2.1778E+04	2.0638E+04	1.7607E+04	1.7969E+04	1.7825E+04	3.0063E+04	3.0340E+04	2.1551E+04
	Mean	2.6647E+04	2.8417E+04	3.0109E+04	3.1521E+04	3.1940E+04	2.4850E+04	2.4635E+04	2.2017E+04	2.0667E+04	2.1125E+04	3.1843E+04	3.1960E+04	2.4456E+04
	Std	6.5554E+02	9.5987E+02	1.3049E+03	5.7431E+02	6.5569E+02	1.5363E+03	1.4428E+03	2.5640E+03	1.0524E+03	2.2600E+03	6.6062E+02	5.1851E+02	1.1526E+03
	Rank	7	8	9	10	12	6	5	3	1	2	11	13	4
F10	Best	1.6373E+03	6.6179E+04	6.8318E+03	2.0960E+03	2.5074E+03	2.3401E+04	3.9994E+04	4.5649E+04	1.8734E+04	1.0047E+04	5.7952E+04	5.5613E+04	5.9932E+04
	Mean	1.7980E+03	1.3329E+05	1.1112E+04	2.3352E+03	3.4143E+03	4.7031E+04	9.3355E+04	6.5737E+04	3.5351E+04	1.7685E+04	8.3479E+04	9.5512E+04	8.9234E+04
	Std	9.8185E+01	3.5513E+04	3.1779E+03	1.5105E+02	3.7495E+02	1.3636E+04	2.6061E+04	1.0321E+04	1.0524E+04	4.7357E+03	1.0493E+04	1.5321E+04	2.1935E+04
	Rank	1	13	4	2	3	7	11	8	6	5	9	12	10
F11	Best	3.3890E+05	2.1311E+08	1.2670E+08	2.5765E+06	2.6013E+07	5.2340E+08	8.5148E+08	3.7023E+08	3.8136E+09	2.7880E+08	2.4342E+08	1.7722E+09	2.5926E+09
	Mean	1.3669E+06	1.0341E+09	2.5900E+08	6.3738E+06	6.6068E+07	1.1748E+09	2.1173E+09	7.5079E+08	8.3389E+09	9.9212E+08	5.7400E+08	2.7099E+09	4.3868E+09
	Std	7.5527E+05	6.2939E+08	7.3264E+07	3.0423E+06	2.1224E+07	3.4232E+08	5.8307E+08	2.1898E+08	3.0018E+09	4.2799E+08	2.2311E+08	6.2630E+08	1.1407E+09
	Rank	1	8	4	2	3	9	10	6	13	7	5	11	12
F12	Best	2.6080E+03	6.8766E+04	6.7806E+04	2.0105E+04	2.4911E+04	4.2993E+05	9.7054E+06	2.1165E+05	5.8462E+07	2.7910E+05	3.9007E+04	1.9528E+07	2.5702E+07
	Mean	6.6744E+03	2.5645E+06	2.7226E+05	2.9318E+04	4.1499E+04	2.4570E+06	2.9287E+07	4.8803E+05	4.0078E+08	1.2441E+06	8.2594E+04	3.4866E+07	7.6241E+07
	Std	4.4483E+03	7.2204E+06	1.1805E+05	5.0287E+03	8.8504E+03	1.5256E+06	1.1566E+07	1.6661E+05	2.7853E+08	1.6040E+06	2.5716E+04	9.6640E+06	4.4196E+07
	Rank	1	9	5	2	3	8	10	6	13	7	4	11	12
F13	Best	1.6245E+03	1.5763E+06	3.5068E+04	1.9289E+03	3.6765E+03	1.3426E+06	1.3844E+06	7.0599E+05	5.5344E+05	1.9857E+05	1.3659E+06	1.3432E+06	2.7704E+06
	Mean	1.8190E+03	1.1237E+07	1.1990E+05	2.1080E+03	1.0369E+04	4.8148E+06	5.1932E+06	1.9466E+06	2.9720E+06	9.5603E+05	3.9180E+06	4.0788E+06	8.9705E+06
	Std	8.2139E+01	7.0265E+06	8.2628E+04	9.8876E+01	6.0018E+03	2.1326E+06	2.3790E+06	8.8604E+05	1.2573E+06	5.5844E+05	1.5977E+06	1.7380E+06	4.6040E+06
	Rank	1	13	4	2	3	10	11	6	7	5	8	9	12
F14	Best	1.7689E+03	6.8311E+03	2.6767E+04	6.0855E+03	2.0326E+04	2.5443E+04	4.0368E+05	1.5263E+04	1.5559E+06	2.4365E+04	1.6640E+04	1.2553E+06	4.1373E+05
	Mean	3.6640E+03	5.3927E+04	5.8513E+04	8.4196E+03	2.9294E+04	9.0955E+04	2.4640E+06	3.0149E+04	4.4609E+07	1.4105E+05	4.5574E+04	2.4130E+06	1.8264E+06
	Std	2.4068E+03	7.6243E+04	2.3266E+04	1.3709E+03	5.6179E+03	2.6802E+04	1.7301E+06	8.8715E+03	7.0569E+07	4.7149E+05	1.9668E+04	8.2862E+05	1.1289E+06
	Rank	1	6	7	2	3	8	12	4	13	9	5	11	10
F15	Best	3.3300E+03	9.7137E+03	7.8036E+03	7.5159E+03	8.4344E+03	5.0861E+03	5.9526E+03	4.6857E+03	6.2508E+03	5.6868E+03	5.0178E+03	5.8544E+03	6.2533E+03
	Mean	7.6512E+03	1.0652E+04	8.8278E+03	8.7291E+03	9.4811E+03	6.3585E+03	6.9530E+03	6.4198E+03	7.5574E+03	7.0508E+03	7.2432E+03	7.9146E+03	7.1570E+03
	Std	1.4032E+03	4.4222E+02	5.0041E+02	4.4587E+02	3.9526E+02	6.8047E+02	5.7418E+02	7.4077E+02	7.3109E+02	7.1122E+02	1.2635E+03	1.0030E+03	5.3416E+02
	Rank	8	13	11	10	12	1	3	2	7	4	6	9	5
F16	Best	5.3993E+03	6.0880E+03	5.1553E+03	5.8150E+03	6.0246E+03	3.5644E+03	4.2680E+03	3.9996E+03	5.0171E+03	4.4133E+03	3.7937E+03	4.4507E+03	3.7885E+03
	Mean	6.2227E+03	7.1776E+03	6.4209E+03	6.4520E+03	6.8967E+03	5.0543E+03	5.4971E+03	5.2236E+03	6.1451E+03	5.5837E+03	5.7007E+03	6.3020E+03	5.1708E+03
	Std	2.3343E+02	3.7481E+02	4.3147E+02	2.4825E+02	2.7850E+02	4.8198E+02	6.0292E+02	5.3448E+02	5.7424E+02	6.3941E+02	9.2054E+02	7.6602E+02	5.1795E+02
	Rank	8	13	10	11	12	1	4	3	7	5	6	9	2
F17	Best	2.0593E+03	5.9989E+06	9.7886E+04	7.9717E+03	3.9117E+04	2.3669E+06	2.3220E+06	7.2990E+05	8.7655E+05	2.4755E+05	1.3425E+06	1.1614E+06	2.0044E+06
	Mean	2.2944E+03	2.9442E+07	2.3158E+05	1.5735E+04	8.2338E+04	4.9171E+06	8.9242E+06	2.9812E+06	3.7031E+06	1.4626E+06	5.0204E+06	4.5330E+06	1.1867E+07

	Std	1.3164E+02	1.8995E+07	8.5320E+04	5.0744E+03	2.0123E+04	1.8211E+06	4.8258E+06	1.6718E+06	1.9527E+06	7.9517E+05	2.1427E+06	1.4522E+06	6.6703E+06
	Rank	1	13	4	2	3	9	11	6	7	5	10	8	12
F18	Best	2.0413E+03	6.5059E+03	1.0333E+05	3.0211E+03	3.6946E+04	9.4733E+04	1.1809E+06	1.5515E+04	9.9204E+06	6.8348E+04	5.8840E+04	1.5792E+06	9.5681E+05
	Mean	4.8714E+03	1.4251E+05	3.6852E+05	4.3651E+03	8.8078E+04	9.7395E+05	7.6361E+06	5.4681E+04	5.9065E+07	4.9488E+05	3.1023E+05	3.7293E+06	3.7999E+06
	Std	4.6497E+03	1.6753E+05	2.1105E+05	9.1965E+02	2.7703E+04	6.8534E+05	3.9228E+06	3.3726E+04	4.6303E+07	5.3399E+05	2.8198E+05	1.3122E+06	2.2901E+06
	Rank	2	5	7	1	4	9	12	3	13	8	6	10	11
F19	Best	6.0529E+03	5.2483E+03	5.9894E+03	6.1879E+03	6.8923E+03	4.1896E+03	4.7744E+03	4.1877E+03	4.2215E+03	3.8896E+03	5.9087E+03	6.1647E+03	4.2839E+03
	Mean	6.5796E+03	6.8012E+03	7.0335E+03	7.0933E+03	7.4162E+03	5.3305E+03	5.6196E+03	5.4924E+03	5.1755E+03	5.4534E+03	7.2889E+03	7.3758E+03	5.3066E+03
	Std	2.3868E+02	5.1887E+02	4.2390E+02	2.9568E+02	2.3508E+02	5.3362E+02	4.4864E+02	5.1920E+02	3.7580E+02	4.7984E+02	3.9235E+02	4.2022E+02	4.6137E+02
	Rank	7	8	9	10	13	3	6	5	1	4	11	12	2
F20	Best	3.0152E+03	3.4060E+03	2.9631E+03	3.0412E+03	3.0943E+03	2.9018E+03	3.0159E+03	2.9851E+03	3.0694E+03	3.0986E+03	2.9820E+03	3.2622E+03	2.9013E+03
	Mean	3.0846E+03	3.5238E+03	3.1039E+03	3.1025E+03	3.1613E+03	2.9909E+03	3.1596E+03	3.2248E+03	3.2283E+03	3.3220E+03	3.2631E+03	3.4354E+03	3.0505E+03
	Std	3.5289E+01	6.6801E+01	4.7963E+01	2.2445E+01	2.4375E+01	5.2039E+01	7.1383E+01	1.1054E+02	7.5870E+01	1.2813E+02	1.3876E+02	5.7140E+01	5.7677E+01
	Rank	3	13	5	4	7	1	6	8	9	11	10	12	2
F21	Best	2.3002E+03	2.9937E+04	2.7169E+04	3.1971E+04	3.2772E+04	2.2314E+04	2.4821E+04	2.0132E+04	9.6968E+03	2.1123E+04	3.0733E+04	1.1751E+04	2.4255E+04
	Mean	1.5617E+04	3.1939E+04	3.2629E+04	3.3809E+04	3.4327E+04	2.7087E+04	2.7122E+04	2.4328E+04	2.2912E+04	2.4979E+04	3.4066E+04	3.3647E+04	2.6442E+04
	Std	1.3318E+04	6.1689E+02	1.5747E+03	5.8227E+02	6.0615E+02	2.0315E+03	1.1756E+03	2.6585E+03	2.5793E+03	1.8100E+03	7.9020E+02	4.2479E+03	1.3381E+03
	Rank	1	8	9	11	13	6	7	3	2	4	12	10	5
F22	Best	3.0552E+03	4.0024E+03	3.5399E+03	3.5397E+03	3.6639E+03	3.3432E+03	3.4463E+03	3.7561E+03	3.6192E+03	3.7219E+03	3.4706E+03	4.2620E+03	3.4907E+03
	Mean	3.6185E+03	4.2066E+03	3.6888E+03	3.6062E+03	3.7134E+03	3.4718E+03	3.5621E+03	4.0351E+03	3.8259E+03	3.9935E+03	3.7049E+03	4.4793E+03	3.5991E+03
	Std	1.7783E+02	1.1824E+02	6.6792E+01	2.5727E+01	2.6028E+01	7.1176E+01	5.9819E+01	1.2599E+02	8.4944E+01	1.5729E+02	1.3896E+02	1.0521E+02	4.9773E+01
	Rank	5	12	6	4	8	1	2	11	9	10	7	13	3
F23	Best	3.4309E+03	4.5347E+03	3.9527E+03	3.9697E+03	4.0020E+03	3.7860E+03	4.0356E+03	4.5316E+03	4.2745E+03	4.2503E+03	3.9366E+03	4.8790E+03	4.0347E+03
	Mean	3.9728E+03	4.7774E+03	4.1358E+03	4.0306E+03	4.1256E+03	3.9176E+03	4.1701E+03	4.9885E+03	4.4749E+03	4.7070E+03	4.2844E+03	5.4448E+03	4.1951E+03
	Std	2.4630E+02	1.1301E+02	6.8921E+01	2.0143E+01	3.4919E+01	5.8267E+01	7.0629E+01	1.8895E+02	9.9832E+01	2.3180E+02	1.5403E+02	2.4316E+02	8.1222E+01
	Rank	2	11	5	3	4	1	6	12	9	10	8	13	7
F24	Best	3.1473E+03	3.9460E+03	3.5617E+03	3.2655E+03	3.4425E+03	4.4486E+03	4.7461E+03	4.2197E+03	5.4602E+03	4.1796E+03	4.0443E+03	4.5951E+03	5.8700E+03
	Mean	3.2956E+03	4.7751E+03	3.7374E+03	3.3295E+03	3.5558E+03	5.0930E+03	5.3315E+03	4.7752E+03	7.0468E+03	5.0118E+03	4.3424E+03	5.2284E+03	7.1847E+03
	Std	5.7025E+01	5.2535E+02	7.7101E+01	3.8170E+01	5.6789E+01	3.8876E+02	3.2012E+02	3.0504E+02	7.5933E+02	3.3630E+02	1.6650E+02	2.5672E+02	6.5814E+02
	Rank	1	6	4	2	3	9	11	7	12	8	5	10	13
F25	Best	2.9005E+03	1.7981E+04	1.2755E+04	1.2534E+04	1.3137E+04	1.1648E+04	1.3425E+04	1.1364E+04	1.5045E+04	1.2248E+04	1.3100E+04	8.6367E+03	1.3408E+04
	Mean	1.2393E+04	1.9519E+04	1.3911E+04	1.3179E+04	1.3849E+04	1.3150E+04	1.4894E+04	2.1132E+04	1.7597E+04	2.0499E+04	1.6040E+04	2.0665E+04	1.4892E+04
	Std	3.9313E+03	1.0128E+03	5.8444E+02	2.7478E+02	2.9941E+02	7.5886E+02	7.2496E+02	3.0221E+03	1.2510E+03	3.3552E+03	1.6826E+03	2.2329E+03	6.7913E+02
	Rank	1	10	5	3	4	2	7	13	9	11	8	12	6
F26	Best	3.3113E+03	4.1785E+03	3.5330E+03	3.2712E+03	3.3837E+03	3.6027E+03	3.6822E+03	3.9961E+03	3.8210E+03	3.6598E+03	3.6583E+03	4.3938E+03	3.7677E+03
	Mean	3.3787E+03	4.4357E+03	3.6190E+03	3.3479E+03	3.4675E+03	3.7491E+03	3.8257E+03	4.4290E+03	4.0951E+03	4.0004E+03	3.8488E+03	5.0017E+03	3.9706E+03
	Std	3.9404E+01	1.7638E+02	5.8070E+01	2.1362E+01	3.9814E+01	8.6264E+01	8.9121E+01	2.0199E+02	1.7276E+02	2.3603E+02	1.3793E+02	2.8709E+02	1.1603E+02
	Rank	2	12	4	1	3	5	6	11	10	9	7	13	8
F27	Best	3.3464E+03	4.5162E+03	3.7607E+03	3.3705E+03	3.5219E+03	5.0941E+03	5.1453E+03	4.6191E+03	7.3354E+03	4.6095E+03	4.2494E+03	4.9540E+03	8.4316E+03
	Mean	3.4137E+03	6.0802E+03	3.9824E+03	3.4101E+03	3.6518E+03	6.5554E+03	6.2021E+03	6.0742E+03	9.6338E+03	6.0265E+03	5.2688E+03	6.2534E+03	1.0590E+04
	Std	3.6302E+01	1.0018E+03	1.2628E+02	2.9708E+01	6.3827E+01	7.8460E+02	5.9429E+02	6.0720E+02	1.2516E+03	6.0496E+02	5.7552E+02	5.1903E+02	1.2839E+03
	Rank	2	8	4	1	3	11	9	7	12	6	5	10	13
F28	Best	4.9516E+03	9.0545E+03	7.7525E+03	7.1975E+03	8.0661E+03	6.2399E+03	7.0229E+03	7.0681E+03	7.7767E+03	6.5094E+03	7.2000E+03	8.3732E+03	6.8890E+03
	Mean	7.9656E+03	1.0566E+04	8.7320E+03	7.9849E+03	8.8033E+03	7.4235E+03	8.0352E+03	8.4953E+03	9.3613E+03	8.8154E+03	8.4704E+03	9.6679E+03	8.2918E+03
	Std	6.1074E+02	5.1137E+02	5.0407E+02	2.9960E+02	3.7980E+02	5.3118E+02	5.4084E+02	6.6111E+02	7.6129E+02	7.7275E+02	8.0683E+02	6.6881E+02	5.7671E+02
	Rank	2	13	8	3	9	1	4	7	11	10	6	12	5
F29	Best	9.5898E+03	1.3460E+06	8.0443E+05	3.4454E+04	5.5693E+05	6.1369E+06	2.3798E+07	2.4016E+06	1.2665E+08	4.9109E+06	4.0919E+06	3.0555E+07	1.8676E+07
	Mean	1.8982E+04	1.0608E+07	4.2486E+06	8.4232E+04	1.1352E+06	2.0137E+07	6.4361E+07	5.9925E+06	4.2566E+08	2.0554E+07	1.1565E+07	7.1412E+07	5.6246E+07
	Std	2.5116E+04	8.8820E+06	1.7374E+06	2.3618E+04	4.2837E+05	9.0915E+06	2.0113E+07	3.0380E+06	1.9035E+08	1.1438E+07	6.3844E+06	1.9882E+07	2.7892E+07
	Rank	1	6	4	2	3	8	11	5	13	9	7	12	10

Appendix B

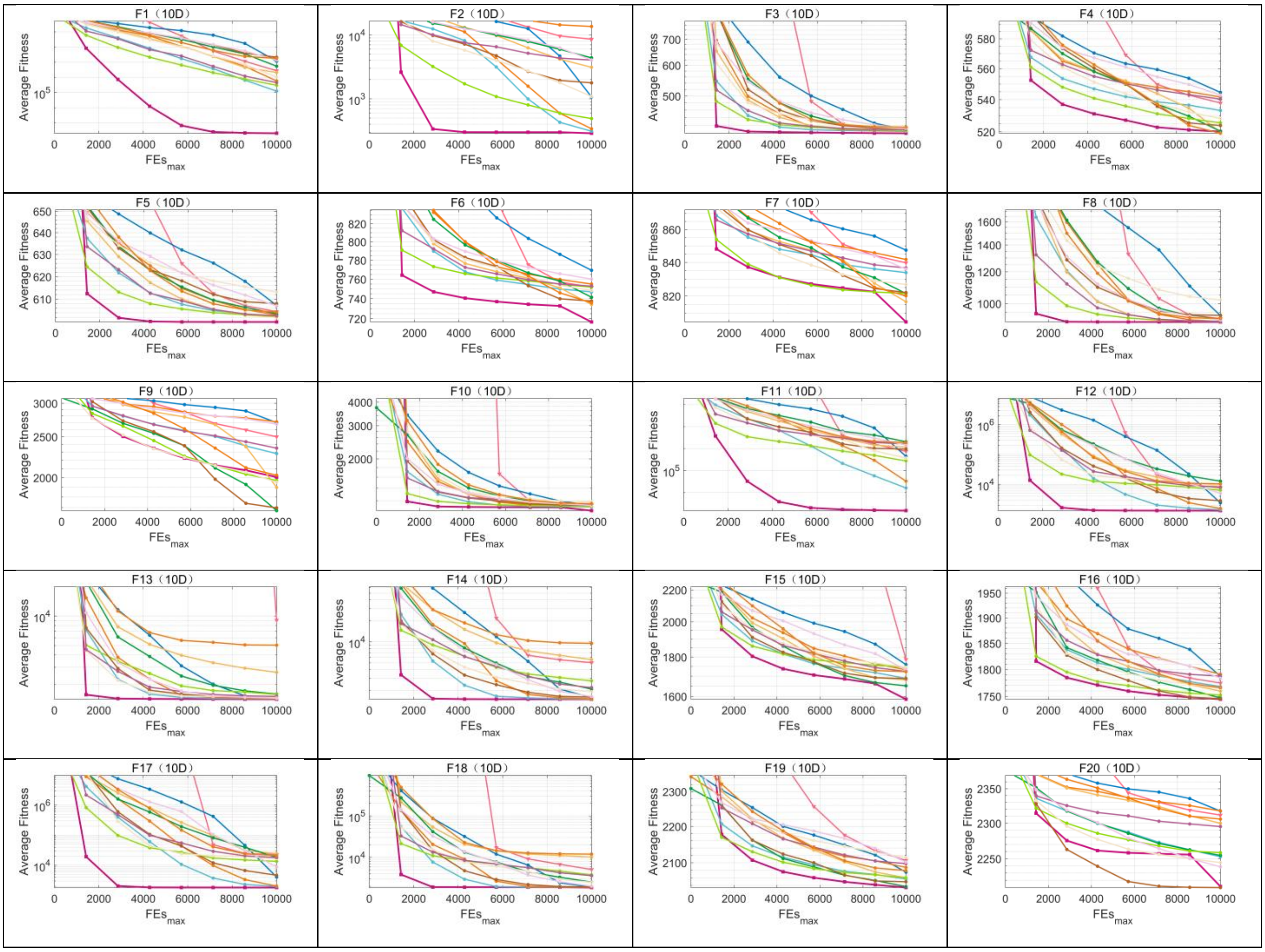

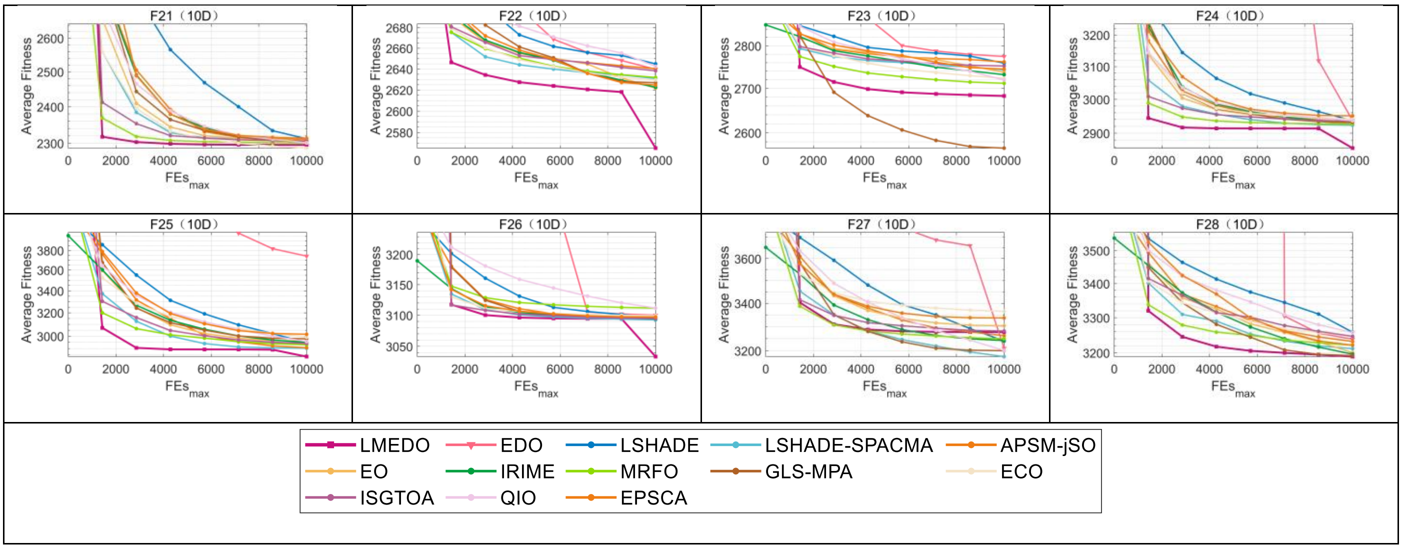

Figure B1. The convergence curves of LMEDO and comparison algorithms based on CEC2018 (10D)

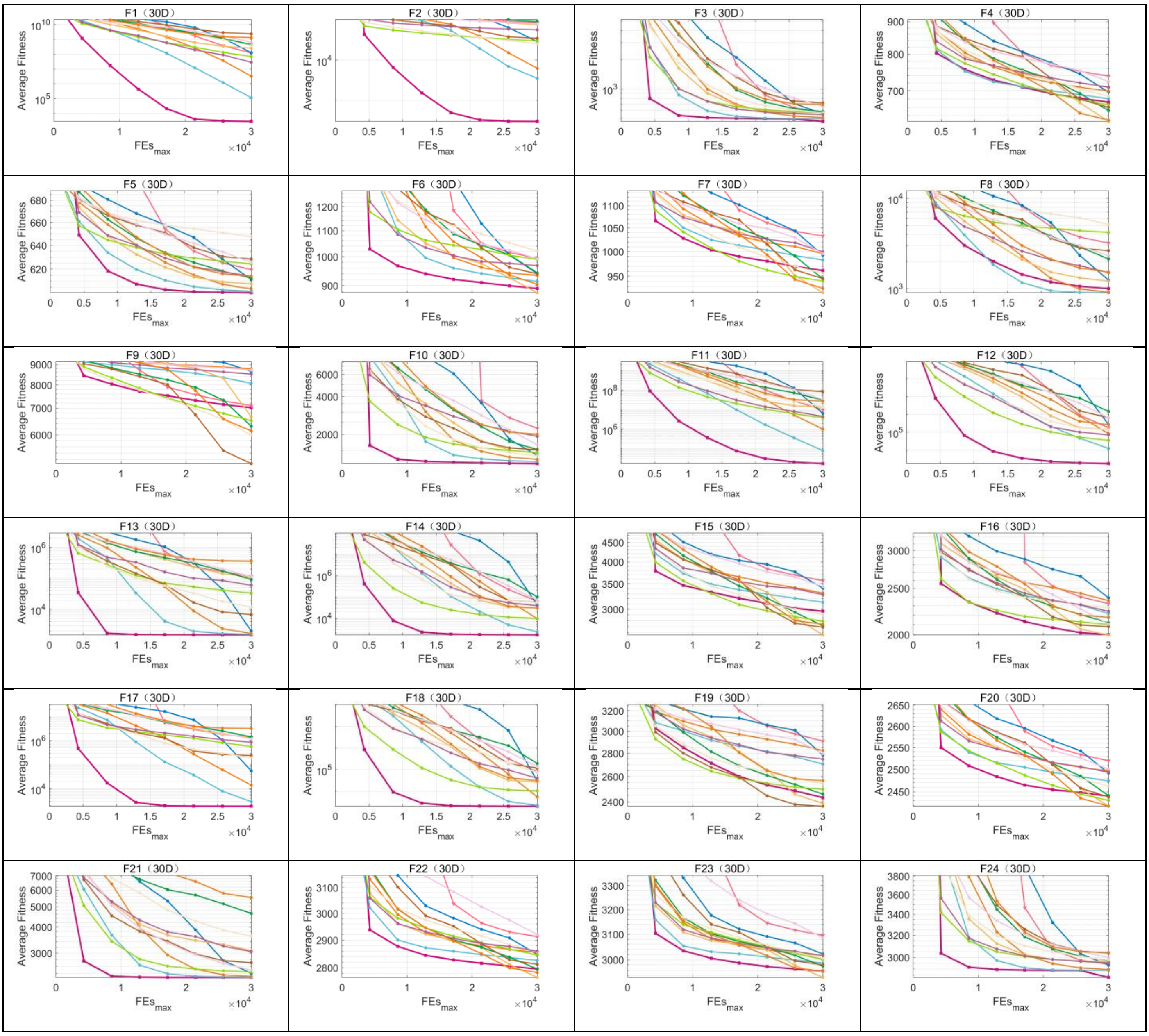

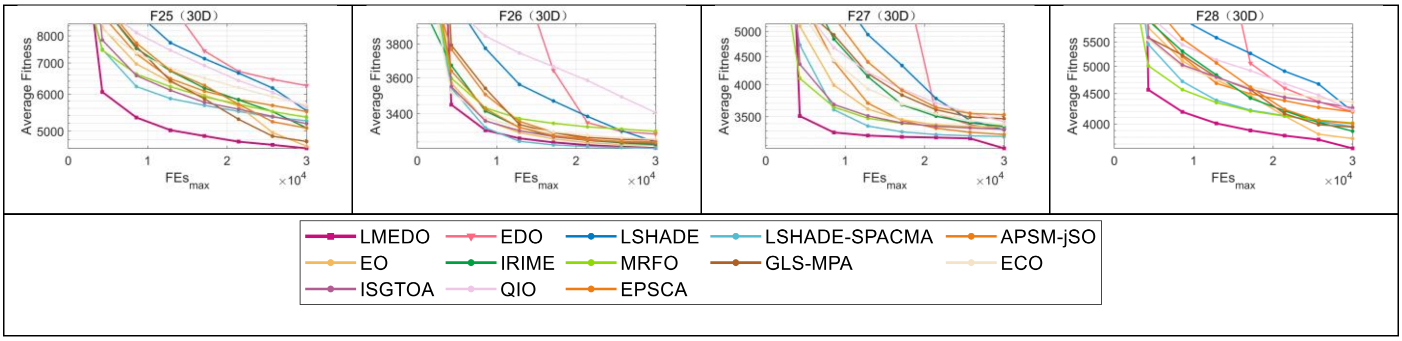

Figure B2. The convergence curves of LMEDO and comparison algorithms based on CEC2018 (30D)

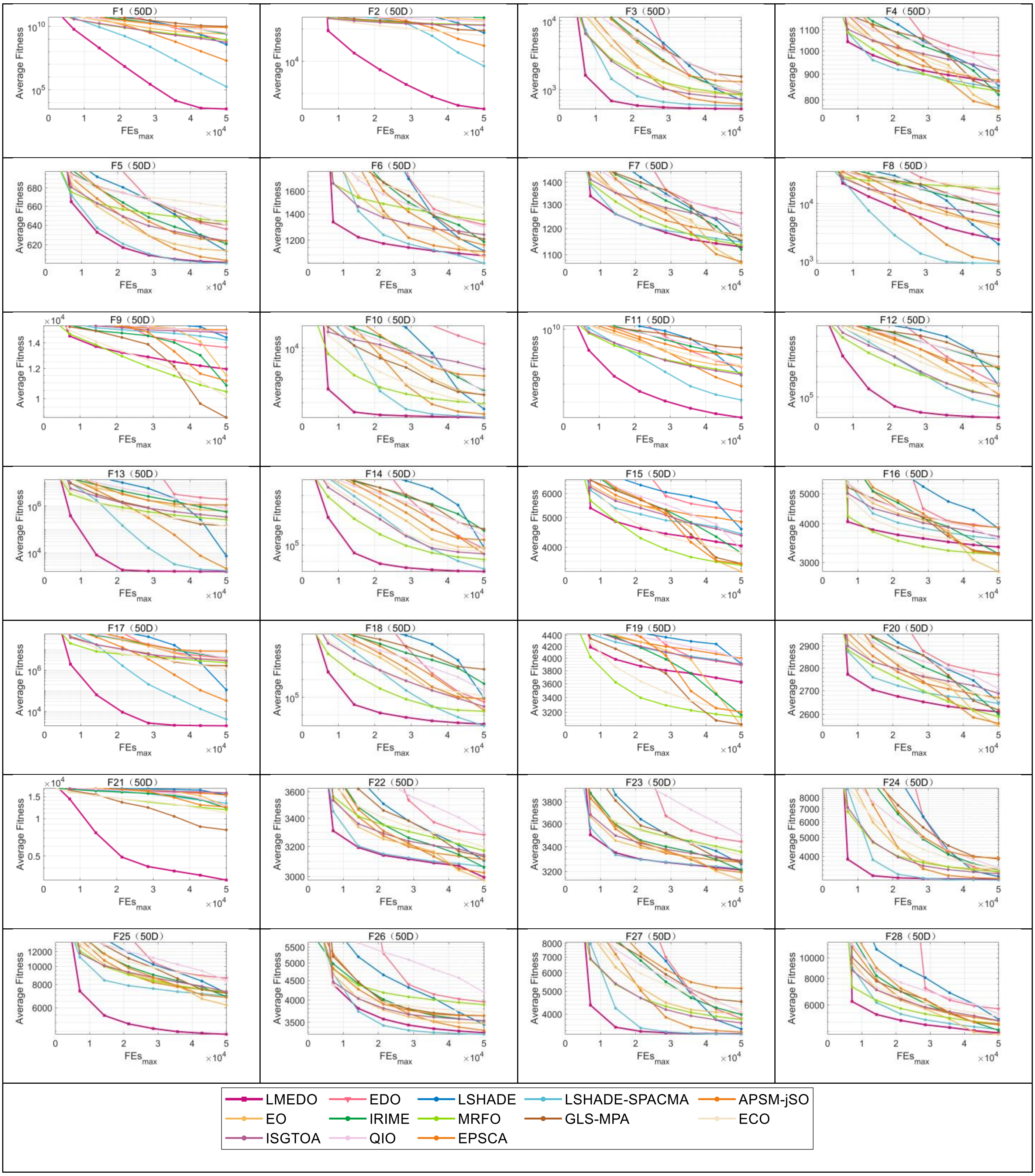

Figure B3. The convergence curves of LMEDO and comparison algorithms based on CEC2018 (50D)

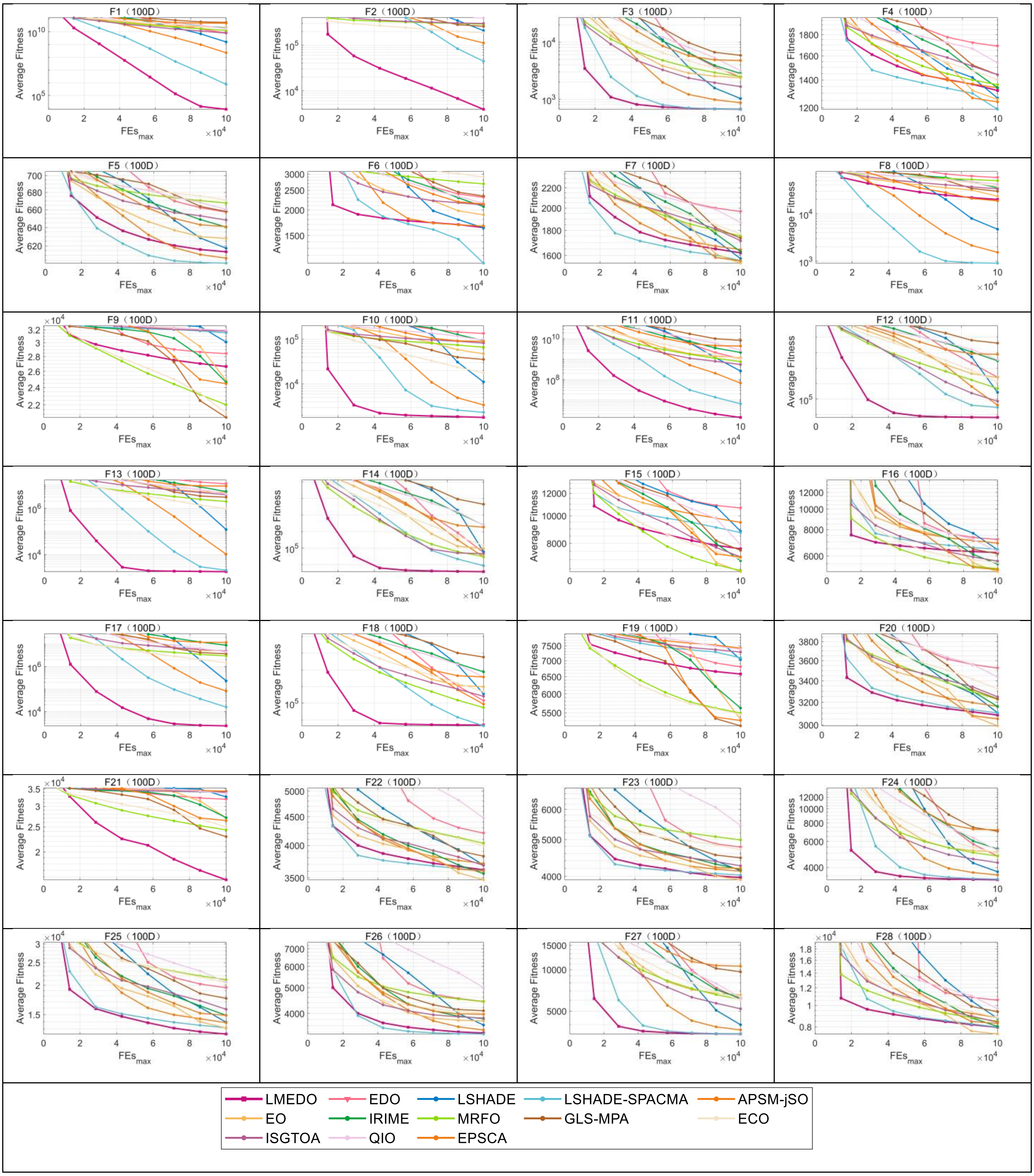

Figure B4. The convergence curves of LMEDO and comparison algorithms based on CEC2018 (100D)

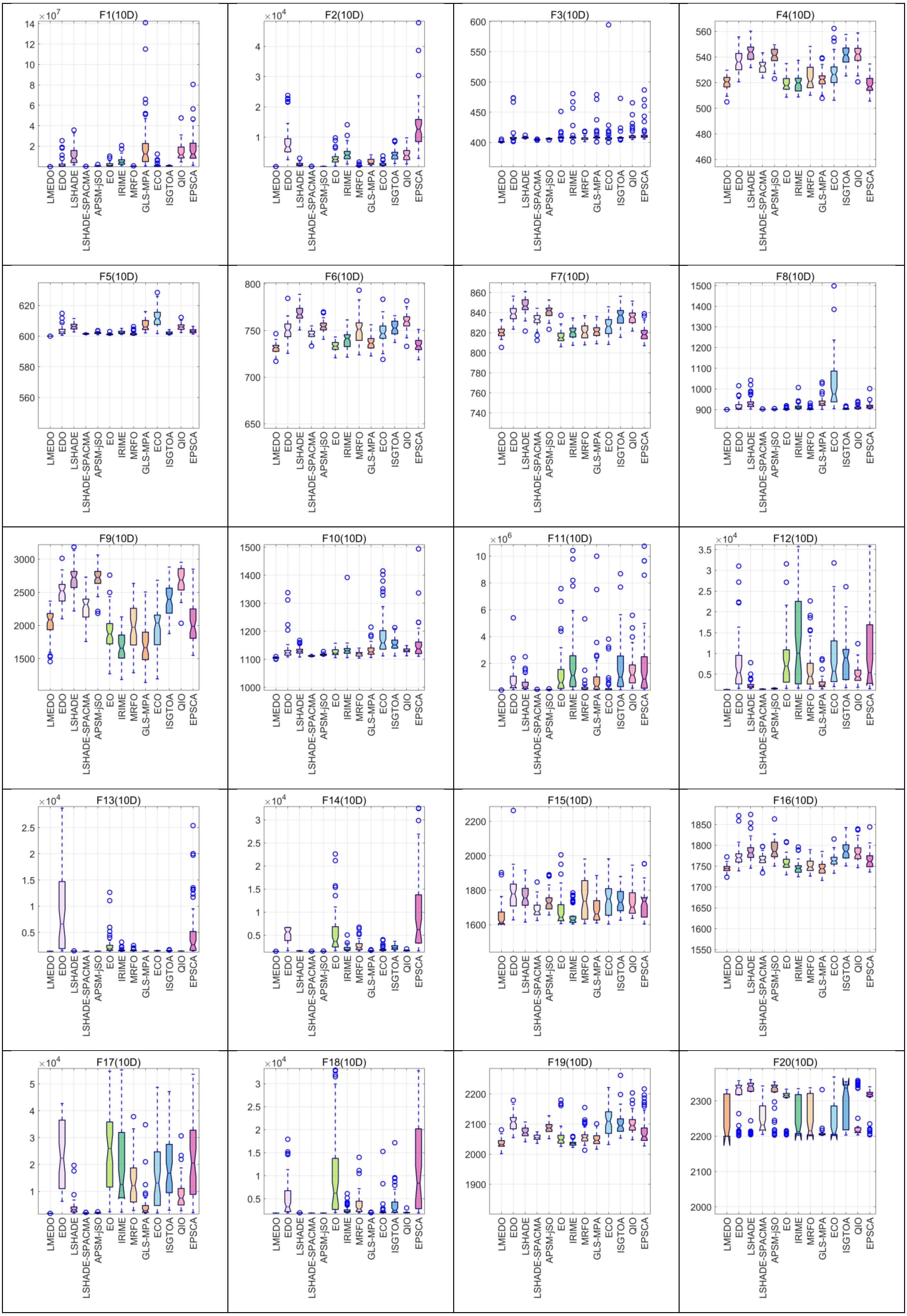
F1(10D)
F2(10D)
F3(10D)
F4(10D)
F5(10D)
F6(10D)
F7(10D)
F8(10D)
F9(10D)
F10(10D)
F11(10D)
F12(10D)
F13(10D)
F14(10D)
F15(10D)
F16(10D)
F17(10D)
F18(10D)
F19(10D)
F20(10D)
LMEDO
EDO
LSHADE
LSHADE-SPACMA
APSM-jSO
EO
IRIME
MRFO
GLS-MPA
ECO
ISGTOA
QIO
EPSCA

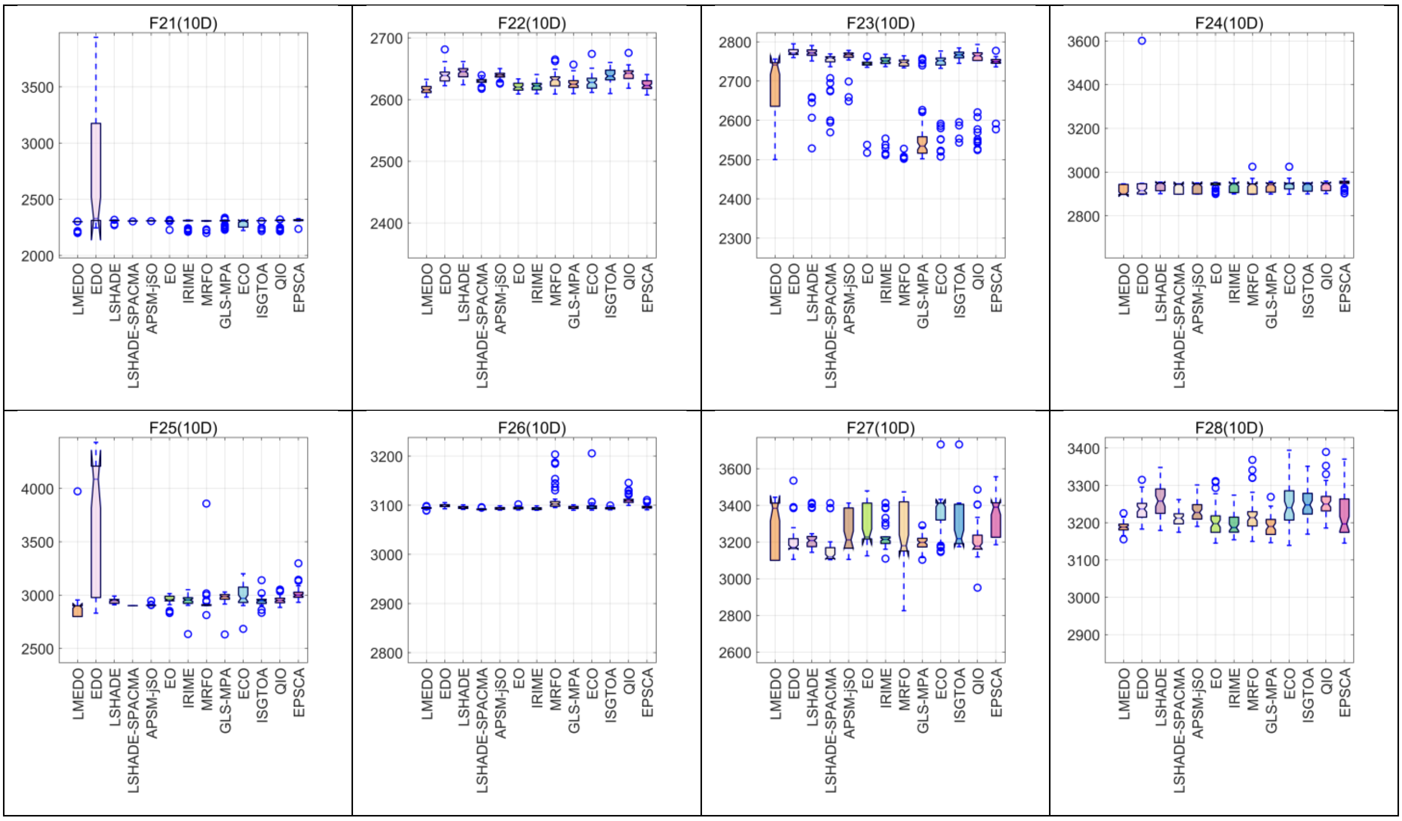

Figure B5. The boxplots of LMEDO and comparison algorithms based on CEC2018 (10D)

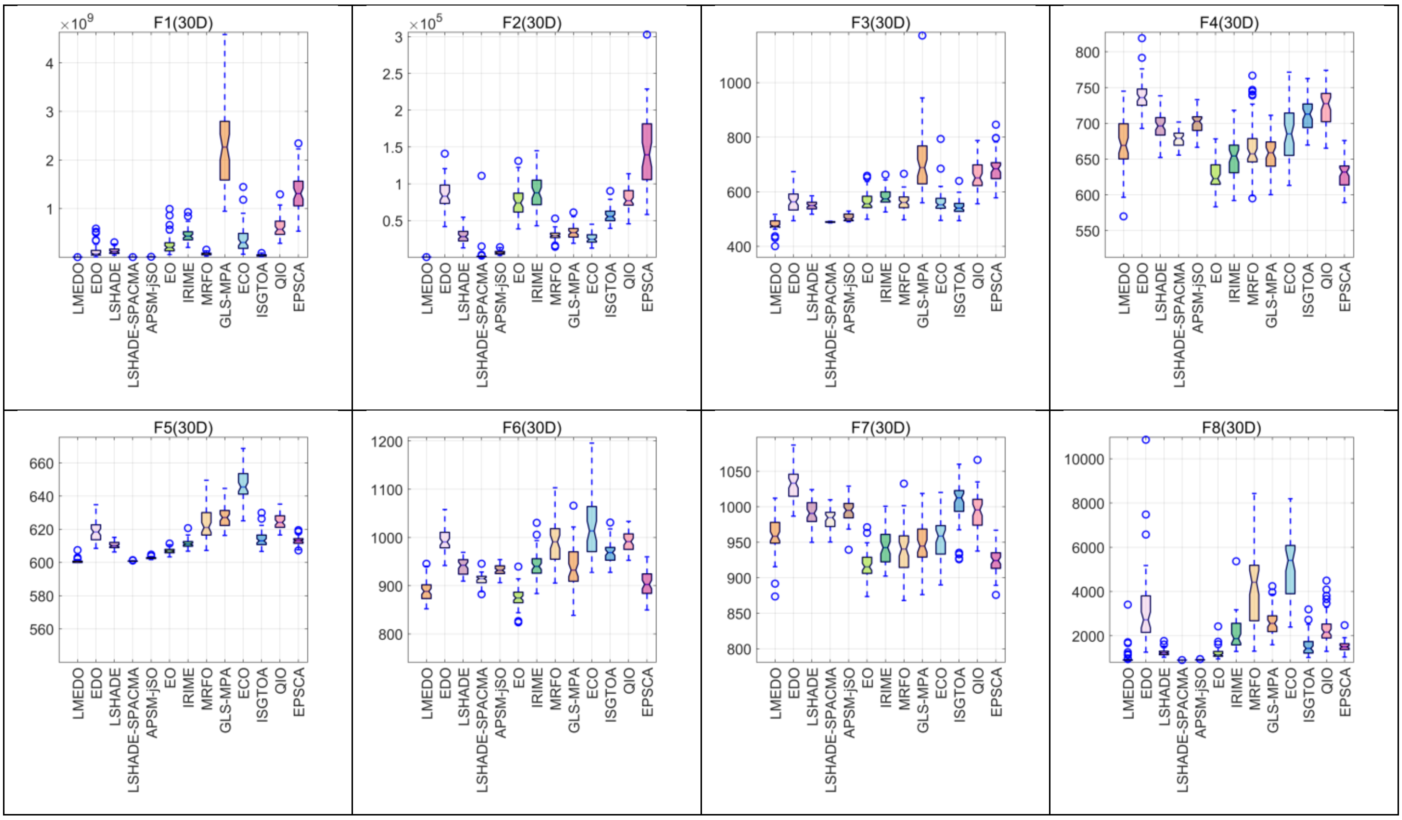

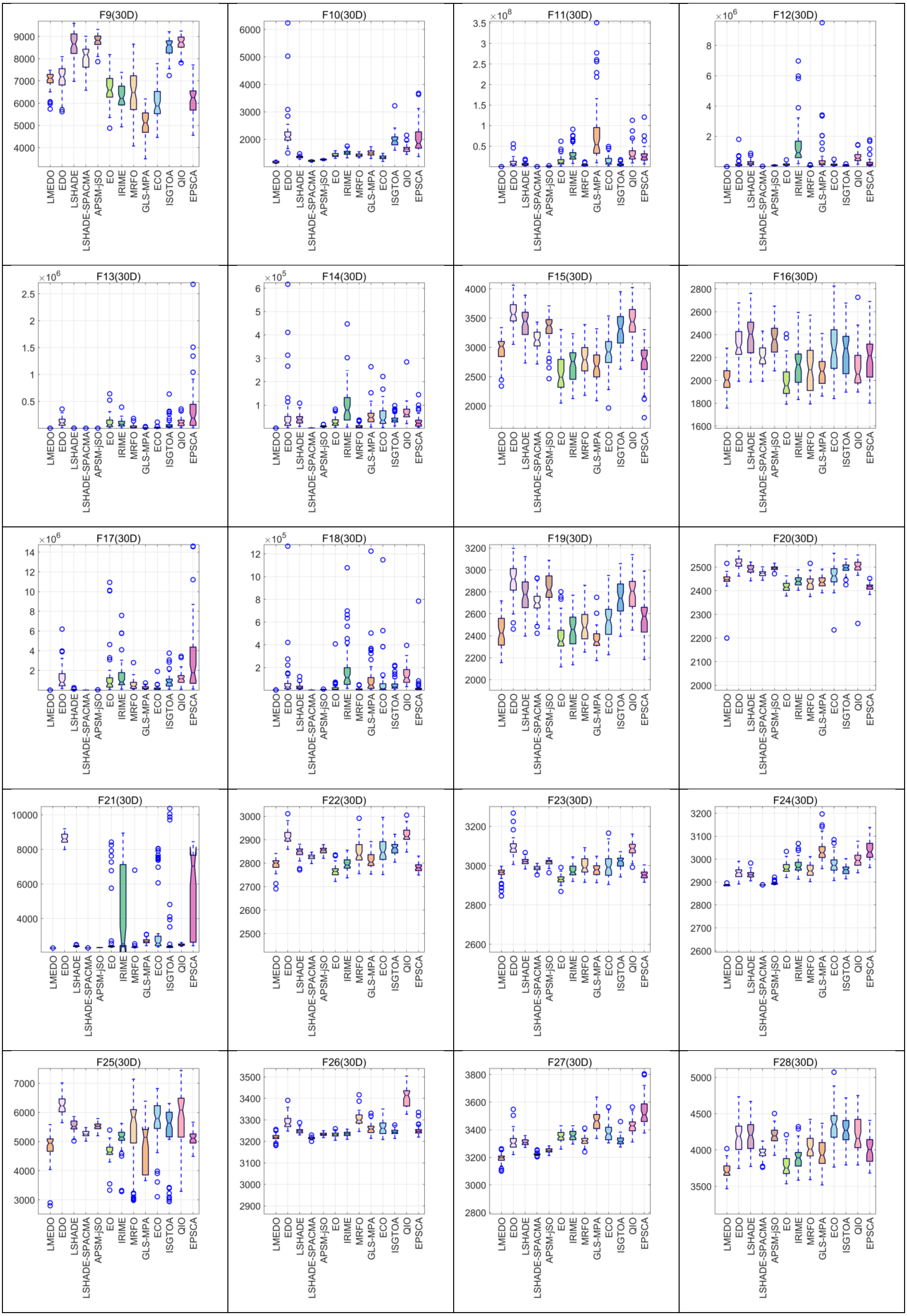
F9(30D)
F10(30D)
F11(30D)
F12(30D)
F13(30D)
F14(30D)
F15(30D)
F16(30D)
F17(30D)
F18(30D)
F19(30D)
F20(30D)
F21(30D)
F22(30D)
F23(30D)
F24(30D)
F25(30D)
F26(30D)
F27(30D)
F28(30D)
LMEDO
EDO
LSHADE
LSHADE-SPACMA
APSM-jSO
EO
IRIME
MRFO
GLS-MPA
ECO
ISGTOA
QIO
EPSCA

Figure B6. The boxplots of LMEDO and comparison algorithms based on CEC2018 (30D)

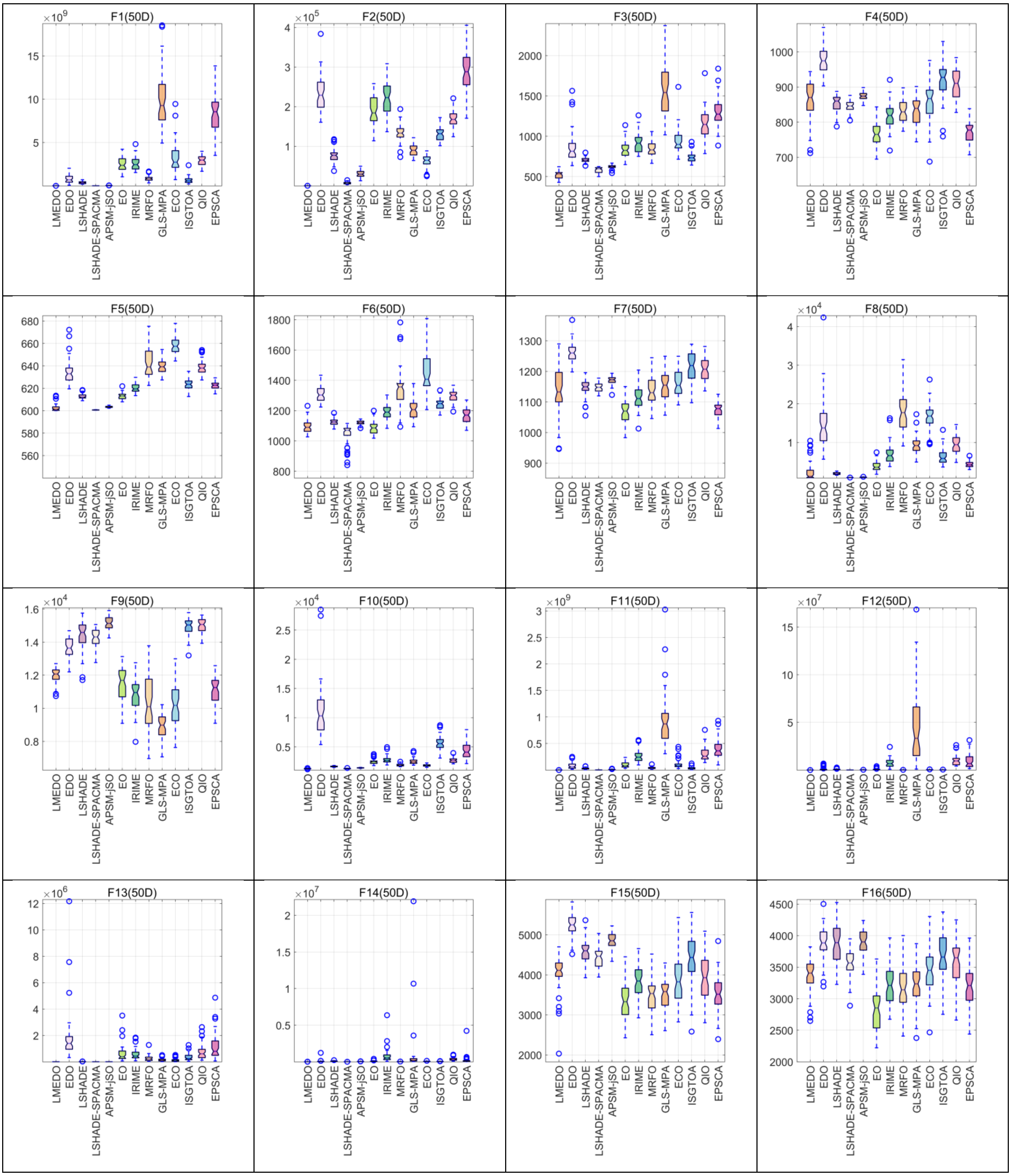

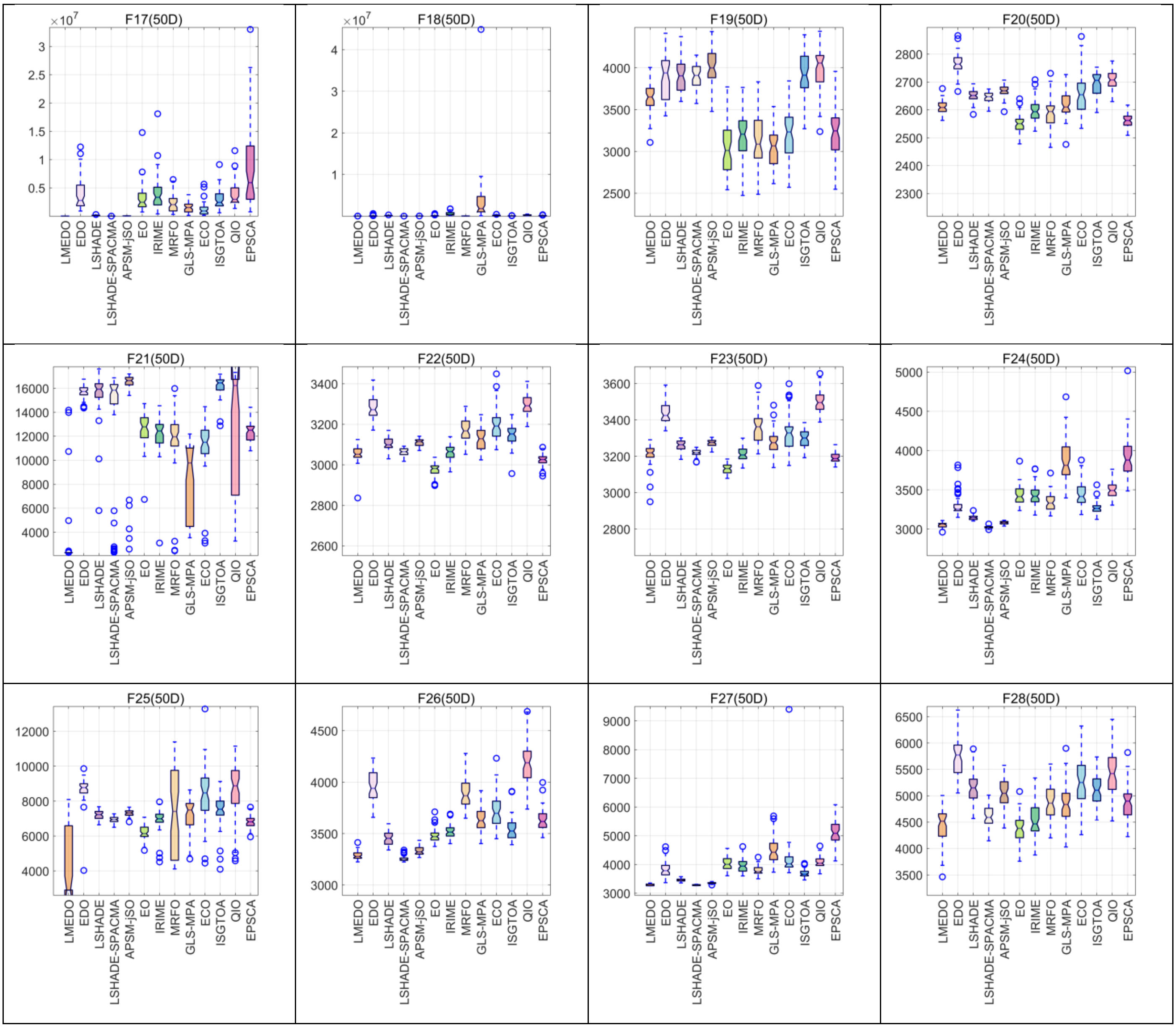

Figure B7. The boxplots of LMEDO and comparison algorithms based on CEC2018 (50D)

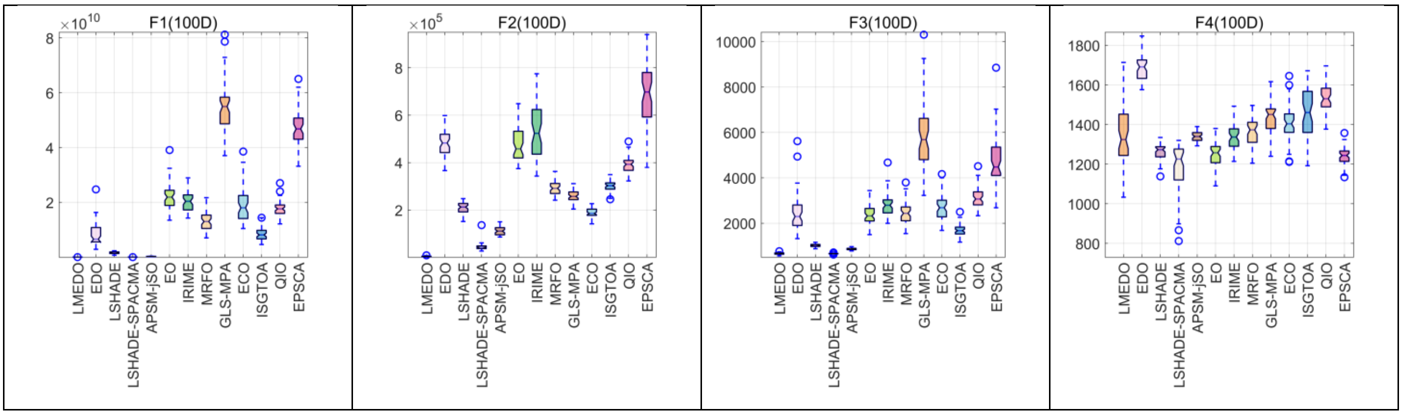

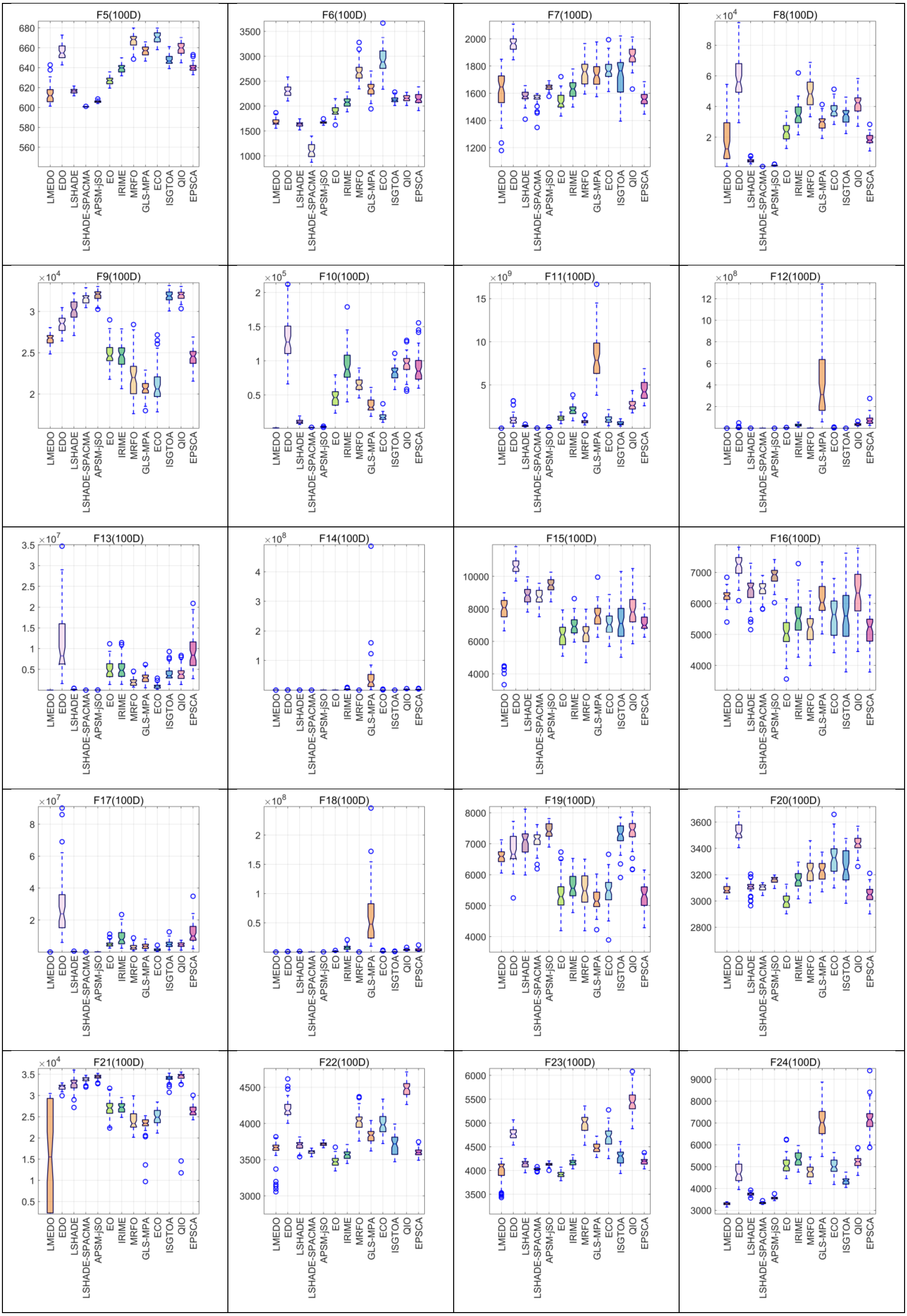
F5(100D)
F6(100D)
F7(100D)
F8(100D)
F9(100D)
F10(100D)
F11(100D)
F12(100D)
F13(100D)
F14(100D)
F15(100D)
F16(100D)
F17(100D)
F18(100D)
F19(100D)
F20(100D)
F21(100D)
F22(100D)
F23(100D)
F24(100D)
LMEDO
EDO
LSHADE
LSHADE-SPACMA
APSM-jSO
EO
IRIME
MRFO
GLS-MPA
ECO
ISGTOA
QIO
EPSCA

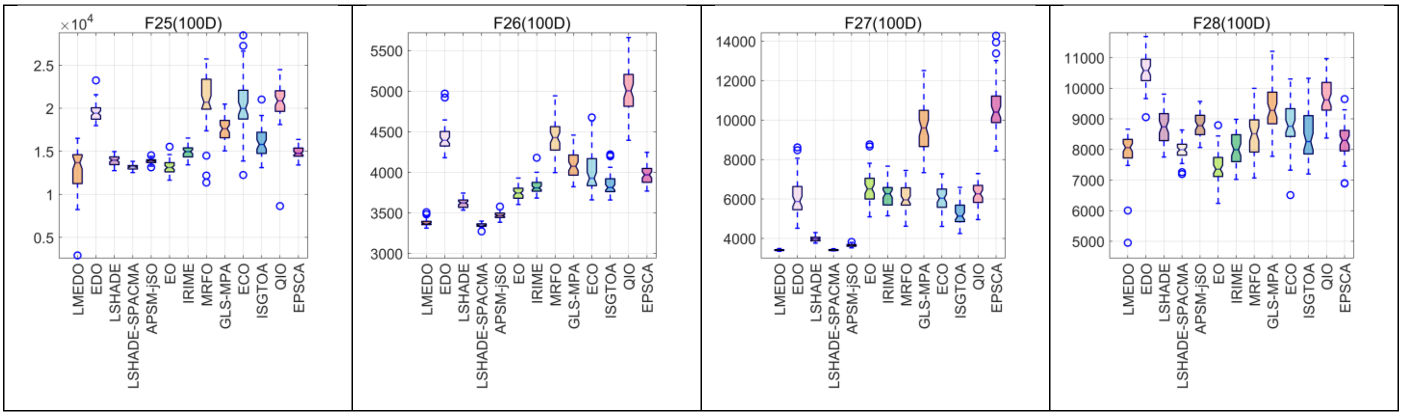

Figure B8. The boxplots of LMEDO and comparison algorithms based on CEC2018 (100D)
